# Effect of Ni Addition on the Solidification of Liquid Al and Solid Cu Diffusion Couples

**DOI:** 10.3390/ma18245689

**Published:** 2025-12-18

**Authors:** Vigneshwar Hari, Stuart D. McDonald, Xin Fu Tan, Kazuhiro Nogita

**Affiliations:** Nihon Superior Centre for the Manufacturing of Electronic Materials (NSCMEM), School of Mechanical and Mining Engineering (SoMME), The University of Queensland, Brisbane, QLD 4072, Australia; g.harikannan@uq.edu.au (V.H.); s.mcdonald1@uq.edu.au (S.D.M.);

**Keywords:** aluminium, copper, Al-Ni alloys, diffusion, microstructure, CALPHAD, solidification, brazing

## Abstract

Al-Ni alloys have a unique set of properties including high conductivity, high fluidity, good thermal stability, and reasonable strength. These properties are also needed for effective braze fillers, a novel application for Al-Ni alloys. A Cu substrate was reacted with pure liquid Al, and the resulting microstructure upon solidification was observed and analysed using scanning electron microscopy (SEM) and energy dispersive X-ray spectroscopy (EDS). This diffusion couple was compared with the diffusion couple between liquid eutectic Al-3at.%Ni and a Cu substrate. Several phases unique to the solidified liquid in the Al-Ni/Cu diffusion couple were observed, such as Al_7_Cu_4_Ni (τ), Al_3_(Cu, Ni)_2_, and Al_3_Ni. These microstructures were compared with a mathematical model based on Fick’s second law, as well as calculation of phase diagram (CALPHAD) modelling. The approximate calculated concentration profile of Cu in the liquid phase was validated against the microstructural observations and proved effective to explain the observed microstructural features. Liquid Al-3at.%Ni was found to limit the growth of the brittle Al_2_Cu (θ) phase during solidification by limiting Cu solubility in the liquid phase, which would be beneficial for use in dissimilar joints between Al and Cu.

## 1. Introduction

**Figure 1:**
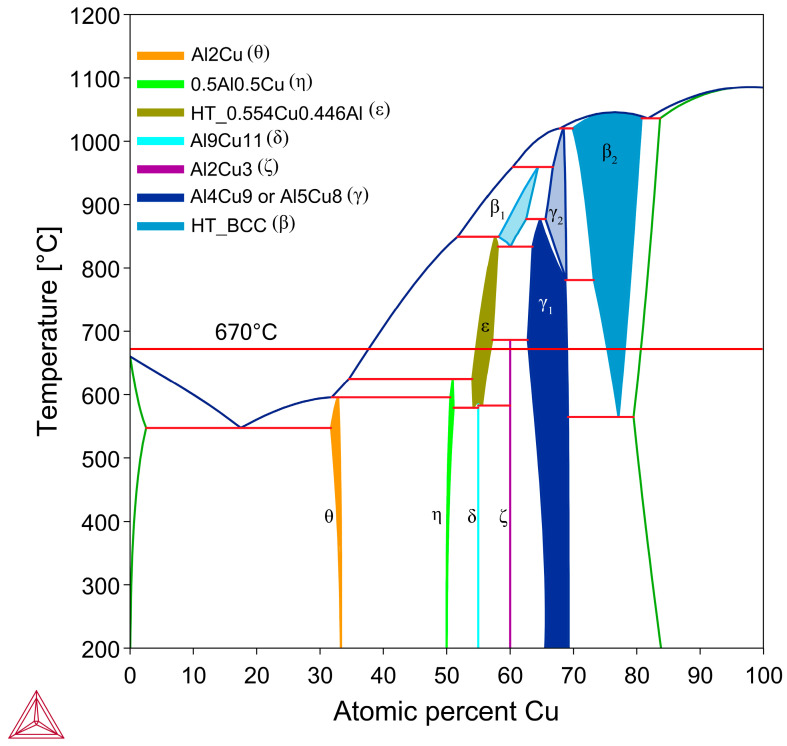
Al-Cu binary phase diagram with important room temperature stable phases labelled, as well as the process temperature for the samples in this study. Figure was produced using Thermo-Calc 2024b TCBIN TC Binary Solutions v1.1 Database [55].

Brazing is the process of joining two metal substrates together via the melting of a braze filler material (whose liquidus lies above 450 °C) to fill the contact surface via capillary action and solidification [1]. Brazing is widely used in many industries such as automotive, aerospace, heating, ventilation, and air conditioning (HVAC), and electronics [2]. While brazing is commonly used to join similar alloys with a shared primary element, dissimilar joints are of interest, as they can create parts with optimised properties harnessing different metals. Al/Cu joints are of interest due to the increasing prevalence of electric vehicles and battery technology, where the competing design factors of high conductivity and low weight can both be achieved with bimetallic parts composed of Al and Cu. Currently, the main method to join Al to Cu is to use friction stir welding (FSW), which involves using a rotating tool to heat and locally join the Al and Cu [3]. While this method has its advantages, it is limited in the geometries it can manufacture and the resultant joint has a complex microstructure, with several brittle Al-Cu intermetallic compounds (IMC) forming that affect strength and conductivity [3]. Currently, for brazing Al/Cu joints, the main options for filler alloys are Zn-Al alloys and Al-Si alloys [4,5,6]. Zn-Al braze filler has a low melting range that is easy to process samples with, between 381 and 570 °C depending on composition [7], and produces IMCs with up to 500 HV hardness in the diffusion zone [6,8]. The main limiting property of using Zn-Al filler alloys is that Zn has a low electrical conductivity of 27% IACS (International Annealed Copper Standard) (15.56 MS/m) [9], which would be detrimental for the high conductivity Al-Cu joints often try to achieve. Al-Si alloys have between 40 and 50% IACS (23.20–29 MS/m) [10], thus it is the most commonly used braze filler for Al-Cu joints. Si does not form IMCs with Cu in the Al- and Cu-rich part of the Al-Si-Cu system [5]; however, Si forms large lamellar crystals in the braze that have high hardness values of ~980 HV and are brittle [11]. The Al in Al-Si alloys also form Al-Cu phases with hardness values between ~660–920 HV that lead to brittle failure and crack formation [12]. The Al-Cu phase diagram in Figure 1 shows the main Al-rich equilibrium phase in the Al-rich section of the system is Al_2_Cu (*θ* phase). The *θ* phase was noted as being quite brittle [11], with a low fracture toughness [12], which leads to *θ* being the main crack initiation point in Al/Cu brazed joints with Al-Si [5,8,11,12,13].

Al-Ni alloys have had recent scientific interest as a possible new generation casting material. Several works have highlighted that alloys from this system have superior tensile strength compared to Al-Si alloys, even at elevated temperatures, especially after trace element inclusions [14,15,16,17,18,19,20]. The alloy is also a popular choice for directional solidification experiments, due to its highly faceted Al_3_Ni phase [21,22,23,24]. Al-Ni alloys are also commonly used when investigating the effect a magnetic field has on solidification [25,26,27]. Commercially, the main use for Al-Ni alloys is for easily castable (high fluidity [28,29,30]), high conductivity parts in electric vehicles [28]. Eutectic Al-6.1wt.%Ni (Al-3at.%Ni) has a conductivity value of 55.5% IACS or 32.19 MS/m [31], which is considerably higher than eutectic Al-Si [10], and only marginally lower than the ~61% IACS (35.96 MS/m) value of pure Al [28,32]. Al-Ni alloys have a unique eutectic morphology, which consists of a matrix of an Al phase with needle-like Al_3_Ni crystals embedded into it [29]. Given these properties, it was theorised that Al-Ni eutectic alloy could be used as a braze filler for dissimilar Al to Cu joints. There are, however, several steps required to investigate the viability of Al-Ni alloys before using them in a brazed joint. The alloy has a high melting temperature of ~640 °C [29,33,34], and, since this value is close to the melting temperature of pure Al (660 °C), it could cause issues during the brazing Al to Cu joints. To try and modify this, in the author’s previous work, it was investigated if a Sr addition would lower the eutectic temperature of Al-Ni alloys [33], as a similar effect was found in Al-Si alloys [35,36]. Sr was found to not affect the eutectic temperature of Al-Ni alloys.

The authors propose that Al-Ni alloy can be used to create brazed Al/Cu joints with improved microstructural features. A study by Xiong et al. [37] looked at the effect of a Ni interlayer on solid-state Al/Cu diffusion couples. The study found that this improved the strength and lowered the hardness of the Al/Cu solid-state diffusion joint. Similar results were found when Ni was used for Al/Cu friction welds [38]. The addition of 1wt.%Ni to Al-6.5wt.%Si-20wt.%Cu (0.52at.%Ni to Al-at.%7.12Si-at.%9.7Cu) braze filler alloy (for Al to Al brazes) was found to be beneficial in improving mechanical properties of the braze by transforming some of the *θ* phase into Al_3_(Cu, Ni)_2_, while also modifying the morphology of the *θ* phase from bulky crystals to lamellar Al-Al_2_Cu eutectic [39]. Works by Furuya et al. [40,41] looked at comparing laser welded joints of Al/Cu to Al-Ni/Cu [40], and using Al-Ni alloys at various compositions as a filler metal during Tungsten inert gas (TIG) arc brazing Al-Ni to Cu [41]. These studies found that the addition of Ni reduced the layer thickness of the brittle crack prone *θ* layer, while also forming a new Al_7_Cu_4_Ni (τ) layer between the interface of *θ* and Al_4_Cu_9_ (γ phase) phases [40] that strengthened the joint. The additional τ particles also strengthened the *θ* layer [41].

Given these promising results, it was hypothesised that eutectic Al-Ni alloy would make for a good braze filler alloy for joining Al to Cu. The diffusion behaviour between a high purity Al substrate and Al-Ni braze filler is reasonably straightforward and easy to understand given the low Ni concentration and parallels with diffusion zones produced between Al and Al-Si braze filler [13]. However, the diffusion behaviour between Al-Ni braze filler and the Cu substrate during time scales present in furnace brazing (between 1 and 5 min [1,13]) is not well known. It is crucial to understand this reaction to help evaluate Al-Ni braze filler’s viability for joining Al to Cu. Investigations of the Al-Cu-Ni system showed that several binary and ternary phases can form during casting [42,43,44,45,46,47]. However, given that the Cu solute profile in the liquid is not constant in a diffusion couple, the final microstructure of the Al-Ni/Cu couple is uncertain. Furthermore, many previous works have investigated the diffusion reaction between liquid Al and solid Cu [48,49,50,51,52,53,54]; however, the addition of Ni to the liquid Al is a novel variation. Ni addition to filler Al metal in previous welding works [40,41] led to a reduced amount of *θ* phase in the welded joint between Al and Cu. If similar reductions in the thickness of the *θ* phase layer can be replicated, it would likely lead to improved mechanical properties of a fully brazed Al/Cu joint [11,12], and the improved conductivity of the joint. In this study, diffusion between Al/Cu couples (henceforth, all mentions of Al/Cu and Al-Ni/Cu couples imply that the reaction is between liquid Al or Al-Ni and solid Cu), will be compared with Al-Ni/Cu couples at a fixed temperature and several time scales common in furnace brazing. This work aims to investigate the macroscopic patterns of phase formation during solidification, and, specifically, which phases form at which distances from the Cu substrate and how this reflects Cu diffusion over time. The microstructures and modelled diffusion behaviour will be compared with ThermoCalc modelling, and theoretical diffusion curves of Cu in the liquid, based on Fick’s laws, will be created to discern if scientific modelling of diffusion behaviour within the samples match observations of its solidification, and to specifically discern the effect of the Ni addition.

## 2. Materials and Methods

**Figure 2:**
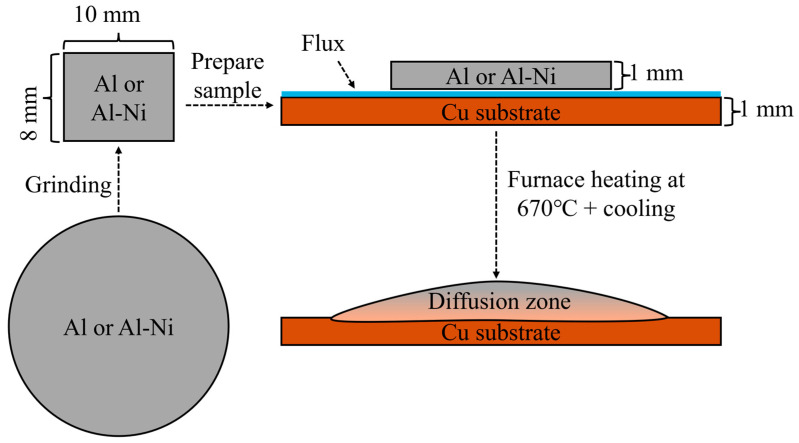
Summary of sample preparation process. The sample is initially cut into circular discs, then ground to 10 × 8 × 1 mm pieces. This is placed on a Cu substrate with flux and heated in the furnace and then removed and quenched to form the final sample.

Samples for the liquid layer were first created using an induction furnace. Nominally pure AA196 aluminium (99.96% pure Al) was cast into cylindrical ingots with a boron nitride coated steel mould with a diameter of 20 mm to make an ingot. AA196 was mixed with 99.97% pure Ni to produce ~Al-3at.%Ni (all percentages in at.% unless otherwise stated) and cast using the same process. The cylindrical ingots for both compositions were then cut into ~2–3 mm thin discs. These were then ground down into roughly 10 × 8 × 1 mm (±0.3 mm) rectangular prisms weighing approximately 250 mg for the Al/Cu samples and 270 mg for the Al-3Ni samples. These prisms were then cleaned with an Al_2_O_3_ abrasive. Then, 20 × 12 × 1 mm (±0.1 mm) Cu substrates (99.99% pure), which were laser cut from a larger sheet of Cu, were also cleaned with the Al_2_O_3_ abrasive. Comweld Aluminium Flux^TM^ (proprietary mix of KCl, LiCl, NaCl, and NaF) was applied on one side of the Cu substrates. The unmelted Al alloys were placed on the fluxed side of the Cu substrates, and the couples were placed in an oven at 110 °C for 10 min to remove moisture. Brazing was carried out in a Carbolite STF 16-180 Single-Zone Tube Furnace (Sheffield, UK), with an inner diameter of 50 mm. With an open furnace door, argon at a constant flowrate of 10 L/min was supplied through the tube furnace and this flow assisted with the removal of gasses from the flux and to reduce the presence of oxygen during the diffusion reaction. The internal temperature of the furnace was set to ~610 °C and monitored by a K-type thermocouple placed on top of the sample. The sample was placed on top a refractory brick and inserted into the hottest section of the furnace. The sample was then heated for 15 min to allow the flux to evaporate completely (the melting temperature of the flux is ~540 °C). The furnace temperature was then increased to 670 °C. Once the sample reached 10 °C below the melting temperature of the unmelted liquid (640 °C and 660 °C for the Al-Ni/Cu couple and the Al/Cu couple, respectively), the thermocouple was removed. The sample was visually monitored until melting of the entire piece of Al or Al-Ni was observed, at which point a timer was started. After the desired times were achieved (15, 30, 60, 90, and 1800 s), the sample was slowly removed from the centre over 5–10 s. It was then air cooled near the opening of the tube furnace at a cooling rate of ~6–12 °C/s (an additional sacrificial sample was measured by a 2.5 mm diameter K-type thermocouple to produce this value). All samples were controlled to similar thermal conditions within the furnace and during cooling. A summary of the sample creation process is shown in Figure 2. The naming convention for each sample is the composition of the Al-based substrate followed by the time above liquidus temperature (i.e., Al 15s would be Al/Cu couple after 15 s of observed liquid Al phase present).

After the samples were created, they were mounted into epoxy with the weight ratio 25:3:3 epoxy-resin-to-hardener-to-graphite-powder to create conductive scanning electron microscopy (SEM) compatible moulds. Since the liquid Al/Al-Ni tended to form a spherical cap once melted, the samples were roughly ground with the aim to display the thickest cross-section of this spherical cap. Samples were then polished using standard metallographic techniques. These samples were imaged with a Hitachi TM1000 SEM (Tokyo, Japan), while energy dispersive X-ray spectroscopy (EDS) was performed using a Hitachi TM3030 SEM/EDS (detector Quantax70 EDS). Mosaic SEM images of the microstructures were created from imaging a vertical section at the thickest (largest distance between Cu substrate and edge of the solidified liquid phase) part of the sample (or near the thickest to avoid porosity/scratches) and using ImageJ Fiji’s pre-installed stitching macro to join the micrographs. EDS spot analysis was performed at least 5 times for each phase, and the averaged compositions are reported. Measurements of layer thicknesses (specifically the *θ* phase and S/L interface migration) were made using the Inter-edge Distance Macro version 3 (Supplementary Code S1) for ImageJ Fiji (https://forum.image.sc/t/imagej-macro-to-measure-distance-between-two-user-drawn-lines-edges-version-3/110803, accessed on 23 June 2025) [56]. This macro uses a perpendicular line between two user-drawn lines to measure the distance between two lines. The code, results, and images with layer thickness measurements are available as Supplementary Materials. All CALPHAD was performed on Thermo-Calc 2024b using the TCAL7 Al Alloys v7.1 and TCBIN TC Binary Solutions v1.1 databases [55,57]. Finally, numerical calculation of the Cu concentration profile within the samples was performed using Python 3.12 (Supplementary Codes S2 and S3).

## 3. Results

### 3.1. Thermo-Calc Simulation and Phases Present

**Figure 3:**
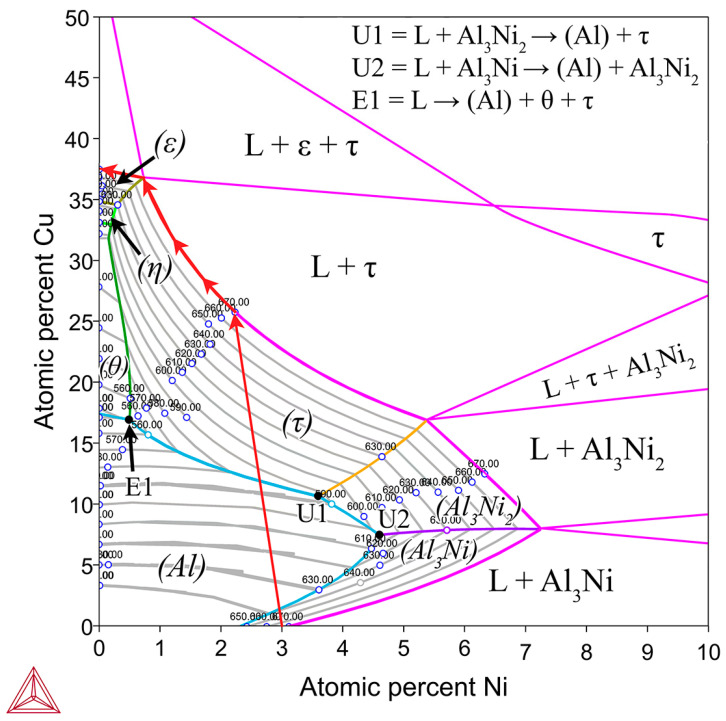
Isothermal section of the Al-Cu-Ni phase diagram at 670 °C shown in pink. For the composition region where only L phase is present, a liquidus projection for this region is overlayed with isothermal tie-lines every 10 °C shown as grey lines [57]. This is implemented to show the primary phases (labelled in brackets) that form during solidification of liquid in the Al-Ni/Cu couple based on the local composition of the L phase. Initially, the entire L phase has a composition of Al-3Ni, then, as Cu dissolves into the L phase, local compositions change following the red arrows, until all the Ni precipitates out of the L phase as τ or diffuses into the Cu couple. Invariant reaction points are shown with U1, U2, and E1, with the equations shown in the top right of the figure [44]. Note, there is a slight discrepancy between TCAL7 and the literature, as it states the Al-Ni eutectic point is at 2.4at.%. This work and other works in the literature use 2.9–3.1at.% Ni as the eutectic point for the Al-Ni system [16,29,33,34]. Modelling was performed using ThermoCalc 2024b using TCAL7 Al-based Alloy Database.

**Figure 4:**
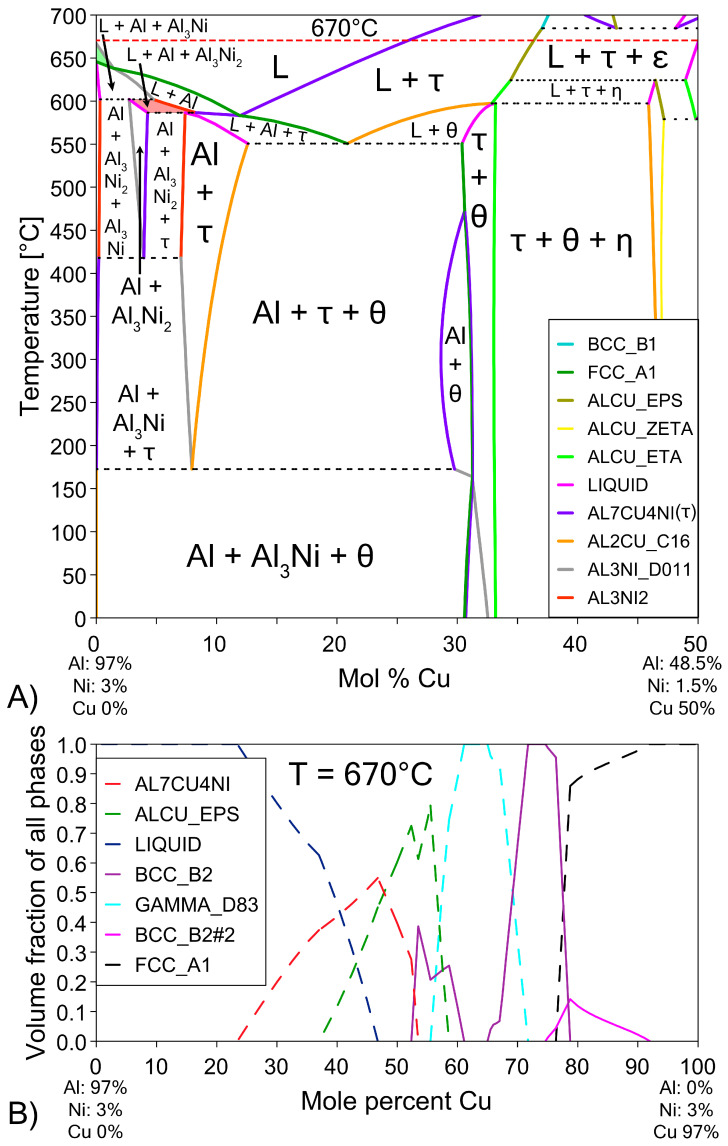
(**A**) Pseudo-binary phase diagram of the (100 − x)(Al-3Ni)-(x)Cu system. This diagram shows the equilibrium phases that form at different temperatures as more Cu diffuses into the L phase and the ratio of Al-3Ni to Cu changes [57]. The regions of the phase diagram corresponding to the phases L + Al + Al_3_Ni_2_ are highlighted in red and L + Al_3_Ni is highlighted in green for future discussion. (**B**) Pseudo-binary single axis diagram showing volume fraction of various phases at 670 °C (red dashed line in Figure 4A). Ni is fixed at 3% to highlight formation of β phase.

The possible phases that form in the diffusion couples were investigated using the Al-Cu phase diagram (Figure 1), as well as the isothermal Al-Cu-Ni phase diagram at 670 °C, shown in Figure 3. Given the previously stated cooling rate, while it was faster than equilibrium conditions, it was not fast enough to cause significant amounts of non-equilibrium phases to form [58,59]. The lack of non-equilibrium phases was also noted in later observations. The liquidus projection (for all compositions that are entirely liquid phase henceforth shall be referred to as L phase) at 670 °C, was projected onto the isothermal diagram. A pseudo-binary phase diagram of the Al-Cu-Ni system is also plotted in Figure 4A. Its *x*-axis starts at a composition of Al-3Ni and progressively replaces Al-3Ni with Cu, showing all equilibrium phases that form between 0 and 700 °C. Note, while equilibrium phases at room temperature are shown, generally for Al alloys, these phases do not reflect the observed microstructure as the kinetics of these solid-state reactions are generally very slow. Phases present in Figure 1, Figure 3 and Figure 4 are summarised in Table 1. There are two main Al-Cu-Ni ternary phases identified. These phases are Al_7_Cu_4_Ni (τ) and Al_3_Ni_2_, or, since there is significant Cu solubility in this phase, it is also referred to as Al_3_(Cu, Ni)_2_ in this work.

### 3.2. SEM and EDS Analysis

#### 3.2.1. Overview of Phase Formation in the Diffusion Couples

**Figure 5:**
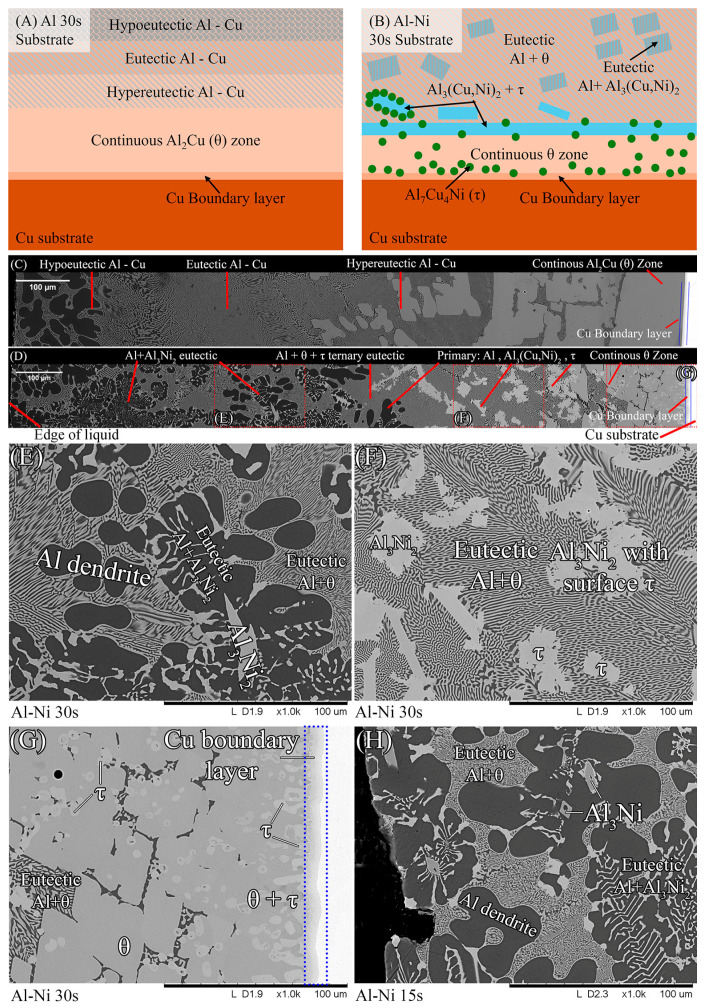
(**A**) Schematic of the various microstructure layers found in the Al 30s sample. (**B**) Corresponding schematic showing microstructures and layers found in the Al-Ni 30s sample; (**C**) mosaic SEM micrograph of the Al 30s sample showing the layers described in 4A, rotated 90° anticlockwise. (**D**) Mosaic SEM micrograph of the Al-Ni 30s sample showing the layers described in 4Bs, also rotated. (**E**–**G**) High-magnification SEM images of regions outlined in dotted red sections of (**D**) with phases labelled. (**H**) High-magnification SEM image of Al-Ni 15s sample showing region next to the edge of the L phase with phases labelled.

To aid in the analysis and discussion of the complex microstructure present in the samples, Figure 5A,B were created as general guides for simplifying the microstructures into distinct layers/regions for the sake of discussion/comparison. Figure 5A shows a schematic summarising the microstructures found in the Al 30s sample. The corresponding macroscopic image is shown in Figure 5C. Note that in this work, unless otherwise stated, all SEM images are rotated 90° anticlockwise compared to reality. Figure 5A,C show, for the Al 30s sample, observing from the surface of the L phase toward the Cu substrate, the layers reflecting the expected phases/microstructures that form throughout the binary Al-Cu alloy system, shown in Figure 1 [60]. This was followed by a region dominated by primary θ phase, which solidified as a large continuous layer. This region is dubbed the “continuous Al_2_Cu (θ) layer”. This region is of major importance, as large *θ* crystals are what contribute to the general brittleness of Al/Cu brazed joints [5,12]. Finally, there was the Cu boundary layer. The Al-rich edge of this layer also marks the location of the solid/liquid (S/L) interface during the diffusion reaction, as all phases to the left of it (vertically higher in the sample) formed upon the liquid cooling or precipitated out of the L phase, while all phases below it were solid and never melted. This layer was composed of several solid phases that formed due to Al diffusion into solid Cu. For the 30 s sample, this layer was only around 10 μm thick in the Al-Ni 30s sample.

**Figure 6:**
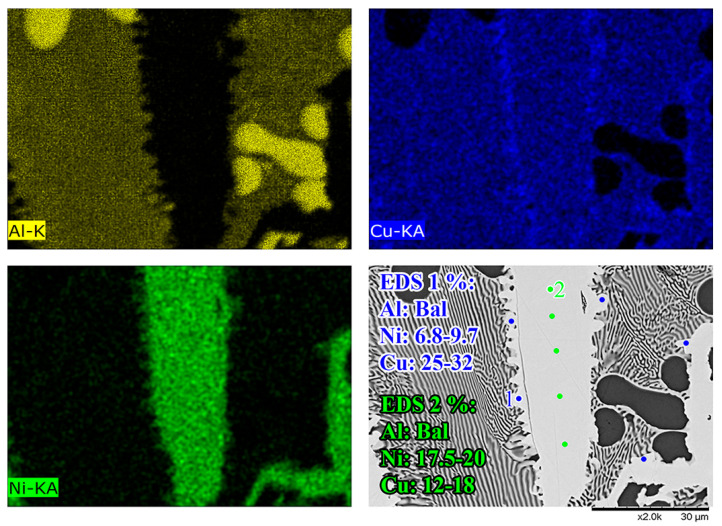
High-magnification SEM-EDS micrographs of a Al_3_Ni_2_ in the Al-Ni 15s sample. From the EDS maps and point measurements, it shows that the relatively faceted Al_3_Ni_2_ crystal (EDS 2) has small τ formations on its surface (EDS 1) due to a quasi-peritectic reaction.

Figure 5B,D show the Al-Ni 30s sample and its characteristic microstructure. Figure 5E–G show high-magnification images of the sections demarcated with red dots in Figure 5D. Starting from the edge of the L phase to the Cu substrate, the first microstructural region was dominated by Al dendrites with Al_3_Ni_2_ in-between the dendrite arms, or with eutectic Al + Al_3_Ni_2_ surrounding the Al dendrites [45]. The eutectic Al + Al_3_Ni_2_ parts of the microstructure form in the red highlighted region of Figure 4A and along the liquidus slope joining U2 to U1 in Figure 3. Some large, possibly primary, Al_3_Ni_2_ crystals can also be found. The remaining space between Al dendrites was composed of eutectic Al + θ. This region is highlighted in Figure 5E,F. The volume fraction of the Al dendrites decreased as the distance from the S/L interface decreased, until no Al dendrites were observed. This marks the start of the next section, where large, likely primary, Al_3_Ni_2_ crystals were found. These crystals tended to be “blocky” or plate-like in shape with roughly straight edges. There were also large clumps of agglomerated τ phase found in this region. The remaining volume was composed entirely of eutectic Al + θ. There were some brightly coloured crystals that had the plate-like shape of the Al_3_Ni_2_ crystals, but with bumpy surfaces like that of τ phase. The EDS of one of these crystals from the Al-Ni 15s sample is shown in Figure 6. It highlights that these crystals are in fact Al_3_Ni_2_ crystals that underwent the quasi-peritectic reaction: L + Al_3_Ni_2_ → Al + τ at 586 °C [42,43,44]. Figure 6 shows that under SEM observation, the Al_3_Ni_2_ and τ phases cannot be distinguished via brightness, due to both phases having Al/Cu, Ni ratios of ~3:2 (Cu and Ni are combined as they have nearly identical atomic numbers). Thus, EDS was needed to ascertain the Cu/Ni ratio to distinguish between these phases. Figure 6 shows that since τ is Cu-rich, its Cu/Ni ration is roughly 4:1, while Al_3_Ni_2_ has a roughly 1:1 ratio of Cu/Ni, hence why this phase can also be referred to as Al_3_(Cu, Ni)_2_. Another observation is, that closer to the S/L interface, there are more globular τ phase, and less blocky Al_3_Ni_2_ phase.

There is a ‘line’ of primary Al_3_Ni_2_ + τ phase that demarcates the transition to the next region. This region (shown in Figure 5G) started almost entirely composed of eutectic Al + θ before transitioning to the θ dominated area, until θ + small globules of τ composed the continuous θ layer. This region ended with the S/L interface, after which the Cu boundary layer is found. The frequency of the τ globules increased closer to the S/L interface, with some τ crystals even found attached to the S/L interface. This region must have a Cu concentration above the eutectic Al + θ + τ point of 20.8% Cu (see Figure 4A), due to the presence of θ crystals. While Figure 5D–G highlight most of the phases and relevant morphologies found in the Al-Ni/Cu samples, the Al-Ni 15s sample did have one additional unique phase. The primary Al_3_Ni phase is shown in Figure 5H. This phase only forms when the local Cu% is lower than 1.12%, the green highlighted region in Figure 4A. Figure 5H also highlights the different morphologies that eutectic Al + Al_3_Ni_2_ can present as. In this figure, the phases have a distinct ‘fishbone’ morphology [45]. This morphology coarsens with increased local Cu concentration, which occurs either with increased diffusion time or lower distances from the S/L interface.

#### 3.2.2. Evolution of Microstructures over Increasing Diffusion Times

**Figure 7:**
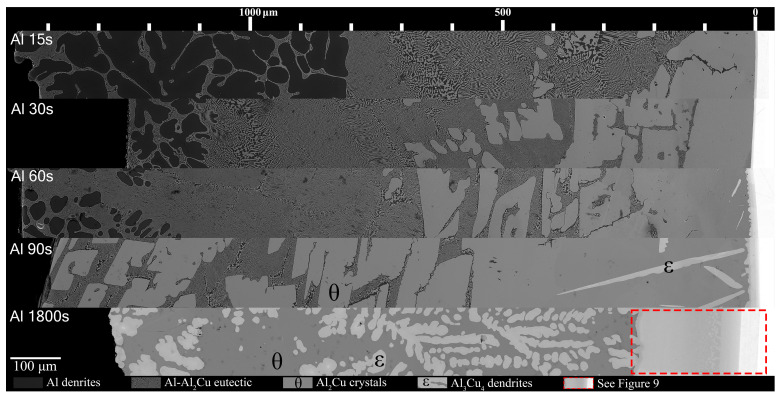
Mosaic SEM micrographs of Al/Cu couples ordered in increasing time above liquidus. θ phase as well ϵ phase that forms at longer diffusion times are labelled. The scale at the top starts at approximately the edge of the Cu phase in the substrate, which is roughly also the location of the S/L interface for Al 15–90s.

**Figure 8:**
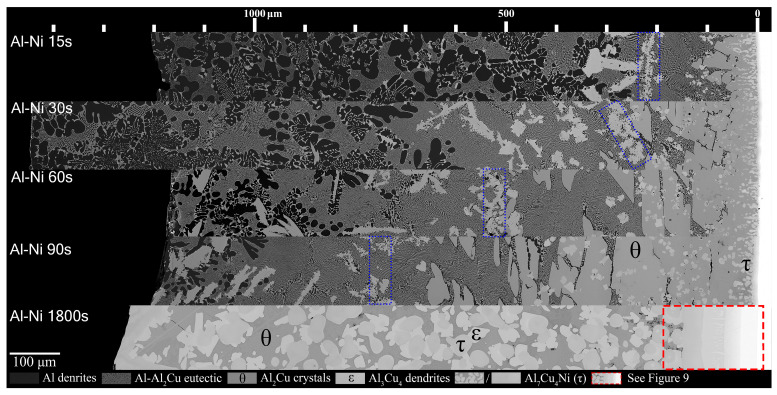
Mosaic SEM micrographs of Al-Ni/Cu couples ordered in increasing time above liquidus. θ, ϵ, and τ phase that form at longer diffusion times are labelled. The blue boxes highlight rough regions where τ crystals accumulate and mark the transition to primary θ dominated microstructures. The scale at the top starts at approximately the edge of the Cu phase in the substrate, which is roughly also the location of the S/L interface for Al-Ni 15–90s.

Figure 7 and Figure 8 show all the samples with respect to time above liquidus. Note, as mentioned previously, while each Al/Al-Ni substrate had a thickness of 1000 ± 50 μm initially, after melting, the L phase tended to form a spherical cap. This results in the maximum distance from the Cu substrate being higher than 1000 μm since the micrographs were taken from the thickest part of the solidified L phase. For the Al/Cu couples, there were several clear trends in the phases that formed. Al dendrites comprised a large area thickness of the Al 15s sample, between 800 and 1500 μm from the S/L interface. The volume fraction of Al dendrites significantly dropped in the Al 30s sample, and it only formed at a greater distance from the S/L interface. This trend continued in the Al 60s sample, until the Al 90s sample, which did not have any Al dendrites.

The eutectic Al-θ exclusive region noted in Figure 5A formed for most Al-Cu couples in Figure 7. This layer roughly had a thickness of 400 μm and initially formed around 420 μm from the S/L interface in the Al 15s sample. With increasing diffusion time, this region remained roughly 400 μm in thickness in the Al 30s and Al 60s samples; however, this region formed farther from the S/L in each proceeding sample. This region did not form in the Al 90s and Al 1800s samples. The hypereutectic layer in the Figure 5A schematic formed for all samples between 15 and 90 s. This layer was initially limited in size in the Al 15s sample, but grew with increasing diffusion time, until it dominated the entirety of Al 90s. The next layer, the continuous θ layer, initially had a small thickness in the Al 15s sample. This layer thickness grew with each sample’s increasing diffusion time. The Al 60s and Al 90s samples also contained needles of ϵ phase that had started solidifying isothermally while the reaction occurred in the furnace. ϵ cannot form upon solidification, and it is only stable above 580 °C (see Figure 1). These ϵ needles grew to large ϵ dendrites in the Al 1800s sample, with θ surrounding the dendrites. It is noted that at slower cooling rates (such as 4 °C/min), some of the ϵ phase can transform into the ζ phase via the peritectic reaction: L + ϵ → ζ [49], but the cooling rates in this work prevented significant occurrences of this reaction. As such, significant amounts of the ζ phase were not observed in the Al 1800s sample. The Cu boundary layer increased in thickness from <10 μm in the Al 15–60s samples, around 10–20 μm in the Al 90s sample, and to around 250 μm in the Al 1800s sample. This allowed it to be easily observed, which was the main purpose for the 1800 s samples.

Figure 8 shows all the Al-Ni/Cu couples. Al-Ni 15s had a large amount of primary Al dendrites, with some Al + Al_3_Ni_2_ eutectic structures surrounding them, all of which was encapsulated by eutectic Al–θ. While the ternary eutectic is noted as containing both θ and τ in phase diagrams of the Al-Cu-Ni system [44,61,62], when SEM images of the eutectic Al + θ + τ in the Al-Ni/Cu samples are compared to the binary Al + θ eutectic, the eutectics look indistinguishable. Research on the ternary system has revealed that the ternary eutectic reaction occurs at ~546 °C at roughly the composition Al-17.1Cu-0.6Ni [62]. This is very close to the binary eutectic composition of Al-17.3Cu at 547 °C. Furthermore, while the Ni solubility limit in Al is very low, it can be as high as 0.4% at elevated temperatures [34,63,64]. This, coupled with the uncertain maximum Ni solubility in the θ phase, leads to a eutectic microstructure in all the Al-Ni/Cu samples that have negligible volume fractions of τ. This is corroborated by the literature, where ternary eutectic Al-Cu-Ni alloys produced microstructures entirely of Al + θ [61,65].

It was noted that lower volume fractions of the Al dendrites, and the accompanying eutectic Al + Al_3_Ni_2_, formed with increasing time. This region also formed further away from the S/L interface as diffusion time increased. Unlike Al 90s, Al-Ni 90s was observed to contain small Al dendrites before the edge of the L phase. The previously stated eutectic morphology dominated region was observed in the Al-Ni 15s sample to be below the Al_3_(Cu, Ni)_2_ + τ ‘line’. This region grew in thickness as time increased. The Al_3_(Cu, Ni)_2_ + τ line also migrated with the eutectic dominated region, forming just above θ crystals. These ‘lines’ for each sample are marked by a blue dotted outline in Figure 8. This line became less pronounced as diffusion time increased with each sample. The Al_3_(Cu, Ni)_2_ and τ primary crystals found in the eutectic morphology dominated region were initially only found above the Al_3_(Cu, Ni)_2_ + τ line that formed in the Al-Ni 15s sample. In the Al-Ni 30s sample, these Ni-rich phases formed throughout the eutectic dominated region. In the Al-Ni 60s and Al-Ni 90s samples, these crystals were also observed forming in the Al dendrite dominated region. The total amount of these crystals increased from Al-Ni 15s to Al-Ni 30s, remained similarly frequent in the Al-Ni 60s sample, and decreased slightly in frequency as well as size in the Al-Ni 90s sample.

The hypereutectic layers found in the Al/Cu couples from Figure 7 are limited in thickness for the Al-Ni/Cu couples in Figure 8. This also is reflected in the thinner continuous θ layer. At the bottom of the continuous θ layer for Al-Ni/Cu samples, there were significant amounts of τ globules present. These τ particles were also found to grow from the Cu boundary layer. It is uncertain whether the majority of these τ globules formed during solidification or if it precipitated out of solution isothermally during the diffusion reaction at 670 °C. These globules were generally most frequent closest to the S/L interface. This τ globular crystal is differentiated from the τ phase that is observed to form on the surface of Al_3_(Cu, Ni)_2_ as the τ globules have smoother edges and do not contain any Al_3_(Cu, Ni)_2_ phase in its centre. This implies that the globules formed as primary τ, as seen at Cu concentrations above 11.9% in Figure 4A. Interestingly, the Al-Ni 90s sample had similar region boundaries between Al dendrite dominated, eutectic dominated, and θ dominated morphology to the region boundaries found in the Al 60s sample. Finally, for the Al-Ni 1800s sample, the L phase became fully saturated with Cu, thus θ precipitated throughout the entire sample. Large τ and ϵ crystals can be found in the microstructure, and given its sizes, these crystals could have only formed isothermally before cooling. Since there is no Al phase present in the entirety of this microstructure, the L phase must have had a Cu% > 30.4%, which is the limit after which no Al phase can form, seen in the phase diagram in Figure 4A. Since the initial Cu solubility limit is 25.8% for the Al-Ni/Cu couples, Ni must have become depleted due to τ precipitation, leading to an increase the Cu solubility limit. As there are also no small τ globules observed inside the θ phase in the Al-Ni 1800s sample, over the course of 1800 s, all the Ni in solution must have precipitated isothermally as τ crystals or dissolved into the Cu boundary layer.

#### 3.2.3. Phase Formation in the Cu Boundary Layer

**Figure 9:**
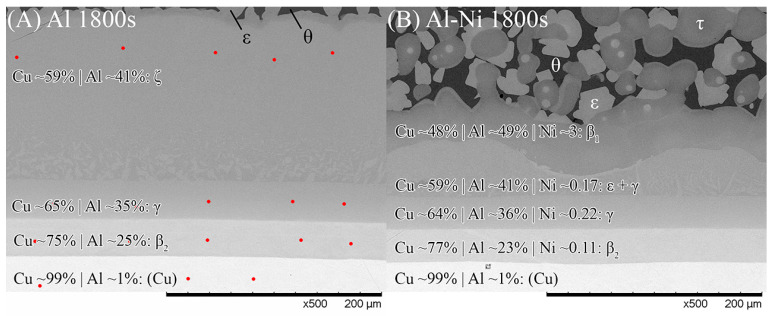
SEM images of the Cu boundary layer and its sublayers that formed from Al diffusion into solid Cu. Note this figure is not rotated and the Cu substrate is at the bottom. (**A**) Al 1800s, (**B**) Al-Ni 1800s. Red dots show locations where spot analysis was performed to calculate element composition. θ and ϵ phase, as well as the Al-Ni 1800s boundary, had spot analysis performed on higher magnification images or different locations (for easier measurements. All scan results found in Supplementary File S1). Averaged EDS results with predicted phases are shown for each layer.

It is important to study the Cu boundary layer as the effect of Ni on the diffusion of atoms through the S/L interface is reflected by the phases and their morphologies that form in this layer. Specifically, it is important to note the first phase that forms on the solid side of the S/L interface, as this can greatly affect diffusion through the layer. While the Cu boundary layer was too thin to easily identify the phases present for the 15–90 s samples, both 1800 s samples had a sufficiently thick Cu diffusion zone to allow EDS analysis of each sublayer. Figure 9 shows SEM images of the Cu diffusion zone from the Al and Al-Ni 1800s samples, with averaged EDS values for each sublayer. The sublayers, observed from the Cu substrate upwards, are Cu, β_2_, γ, and ζ, with a small layer of ϵ at the S/L interface (ϵ/θ once solidified) for the Al 1800s sample. The phases form in order from highest to lowest Cu% content and match the phases that intersect right to left along the isothermal 670 °C line shown in Figure 1. This result is also consistent with other experiments where liquid Al was reacted with solid Cu, and similar phases (depending on temperature) were reported in these works [50,54,66,67].

The Al-Ni 1800s sample had the same sublayers as Al 1800s from Cu to γ; however, instead of the ζ sublayer, it was replaced with a layer dubbed ϵ + γ. Figure 4B shows a single axis calculation from ThermoCalc. Cu concentration was varied, while the system was isothermally held at 670 °C, and the phases that formed are displayed. In this diagram, instead of the ζ phase forming like in the binary system, the corresponding region at 55% Cu is labelled as ϵ + γ instead. ThermoCalc predicted that Ni solubility caused the ζ phase to transform into ϵ + γ at ~626 °C. However, it must be noted that the overall Ni and Cu concentration values recorded for this region are very similar to the theoretical EDS values for the ζ phase, meaning this region could be either combination of phases from the measurements made. This work will use ϵ + γ as the label for this region as it agrees with ThermoCalc, but this region could be ζ due to the non-homogenous and non-equilibrium diffusion of Ni and Cu. Above the ϵ + γ sublayer, there was an additional sublayer identified as β_1_. This phase has a similar BCC crystal structure to the β_2_ phase, but different ratios of Cu and Ni incorporated into the sublattice [57,62,68]. β_1_ was shown to also form in Figure 4B. The CALPHAD calculation performed in Figure 4B controlled Ni content at 3% to exaggerate the volume fraction of β_1_ in the figure for clarity_._ Note that ThermoCalc labelled β_1_ as BCC_B2 and β_2_ as BCC_B2#2, which may cause some confusion. β_1_ is the only recorded equilibrium phase apart from θ (and Al) that contains a higher Al composition than Cu by atomic percent, at the temperatures and cooling rates present in this work, thus ruling out the possibility of the sublayer being η with increased Ni solubility. The β_1_ sublayer was covered by a layer of ϵ before the L phase, while some β_1_ was covered with τ that was then surrounded by ϵ before the L phase. Unlike the Al 1800s sample, the ϵ + γ, β_1_, and ϵ and τ sublayers in Al-Ni 1800s had variably shaped interfaces between them. This suggests that, unlike the diffusion between Cu, β_2_, and γ, the previously listed sublayers had diffusion that was more dominated by boundary diffusion with grain growth, rather than the volume diffusion or boundary diffusion without grain growth [54], that likely formed the lower sublayers of the Cu boundary layer. This is also supported by the observation of faint crystals of γ present in the ϵ + γ sublayer, as well as the thin grain boundaries observed in the β_1_ sublayer (Figure 9 and Supplementary File S1). The average thickness of the Al-Ni 1800s Cu diffusion zone was measured as ~150 μm. This is much lower than the ~250 μm thick Cu diffusion zone in the Al 1800s sample. Since, after 1800 s of isothermal conditions, any initially thermal gradients would have dissipated, this difference in layer thickness must be the result of Ni presence. Since interdiffusion between the solid phases present in the Al/Cu couple is much lower than interdiffusion between liquid and solid phases [12,66,69,70], the additional sublayer of β_1_ forming would lower the overall diffusion flux of Al into the Cu substrate, thus lowering the thickness of the Cu boundary layer. While β_2_ and γ were noted as being slightly thinner in the Al-Ni 1800s sample than in the Al 1800s sample, majority of this thickness reduction resulted due to the ϵ + γ sublayer being much thinner than the ζ sublayer. Overall, Ni addition reduced the diffusion of Al into the Cu substrate by introducing the β1 layer, which reduced the thickness of the Cu boundary layer. Since ϵ was the first phase that formed at the S/L interface for both the Al/Cu and Al-Ni/Cu couples, Cu diffusion into the L phase should have been be similar between the two couples.

#### 3.2.4. Effect of Ni on the “Continuous” θ Phase Layer

**Figure 10:**
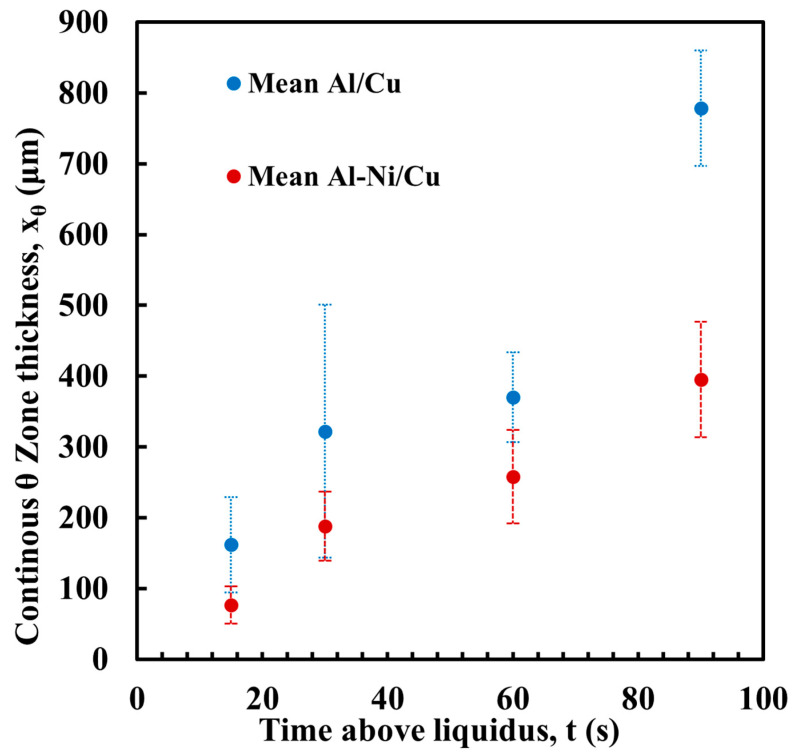
Average thickness of the continuous θ layer for each sample, with Al/Cu samples in blue and Al-Ni/Cu samples in red. Error bars represent one standard deviation for measured values.

**Figure 11:**
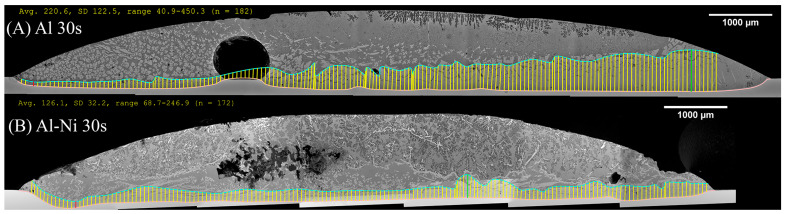
Composite SEM image of the entire diffusion couple for the (**A**) Al 30s and (**B**) Al-Ni 30s samples. The pink lines represent the boundary of the Cu substrate, while the blue line is the manually selected edge of the continuous θ layer (after smoothing via transformation into splines). The yellow lines are measurements made between the previously mentioned lines and represent the θ layer thickness. The units for the measurement summary (in yellow) are in pixels, with a conversion factor of 686 px: 1000 μm used. The given average values are used to plot Figure 10. Matching measurements for all other samples are found in the Supplementary Figure S3.

Figure 10 shows the measurement of the average thickness of the continuous θ layer. This was measured as a reduction in the volume of this phase, which was a key aim of this work. Select images of the measurement are shown in Figure 11, with the rest provided in the Supplementary Figures S2. Note, the large pore/defects present in the previously stated figures accounted for only a small volume fraction of the sample. Since they are likely gas defects, their formation would be during solidification, which is after Cu diffusion in the liquid has already taken place, thus their negligible effect on the microstructures near these defects. The measurement shows that the average layer thickness of the continuous θ layer was consistently lower in the Al-Ni/Cu couples. The Al/Cu couples had θ layer thicknesses around 1.5–2 times that of those found in the Al-Ni/Cu couples. There was one sample that was an outlier. Al 30s measured a continuous θ layer thickness of 322 μm with a larger standard deviation of 179 μm. The other samples had standard deviations between 29 and 82 μm. The entire composite micrograph of the Al 30s couple is shown in Figure 11. This image shows that the continuous θ growth in this sample was quite uneven, with the left side having a much smaller continuous θ layer, and the right having a very thick zone. This was likely caused due to disturbances and tilting of the sample during its removal from the furnace, with this human error causing the lopsided growth of the θ phase. Figure 11 also shows the Al-Ni 30s sample, which, comparatively, was more uniform with its θ layer thickness. This is reflected in its lower standard deviation value of 49 μm compared to Al 30s. The mosaic SEM images in Figure 11 also highlight the previously mentioned curvature of the L phase and its formation of a spherical cap shape upon melting. The furthest horizontal edges of all samples were observed to generally have microstructures that result from higher Cu concentrations in the L phase.

### 3.3. Modelling of Cu Concentration in the Liquid Phase Using Fick’s Laws

To validate the reported data in the previous sections, a simulation for Cu concentration within each sample was produced using Fick’s laws of diffusion. This model was based off previous research investigating the diffusion behaviour between solid Cu and liquid Al [52,53,54]. All modelling in this work was performed using these assumptions:

1. Only diffusion in the one-dimensional case is considered. The S/L interface is the point that marks the start of diffusion in the model, and the edge of the L phase marks the end, thus making it a finite diffusion case. For both types of couples, ϵ is treated as the solid phase from which Cu diffuses into the L phase, and its composition is fixed at 55% Cu.
2. Diffusion coefficient for Cu in liquid Al (*D*) is fixed at 670 °C is 5 × 10^3^ μm^2^/s [71] and does not vary with local Cu or Ni concentration or temperature variation.
3. The maximum Cu concentration of the L phase at 670 °C was found from the equilibrium phase diagrams in Figure 1 and Figure 3. This value was fixed for all time steps at 37% for Al/Cu couples and 25.8% for Al-Ni/Cu couples. When Cu starts to diffuse into the L phase, the L phase at the S/L interface steps up instantly to the solubility limit for the sample. All other diffusion after the initial location follows Fick’s laws.
4. Only diffusion in the time range of 0–90 s will be modelled. After 90 s, significant non-linear events, such as solid crystal precipitation, non-power law crystal growth, etc., took place, which are not considered.
5. Similarly, Al and Ni diffusion, both into the Cu boundary layer and interdiffusion in the liquid, are treated as negligible for the time frames present in the model. This is based off the fact that diffusion coefficients for interdiffusion of Al, Al diffusion between solid Al-Cu phases, and Ni diffusion in liquid Al and solid Cu are at maximum only ~20% of the Cu diffusion coefficient in liquid Al at 670 °C [51,52,53,54,66,72,73].
6. Diffusion after the set diffusion time, such as during the sample’s removal or solidification, is not considered. Solute rejection and other solidification mechanisms are considered when analysing local microstructures after solidification.
7. The spherical cap shape of the L phase [74] is not considered. Maximum length for diffusion will be 1000 μm + migration of the S/L interface.

To set up the diffusion model, Fick’s second law of diffusion was used, and it is shown in Equation (1) below, where *C* represents the concentration of Cu within the liquid in at.%, *D* represents the diffusion coefficient of Cu solute in liquid Al (which as stated in assumption 2 is to be 5 × 10^3^ μm^2^/s), *x* represents the distance from the S/L interface in μm, and *t* represents time (s):

(1) $$\frac{\partial C}{\partial t}=D\frac{\partial^{2}C}{\partial x^{2}}$$

The partial derivative of the diffusion equation can be numerically approximated using the explicit method Forward Time-Centred Space scheme (FTCS) [75], where Equation (2) describes Fick’s second law as the finite difference:

(2) $$\frac{C_{i}^{n+1}-C_{i}^{n}}{∆t}=D(\frac{C_{i-1}^{n}-2C_{i}^{n}+C_{i+1}^{n}}{∆x^{2}})\;C_{i}^{n+1}=C_{i}^{n}+\frac{D∆t}{∆x^{2}}(C_{i-1}^{n}-2C_{i}^{n}+C_{i+1}^{n})$$

Equation (2) allows the computation of the concentration value at a specific location in the next time step, given the concentration values of neighbouring locations at the current time step. The values *n* and *i* represent the numerical value of the current time step and number of the position step, respectively. Δ*t* and Δ*x* are the discrete sizes of the time and distance steps, respectively. Δ*x* was chosen to be 1 μm while Δ*t* was set such that the stability condition for FTCS (labelled *σ*) is met. Setting *σ* = 0.5 gives Δ*t* = 1 × 10^−4^ s. The stability condition for FTCS is shown in Equation (3):

(3) $$\sigma =\frac{D∆t}{∆x^{2}}\leq 0.5$$

**Figure 12:**
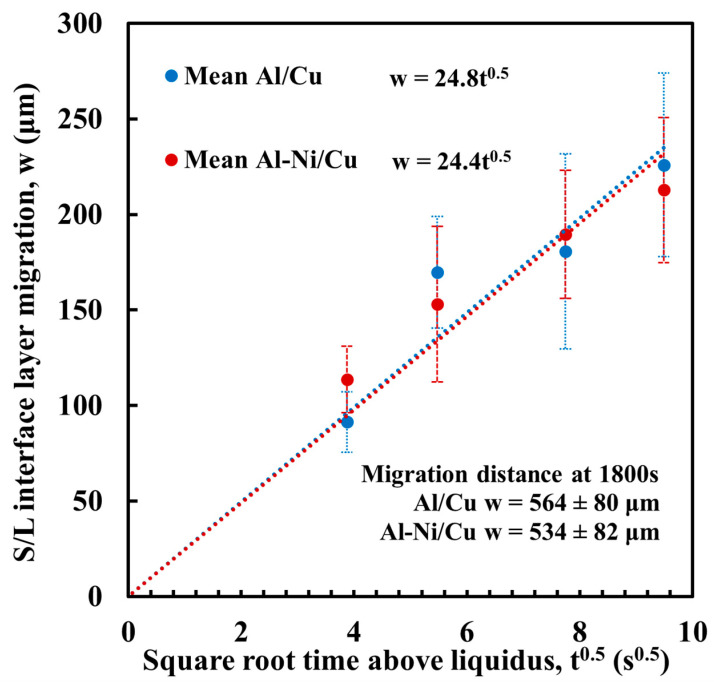
Measured migration of the S/L interface *w* plotted against square root time. Equation for each trend line is given next the legend. Migration distance for 1800 s samples are stated but not included in calculation for trendline, as at larger time intervals the relationship between *w* and *t* no longer follows a power law [53].

Since the L phase is not semi-infinite, assignment of a maximum length between the edge of the L phase and the S/L was needed. This value could vary depending on several variables. One of these variables was described by the modelling work of Tanaka and Kajihara [52,53,54], where it is stated that the S/L interface between the liquid Al and solid Cu slowly migrates away from its initial location in the opposite direction to Cu diffusion according to the formula:

(4) $$w=Kt^{0.5}$$

where *w* is the migration distance of the S/L interface (μm), *t* is diffusion time (s), and *K* is a constant (μm/s^0.5^) that is based on the diffusion coefficient *D* and Cu concentrations present in the phases either side of the S/L interface. This *K* value was calculated for the samples in this work via measurement of the migration distance. This was performed using the same method to measure continuous θ layer thickness in Figure 11, but, instead, measuring the distance between the S/L interface and a horizontal line drawn joining the exposed unreacted surface of the Cu substrate on either side of the sample (measurements in Supplementary Figure S3). Figure 12 shows the results, plotting the square root of *t* against *w*. The trendlines for both the Al/Cu and Al-Ni/Cu couples are shown on the graph. The average value of *K* was 24.6 μm/s^0.5^. This measured value is slightly higher compared to the *K* value of 20.27 μm/s^0.5^ at 973 K reported by Tanaka et al. [54]; however, this difference likely results from the different experimental conditions, as this work had a slightly curved S/L interface due to the shape of the L phase, as well as the small amounts of diffusion that would have occurred between the partially melted Al or Al-Ni liquid and the Cu substrate. The FTCS model will assume there is no diffusion before the Al or Al-Ni has completely melted and will only be modelling the centre of the samples’ S/L interfaces, which was seen to be relatively flat. Substituting for *K* into (4) gives

(5) $$w=24.6\sqrt{t}$$

Regarding assumption 7, to simplify the modelling, it was assumed that the droplet can be flattened such that the edge of the L phase starts at 1000 μm from the S/L interface, thus the L phase will be assumed to not change shape or size from the initial 10 × 8 × 1 mm dimension of the solid metal. As a result, total diffusion distance will be modelled by the formula:

(6) $$x_{max}(\mu m)=(1000+w)=\left(1000+24.6\sqrt{t}\right)$$

To run the FTCS model, the initial and boundary conditions needed to be set up. For all samples, the Neumann boundary condition [76] was set at *x_max_*. This states that since Cu cannot diffuse past the edge of the L phase, the local diffusion flux must = 0:

(7) $$\frac{\delta C_{max}^{n}}{\delta x}=0$$

This ensures that Cu slowly accumulates at the furthest extent of the L phase and the total amount of Cu in the L phase increases with time. At the end of the calculation for each time step, this boundary condition was achieved by letting

(8) $$C_{i_{max}}^{n}=C_{i_{max}-1}^{n}$$

To model *x_max_* at different times, firstly, the maximum time value of 90 s was substituted into Equation (6), giving *x_max_* = 1233 μm. Δ*x* was fixed to 1 μm, giving a total of *i* = 1234 distance steps (including the 0th step). To vary *x_max_* with t, *C_i_*^0^ was set to 37% (or 25.8% for the Al-Ni couples as stated in assumption 3 for Al/Cu couples) for all *i* ≤ 233. This set the initial distance of the S/L interface at *x_max_* − 233 μm = 1000 μm. At the end of every time step, *C_i_* was reset up to the maximum Cu solubility for all *i* < *i_w_*. Equation (9) allows for *i_w_* to be modelled and accounted for whenever the S/L migrated 1 μm using the floor function (rounding down to the nearest integer). *i_w_* is given by

(9) $$i_{w}=233-\left\lfloor w\right\rfloor =233-\left\lfloor 24.6\sqrt{t}\right\rfloor$$

Setting all initial values conditions:

(10) $$C_{i}^{0}=0\;if\;i>233\;\mathrm{and}\;C_{i}^{0}=Max\;Cu\;solubilty,\;if\;i<233$$

All boundary conditions reapplied each time step include Equation (8) as well as:

(11) $$C_{i}^{n}=Max\;Cu\;solubilty\;if\;i<i_{w}$$

**Figure 13:**
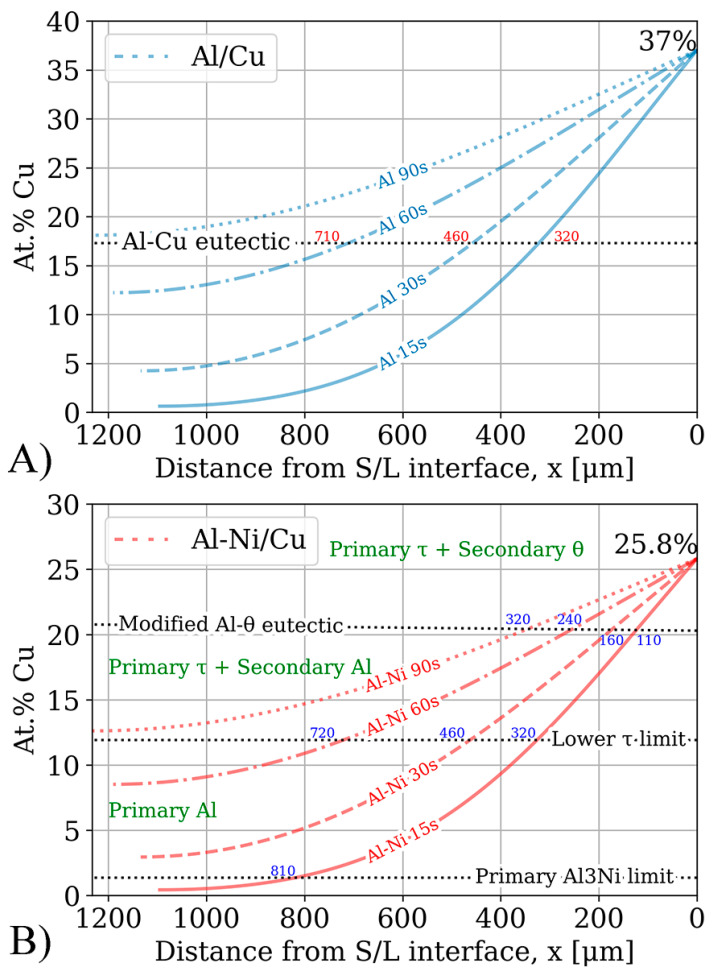
Result of the simulation based on Fick’s laws. The *x*-axis plots the distance from the S/L interface in μm, while the *y*-axis shows the local Cu concentration in at.%. The *x*-axis has been inverted such that the distance values are parallel to the scale bars shown in Figure 7 and Figure 8. Notable compositional boundaries from Figure 1, Figure 3 and Figure 4 are also demarcated. (**A**) Cu concentration profiles for the Al/Cu diffusion couples. *x*-values to the nearest 10 μm for intersection with the Al-Cu eutectic line shown in blue. (**B**) Cu concentration profiles for the Al-Ni/Cu diffusion couples. *x*-values to the nearest 10 μm for intersections with relevant primary phase limits in the pseudo-binary system (Figure 4) shown in blue. Primary and some secondary phases shown in green.

The results of running the FTCS scheme for 15–90 s is plotted in Figure 13. The figure plots the distance from the S/L interface in μm on the *x*-axis and local Cu concentration in at.%. Important composition limits were drawn for the Al/Cu and Al-Ni/Cu couples. The intersection between the concentration profile and the Al-Cu eutectic points in the binary and ternary system are also shown in each sub-figure.

### 3.4. Discussion of Solidification in the Diffusion Couples and Comparison with FTCS Scheme

#### 3.4.1. Comparison of Microstructure Evolution vs. Simulated Cu Concentration Evolution

The Al-Cu eutectic composition, plotted as the dotted black line in Figure 13A, acts as a boundary marker for possible microstructures upon solidifying. At Cu concentrations lower than this value, the expected microstructure upon solidification should be hypoeutectic. At Cu concentrations above this value, hypereutectic or entirely θ based microstructures are expected upon solidification. Since the *x*-axis measured the distance from the S/L interface (which moved with time) to L, the tail of the concentration profile ended at varying *x*-values.

The Cu concentration profiles from Figure 13 are compared to the SEM images to see if the various observed microstructural morphology layers validated the FTCS model. Most of the samples matched well with the predicted microstructures from the FTCS Cu concentration profiles. For the Al 15s and Al 60s samples, the average thickness of the continuous θ layer was found to be roughly half the distance between the corresponding FTCS concentration curve and the intersection with Al-Cu eutectic composition line (shown as the values next to each line in Figure 13A). It is presumed that this pattern would also hold if Al 30s did not have the previously discussed lopsided θ layer thickness. The Al 90s Cu concentration curve does not cross the Al-Cu eutectic line at any location below *x_max_*. This implied that no region of the sample would have a hypoeutectic or eutectic microstructure upon solidification. This prediction from the FTCS model is validated, as Figure 7 showed that the Al 90s sample had primary θ crystals forming throughout the entirety of the sample, characteristic of hypereutectic liquid compositions. One difference between the Al/Cu couples in Figure 7 and the Cu concentration curves in Figure 13 is that while Fick’s laws predict a smooth Cu concentration profile, the samples have distinct regions which are entirely hypoeutectic, eutectic, hypereutectic, and the continuous θ layer, and these regions often transition abruptly. This can be explained by solute segregation during the non-homogenous solidification of the sample, leading to solute concentration in the regions of the samples that solidify last. These regions would generally be closest to the eutectic lines in Figure 13. This would lead to the tiered layering found in Al/Cu couples. This solute segregation upon solidification could have allowed regions with a local Cu concentration above 33% to enrich the yet-to-solidify L phase with additional Cu atoms. This would have caused these regions with <33% Cu to increase in Cu content, thus solidifying entirely as θ with no/fewer eutectic morphologies. This would have extended the continuous θ layer beyond its theoretical limits of >33% local Cu concentration. The observed microstructure shows that the continuous θ layer extends till around 25–28% Cu, supporting the solute rejection hypothesis. The presence of the ϵ phase is one limitation of the FTCS model. Since this phase precipitates out of solution isothermally during diffusion, its presence implies that the L phase was locally supersaturated with Cu. Fick’s first law defines the diffusion flux J (μm/s) as

(12) $$J=-D\frac{\delta C}{\delta x}$$

If the diffusion flux of Cu diffusing into the L phase from ϵ is greater than the diffusion flux of Cu diffusing away from the S/L interface, then the local Cu concentration would increase above 37%, thus causing the solidification of ϵ phase. Since small amounts of ϵ solidified in the Al 60s sample, treating it as the starting value for ϵ precipitation, *J* was calculated at the S/L interface. This was performed by taking the gradient of the Al 60s Cu concentration profile (*δC*/*δx* = mole fraction/μm) and multiplying by *D*, giving *J* = 1.11 × 10^3^ μm/s. Therefore, the flux value of Cu diffusion from ϵ into the L phase must be some value higher than *J* = 1.11 × 10^3^ μm/s at 670 °C and 60 s. Overall, modelling Fick’s second law of diffusion using FTCS was found to produce accurate predictions of the real microstructures found in the Al/Cu couples, and, thus, this methodology could also apply to the Al-Ni/Cu couples.

While the local Cu concentrations of Al/Cu couples in Figure 13A can easily be compared to the binary Al/Cu couples in Figure 7 to predict the local microstructure upon solidification, prediction of microstructure upon solidification in the ternary Al-Cu-Ni system is more challenging. Figure 13B has some crucial compositions plotted as dotted black lines. These lines highlight the limits of compositions for a particular primary phase during solidification. Generally, primary phases tend to form noticeably larger dendrites or crystals relative to the surrounding phases. This feature of the microstructure can be used to analyse and compare results in Figure 13B to microstructures in Figure 8. One of these lines, marking the limits of primary phases in Figure 13B, is the limit of primary Al_3_Ni crystals. This limit is calculated from the pseudo-binary phase diagram in Figure 4A. It shows that primary Al_3_Ni can only solidify below Cu concentrations of 1.36%. FTCS modelling predicts that Al-Ni 15s should be the only sample where primary Al_3_Ni crystals could form. This prediction is corroborated by SEM observations, as seen in Figure 5H, and Al-Ni 15s was indeed the only sample where primary Al_3_Ni was observed. While Figure 13B notes that the limit of primary Al_3_Ni formation should be ~810 μm from the S/L interface, some Al_3_Ni crystals are noted at ~650 μm in Figure 8. This discrepancy likely results from Figure 8 showing the thickest section of the L phase, thus the total diffusion length for the imaged microstructure is higher than *x_max_* in Equation (6). This would mean that Cu atoms would have to diffuse for a longer total distance, and it would take slightly longer the Cu concentration in the sample to match the profile predicted by FTCS. Nevertheless, the difference between the 810 and 650 μm for the predicted and observed limits of primary Al_3_Ni crystals formation is quite reasonable, especially given the many simplifications/assumptions made while constructing the FTCS model and the non-equilibrium solidification of the sample.

The next primary phase composition limits were for primary Al dendrites. From Figure 4, primary Al dendrites form between Cu concentrations above the upper limit for Al_3_Ni and the lower limit for primary τ formation. These limits were 1.36–11.9% Cu respectively. For the Al-Ni 15s and Al-Ni 60s samples, the limit on primary Al solidification was roughly 320 and 720 μm, respectively. Large Al dendrites were indeed noted as only forming at distances above the stated values for the respective samples in Figure 8. For the Al-Ni 30s sample, the Al dendrite limit is calculated as roughly 460 μm. While the limited snapshot seen in Figure 8 suggests that the Al dendrite limit is ~600 μm, observation of the general microstructure in the rest of the sample roughly corroborates the 460 μm limit (see Figure 11 or Supplementary Figures S1). Overall, the predicted limits of primary Al dendrite formation from combining CALPHAD in Figure 4 and the diffusion curves in Figure 13 are corroborated by microstructural observations. The above results support the accuracy of diffusion curves produced for Al-Ni couples by FTCS at lower Cu concentrations.

**Figure 14:**
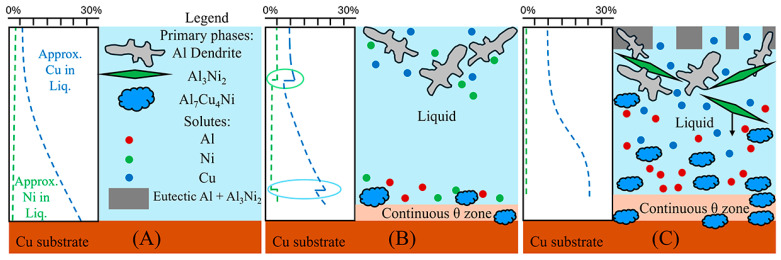
Schematic visualising discussion and explanation for presence and distribution of large primary Al_3_(Cu, Ni)_2_ and τ crystals in the Al-Ni 30s sample. (**A**) The initial sample with liquid in contact with the Cu substrate with varying Cu concentration according to Figure 13. (**B**) This shows the initial solidification where primary Al dendrites form on the top of the liquid, while the continuous θ layer forms at the bottom of the liquid. The Al dendrites reject Cu and Ni solutes into the liquid, while the continuous θ layer rejects Ni and Al into the liquid. This causes spikes in the local solute concentration shown in the ovals. (**C**) The spikes in Ni concentration causes the nucleation of Al_3_(Cu, Ni)_2_ near the Al dendrites and τ near the continuous θ layer. Some of the Al_3_(Cu, Ni)_2_ crystals drop in the liquid due to their relative high density. The concentration curve for Cu slowly homogenises from diffusion and solute rejection. τ is also noted as concentrating.

The next region of interest from Figure 13B is the region with primary τ solidification, with secondary Al formation. In the Al-Ni/Cu samples, however, this region did not have a consistent microstructure. Al-Ni 90s in Figure 8, at distance values above 1000 μm, had smaller Al dendrites (compared to other samples) surrounded by large crystals of Al_3_(Cu, Ni)_2_ + τ. This microstructure is hypothesised to form in the primary τ + secondary Al region of the pseudo-binary system if the solidification occurred without solute segregation. Since this region is observed to have the lowest liquidus temperature from Figure 3 and Figure 4, this region is likely receiving a large amount of rejected solute from other regions of the L phase. One consequence of this is the presence of large, likely primary Al_3_(Cu, Ni)_2_ crystals. As seen in Figure 4A, Al_3_(Cu, Ni)_2_ should not form as a primary phase. It either forms from a peritectic transformation of eutectic Al + Al_3_Ni into Al + Al_3_(Cu, Ni)_2_ or solidifying from the L phase as the ‘fishbone’ eutectic previously discussed. This type of solidification can only occur in the region highlighted in red in Figure 4A. Neither the peritectic reaction nor direct solidification from liquid should nucleate the large Al_3_(Cu, Ni)_2_ observed. Looking at Figure 3, the liquidus projection of the Al-Cu-Ni system below 670 °C reveals that the local Ni concentration would have to be greater than 3.6% and Cu concentrations would need to be between ~7–15%. These conditions could possibly occur during the highly non-equilibrium and non-homogenous solidification found in the Al-Ni/Cu couples. Solute from the primary Al dendrites would enrich the central liquid with Ni and Cu. This could then trigger the formation of primary Al_3_(Cu, Ni)_2_ crystals. These Al_3_(Cu, Ni)_2_ crystals would then grow and influence the local microstructure. Since Al_3_(Cu, Ni)_2_ and τ are Cu- and Ni-rich phases compared to other phases that form in the liquid, their respective crystals would also start sinking in the liquid. If this were to occur, these phases would collect on the solidification front that is growing from the bottom of the sample. This would explain the observed ‘line’ formations highlighted by the blue dotted boxes in Figure 8, as these are consistent lines/formations of Al_3_(Cu, Ni)_2_ + τ crystals. A schematic of this process is shown in Figure 14, to help visualise the proposed solidification behaviour that could lead to the observed microstructures from Figure 8. Since Al-Ni 90s is predicted to not have primary Al dendrites to drive this solute segregation triggered solidification, it would explain the sample’s lower observed volume fraction of Al_3_(Cu, Ni)_2_ and τ, as well as the primary τ + secondary Al noted in the sample. Generally, as time increases, less Al_3_(Cu, Ni)_2_ and more τ is expected to form, according to Figure 4A and Figure 13B. This matches the observation from Figure 8 made in the previous discussion sections.

The final compositional limit of note was the Al-θ eutectic point in the pseudo-binary system. This was found to be around 20.8% Cu. This value is 3.3% higher than the binary eutectic point at 17.5% in the Al-Cu system. However, around this value, τ was the predicted phase to form as the primary phase, unlike Al or θ in the binary system. It was hypothesised that below this Cu concentration, no large θ crystals would form. While this hypothesis held for lower diffusion times, larger crystals of θ were observed to form in Al-Ni 60s and Al-Ni 90s samples. This is once again likely due to solute rejection from the θ phase during solidification. Generally, the x-intercepts related to the Al-θ eutectic line in Figure 13B correlated with the thicknesses of the continuous θ layer in Figure 10.

#### 3.4.2. Limitations of the FTCS Model

The FTCS model of Fick’s laws was able to relatively accurately predict and match the observed microstructures in all Al/Cu couples and the low Cu concentration regions in the Al-Ni/Cu couples. Thus, the varied Cu concentration profile within the diffusion couples and their solidification behaviour could reasonably explain the reason for the varied microstructures between different solidification times and between the Al/Cu and Al-Ni/Cu couples. Given this, there are still some limitations with the model that require discussion. The main limitations are summarised as such:

1. Assumption 1 limiting diffusion to 1D neglects the reality of 3D diffusion within the L phase due to its complex spherical cap shape, as well as neglecting the effect of the curved S/L interface.
2. Reported values for *D* in the literature (at 700 °C) range from approximately 4 to 6 × 10^3^ μm^2^/s [48,54,71]. *D* can also vary based on the local liquid’s Cu concentration [71]. An alternative version of Figure 13, assuming D = 4 and 6 × 10^3^ μm^2^/s, can be found in the Supplementary Figure S4. The effect Ni solubility on *D* is also unknown and unreported in the literature to the best of the authors’ knowledge. While the median value of 5 × 10^3^ μm^2^/s was chosen for this work, results are very sensitive to the value of *D*.
3. Since the Al-Ni 1800s sample only contained precipitated τ and ϵ, and solidified entirely as θ upon cooling, the L phase must have been completely depleted of Ni. This implies that Ni concentration in the L phase decreased with time. This would then lead to an increase in the maximum solubility of Cu in the L phase, as seen in Figure 3. This would affect assumption 3 and the FTCS model. Since it is not clear if the τ observed near the S/L interface nucleated upon cooling or isothermally during the diffusion reaction, like the ϵ in the Al/Cu couples, the rate of the Ni depletion is unknown. While it is assumed that solid precipitates do not affect the result significantly, this assumption may be false. Ni diffusion into the β_1_ sublayer could also have a similar effect.
4. FTCS itself is mathematically simplified and it is an explicit solution to the partial differential equation of Fick’s second law. More mathematically rigorous or implicit solutions would result in more accurate results.

Despite the stated limitations, the Cu concentration profiles from the FTCS solution to Fick’s second law is concluded to reasonably explain observed microstructures in most samples. The results of this modelling, used in conjunction with CALPHAD methods, could accurately predict the observed microstructure while providing possible explanations for unusual microstructural features such as the large Al_3_(Cu, Ni)_2_ crystals observed. The differences between the Al/Cu and Al-Ni/Cu couples could satisfactorily be explained by how Ni modifies the binary Al-Cu system. This modification by Ni also led to lower volume fractions of θ forming due to Ni limiting the maximum solubility of Cu in the L phase, slowing Cu diffusion into the L phase. It also increased the eutectic Al + θ composition location by 3.3% to 20.8% Cu. This finding would likely mean that Ni presence in Al-based braze fillers would limit how much Cu initially diffuses into liquid braze filler, thus producing less of the brittle θ phase upon solidification. However, now that diffusional behaviour and microstructural modification due to Ni have been demonstrated, analysing the complicated effects of these findings on dissimilar brazed Al to Cu joints is required to further evaluate the feasibility of using Al-Ni alloys as braze fillers.

## 4. Conclusions

This study investigated the differences between the diffusion couple liquid Al vs. liquid Al-3at.% Ni and a solid Cu substrate at 670 °C. The evolution of the microstructures in each couple over a range of reaction times (15, 30, 60, 90, and 1800 s) was observed. The findings are summarised as

1. The reaction between liquid Al and the Cu substrate formed four distinct microstructural layers upon solidification. These layers were characterised by their morphologies: primary Al dendrites, eutectic dominated morphology, primary Al_2_Cu (θ) dendrites, and the continuous *θ* layer, where θ composed > ~90% of the volume fraction.
2. The solid/liquid (S/L) interface marked the transition from the liquid to the solid Cu substrate. There were several unique solid phases that formed on the surface of the Cu substrate This diffusion layer consisted of (in descending order of Cu content) FCC Cu(Al), Cu_3_Al (β_2_), Al_5_Cu_8_ (γ), Al_9_Cu_11_ (ζ), and HT_AlCu (ϵ). ϵ also formed as a solid precipitate in the liquid after 60 s of diffusion had elapsed.
3. The reaction between liquid Al-Ni and the Cu substrate formed similar regions to those present in the Al/Cu couples. Additional phases of Al + Al_3_(Cu, Ni)_2_, Al_7_Cu_4_Ni (τ), and primary Al_3_(Cu, Ni)_2_ were noted to be present. The Cu diffusion zone contained the sublayers: FCC Cu(Al), Cu_3_Al (β_2_), Al_5_Cu_8_ (γ), Al_5_Cu_8_ (γ) + HT_AlCu (ϵ), Al_0.49_Cu_0.48_Ni_0.03_ (β_1_), and HT_AlCu (ϵ) + Al_7_Cu_4_Ni (τ).
4. The presence of Ni reduced the formation of the continuous Al_2_Cu (*θ)* layer. All Al-Ni/Cu couples had a reduction of 30–50% in the thickness of this layer (at equivalent diffusion times) when compared to Al/Cu couples. This result was attributed to the Ni presence in the liquid phase lowering the maximum liquid solubility of Cu to ~25.8%, which lowers the Cu concentration gradient in the sample, requiring more time for Cu to diffuse in the liquid and concentrate enough to form θ. Ni also modifies the Al-Cu system, such that large θ crystals can only solidify above Cu concentrations of 20.8%, instead of 17.5% in the binary system.
5. The Forward Time-Centred Space Scheme (FTCS) for solving Fick’s second law of diffusion was found to viably model Cu diffusion in all the couples. Its results for Cu concentration at various distances from the S/L interface, when combined with CALPHAD, generally correlated with observed microstructures at the same distances from the S/L in corresponding diffusion couples.

## Figures and Tables

**Table 1:** Summary of all the phases that could possibly form in the samples based off Figure 1 and Figure 3. Note, HT = high temperature, VA = site vacancy (used in ThermoCalc documentation).

ThermoCalc Name (TCBIN/TCAL7)	Phase Stoichiometry/Name	Formula Unit of Lattice	Lattice Composition (at.%)		
FCC_A1	Al or Cu	Al or Cu	-		
AL2CU_C16	Al_2_Cu θ	(Al)2(Cu, Ni)1	Al	~67%	
			Cu	~33%	
			Ni	0–0.132%	
ALCU_ETA	AlCu η	(Al, Cu)1(Cu)1	Al	~50%	
			Cu	~50%	
D81_ALCU/ALCU_EPS	HT_AlCu ϵ	(Al, Cu)1(Cu)1	Al	Bal	
			Cu	~54–57%	
			Ni	<2%	
BCC_B2/BCC_B2#2	HT_BCC β	(Al, Cu)1(VA)3		β_1_	β_2_
			Al	~50–46%	22–25%
			Cu	~49–38.5%	75–78%
			Ni	Bal	~0–3%
D83_ALCU/GAMMA_D83	Al_4_Cu_9_ γ	(Al, Ni)4(Al, Cu, Ni)1 (Cu, Ni)8	Al	~26–32%	
			Cu	~68–74%	
			Ni	<2%	
ALCU_DELTA/ALCU_DEL	Al_2_Cu_3_ δ	(Al)2(Cu)3	Al	40%	
			Cu	60%	
ALCU_ZETA	Al_9_Cu_11_ ζ	(Al)9(Cu)11	Al	45%	
			Cu	55%	
AL3NI_D011	Al_3_Ni	(Al)3(Ni)1	Al	75%	
			Ni	25%	
AL7CU4NI	Al_7_Cu_4_Ni τ	(Al)1(Cu, Ni, VA)1	Al	~58.4%	
			Cu	~33.3%	
			Ni	~8.3%	
AL3NI2	Al_3_Ni_2_	(Al)3(Al, Cu, Ni)2(VA, Ni)1	Al	Bal	
			Cu	15.4–~23%	
			Ni	14–40%	

## Data Availability

The original contributions presented in this study are included in the article/Supplementary Material. Further inquiries can be directed to the corresponding author.

## Supplementary Materials

The following supporting information can be downloaded at: https://www.mdpi.com/article/10.3390/ma18245689/s1. Figure S1: ZIP file with images showing the macroscopic cross-section of all samples present in this study; Figure S2: ZIP file with measurements of Figure S1 of the distance of the “continuous” Al_2_Cu layer. Measurements stated in pixel units. Conversion factor is 686 px: 1000 μm; Figure S3: ZIP file with measurements of Figure S1 measuring the S/L interface migration distance for each sample. Measurements made in pixel units. Conversion factor is 686 px: 1000 μm; Figure S4: ZIP file with alternate versions of Figure 13 assuming the diffusion coefficient = 4 or 6 × 10^3^ μm/s; File S1: docx files with EDS measurements point measurements for phases present in the Cu diffusion zone; Code S1: txt file with ImageJ macro code for analysing distance between two lines in the software. Code S2: Python code produced for FTCS modelling; Code S2: Python code for producing Figure 13A; Code S3: Python code for producing Figure 13B.

## Abbreviations

The following abbreviations are used in this manuscript:

SEM	Scanning electron microscope
EDS	Energy dispersive spectroscopy
CALPHAD	Computer Coupling of Phase Diagrams and Thermochemistry
IACS	International Annealed Copper Standard
TIG	Tungsten inert gas
HVAC	Heating, ventilation, and air conditioning
S/L	Solid/liquid
L phase	Liquid phase
FTCS	Forward Time-Centred Scheme
θ,ϵ,τ, etc.	Phases present in the Al-Cu-Ni ternary system. See Table 1.
