# Supplementary material for: Effect of Ni Addition on the Solidification of Liquid Al and Solid Cu Diffusion Couples
Source: Materials (Basel). 2025 Dec 18;18(24):5689. doi: 10.3390/ma18245689 (PMC12735080; doi:10.3390/ma18245689)
Supplement: Supplementary file 1 [file materials-18-05689-s001.zip › Supplementary Figures/Files S1/Al 1800s diffusion zone/EDS scans 1.pdf]

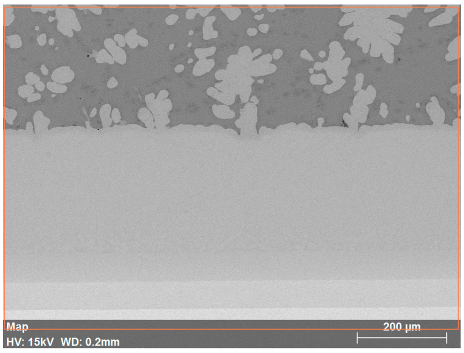

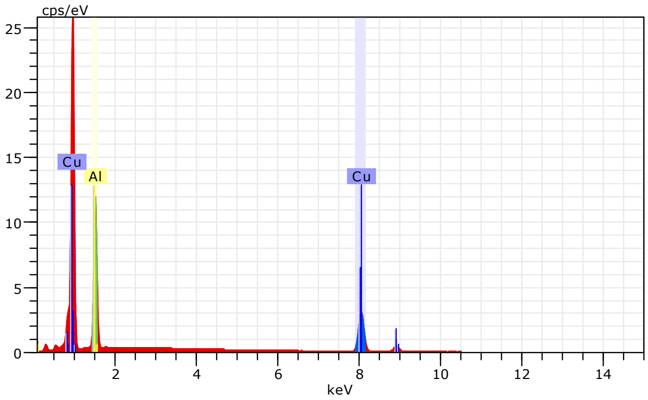

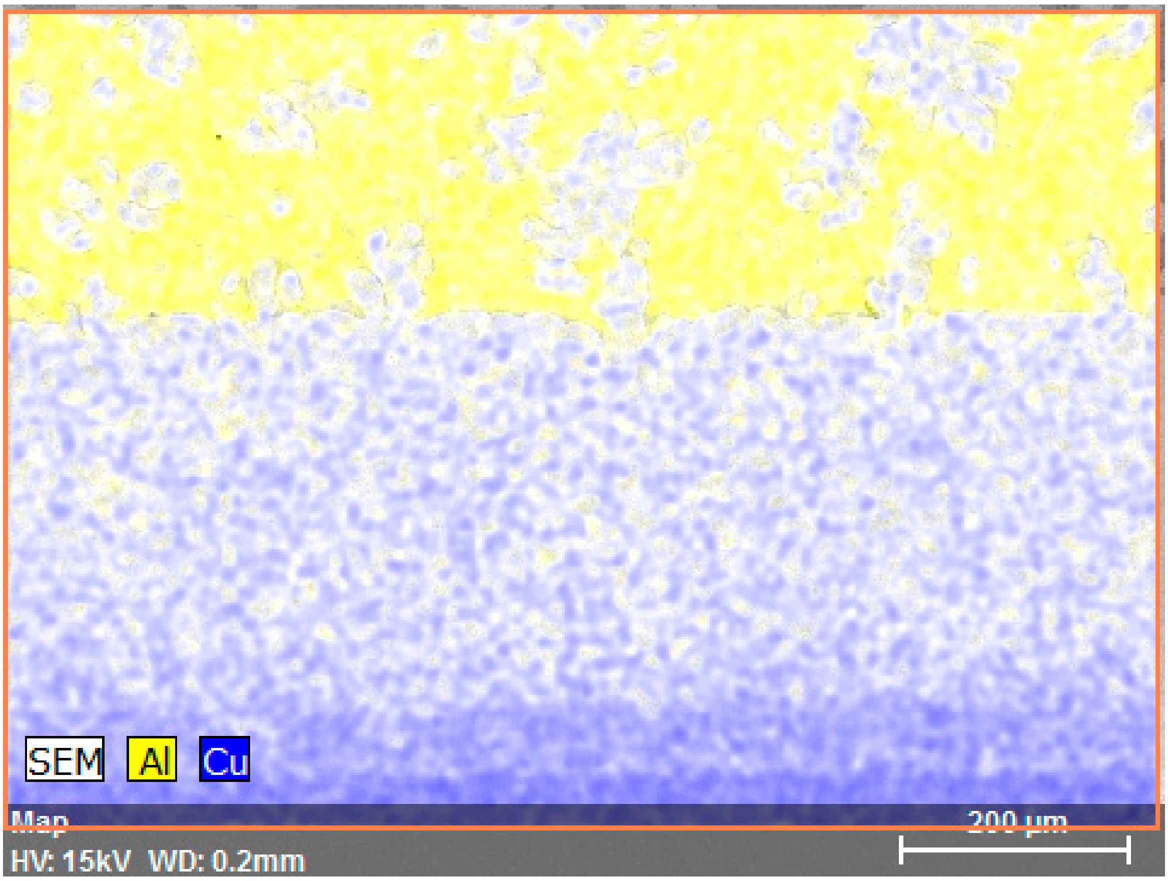

MapDate:15-Sep-25 2:32:08 PMImage size:480 x 360

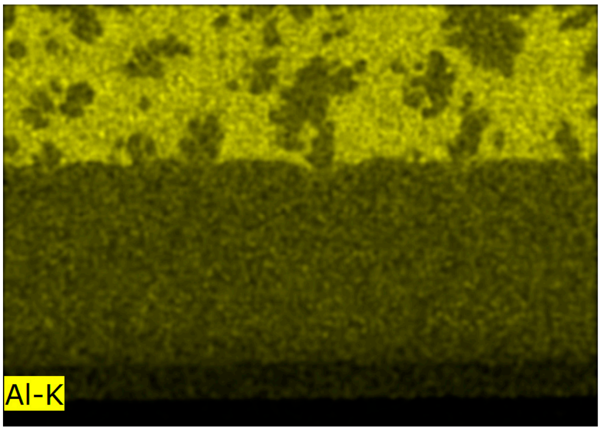

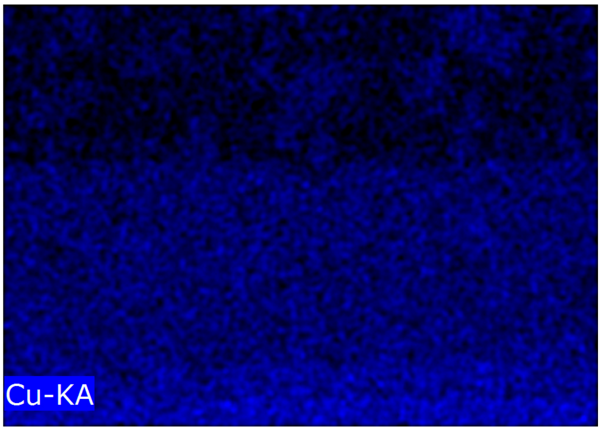

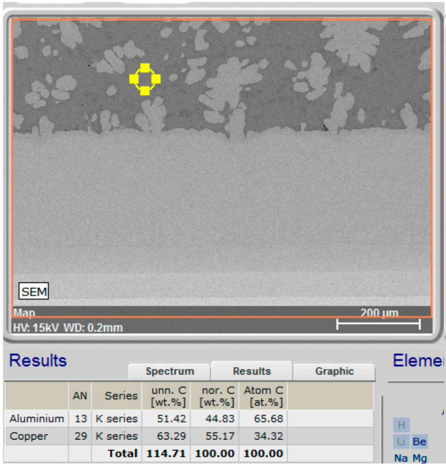

	AN	Series	unn. C [wt.%]	nor. C [wt.%]	Atom C [at.%]
Aluminium	13	K series	51.42	44.83	65.68
Copper	29	K series	63.29	55.17	34.32
		Total	114.71	100.00	100.00

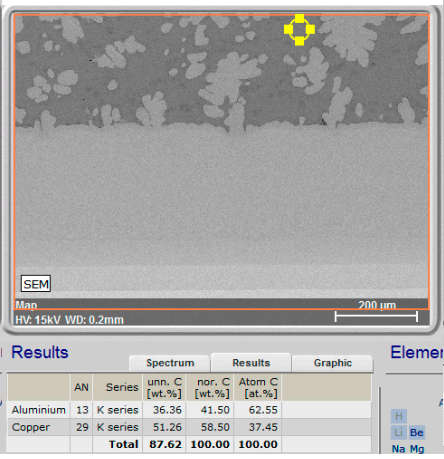

	AN	Series	unn. C [wt.%]	nor. C [wt.%]	Atom C [at.%]
Aluminium	13	K series	36.36	41.50	62.55
Copper	29	K series	51.26	58.50	37.45
		Total	87.62	100.00	100.00

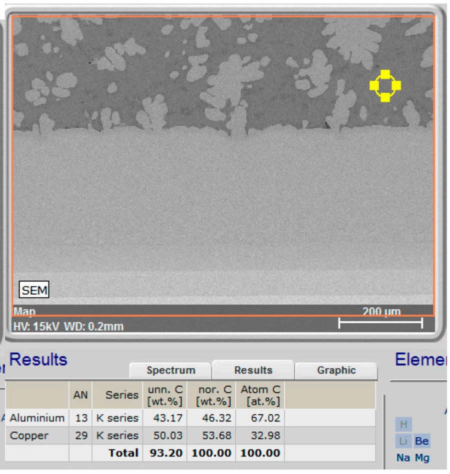

	AN	Series	unn. C [wt.%]	nor. C [wt.%]	Atom C [at.%]
Aluminium	13	K series	43.17	46.32	67.02
Copper	29	K series	50.03	53.68	32.98
		Total	93.20	100.00	100.00

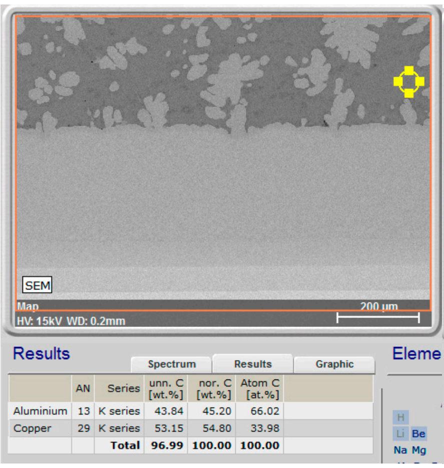

	AN	Series	unn. C [wt.%]	nor. C [wt.%]	Atom C [at.%]
Aluminium	13	K series	43.84	45.20	66.02
Copper	29	K series	53.15	54.80	33.98
		Total	96.99	100.00	100.00

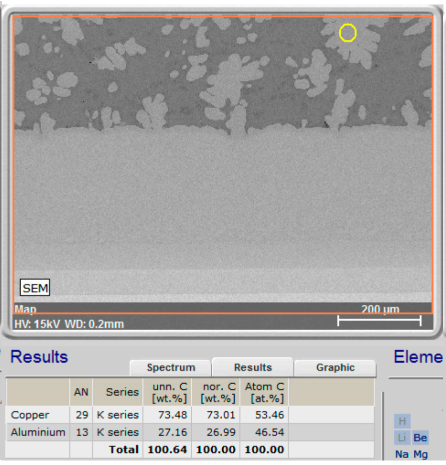

	AN	Series	unn. C [wt.%]	nor. C [wt.%]	Atom C [at.%]
Copper	29	K series	73.48	73.01	53.46
Aluminium	13	K series	27.16	26.99	46.54
		Total	100.64	100.00	100.00

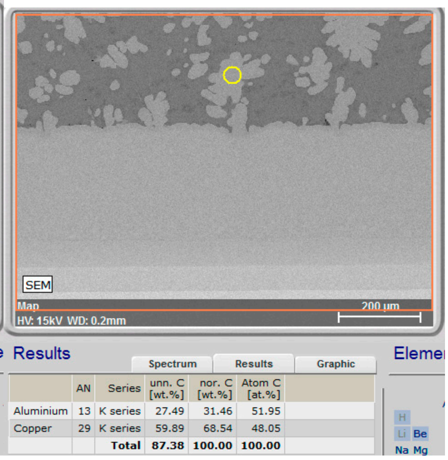

	AN	Series	unn. C [wt.%]	nor. C [wt.%]	Atom C [at.%]
Aluminium	13	K series	27.49	31.46	51.95
Copper	29	K series	59.89	68.54	48.05
		Total	87.38	100.00	100.00

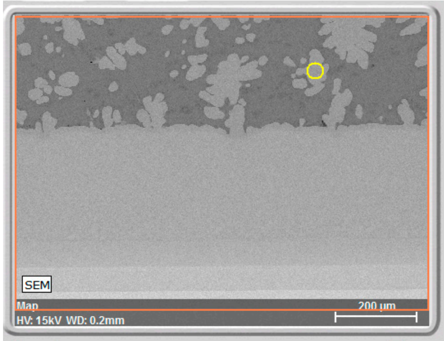

Results

	AN	Series	unn. C [wt.%]	nor. C [wt.%]	Atom C [at.%]
Copper	29	K series	84.59	75.05	56.09
Aluminium	13	K series	28.12	24.95	43.91
		Total	112.71	100.00	100.00

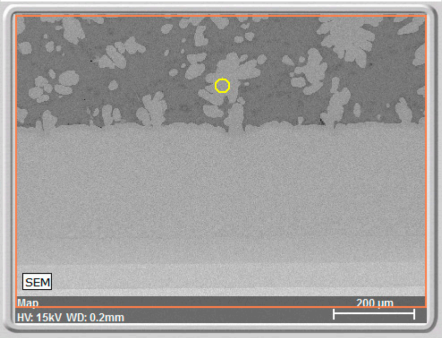

Results

	AN	Series	unn. C [wt.%]	nor. C [wt.%]	Atom C [at.%]
Copper	29	K series	54.25	71.27	51.30
Aluminium	13	K series	21.87	28.73	48.70
		Total	76.11	100.00	100.00

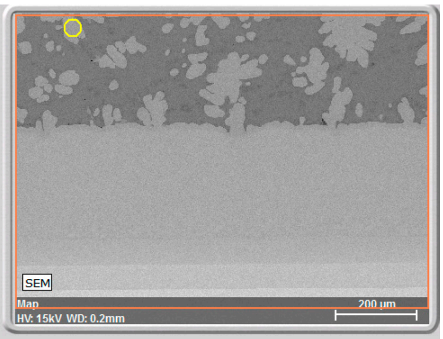

Results

	AN	Series	unn. C [wt.%]	nor. C [wt.%]	Atom C [at.%]
Copper	29	K series	65.34	70.48	50.34
Aluminium	13	K series	27.37	29.52	49.66
		Total	92.71	100.00	100.00

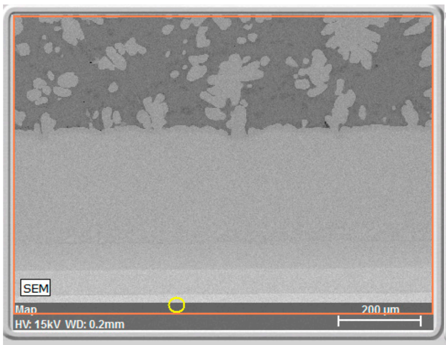

Results

	AN	Series	unn. C [wt.%]	nor. C [wt.%]	Atom C [at.%]
Copper	29	K series	96.18	99.44	98.69
Aluminium	13	K series	0.54	0.56	1.31
		Total	96.72	100.00	100.00

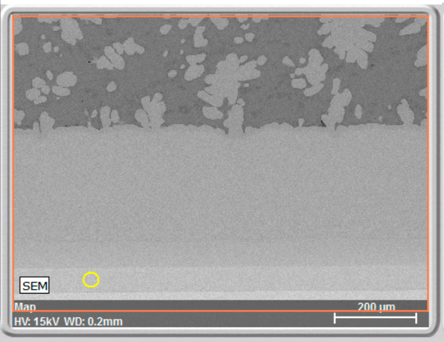

Results

	AN	Series	unn. C [wt.%]	nor. C [wt.%]	Atom C [at.%]
Copper	29	K series	91.09	87.92	75.55
Aluminium	13	K series	12.52	12.08	24.45
		Total	103.61	100.00	100.00

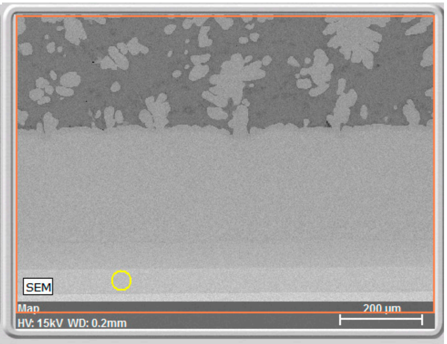

Results

	AN	Series	unn. C [wt.%]	nor. C [wt.%]	Atom C [at.%]
Copper	29	K series	75.75	87.28	74.44
Aluminium	13	K series	11.04	12.72	25.56
		Total	86.79	100.00	100.00

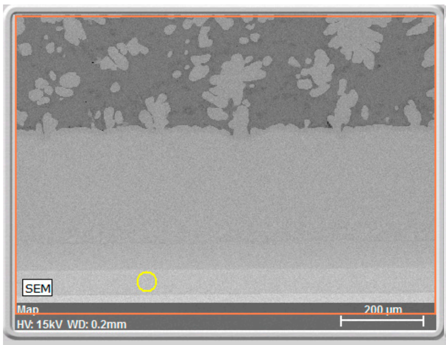

Results

	AN	Series	unn. C [wt.%]	nor. C [wt.%]	Atom C [at.%]
Copper	29	K series	85.10	88.96	77.38
Aluminium	13	K series	10.57	11.04	22.62
		Total	95.67	100.00	100.00

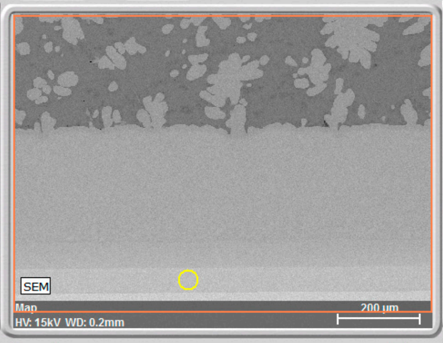

Results

	AN	Series	unn. C [wt.%]	nor. C [wt.%]	Atom C [at.%]
Copper	29	K series	70.66	86.51	73.14
Aluminium	13	K series	11.02	13.49	26.86
		Total	81.68	100.00	100.00

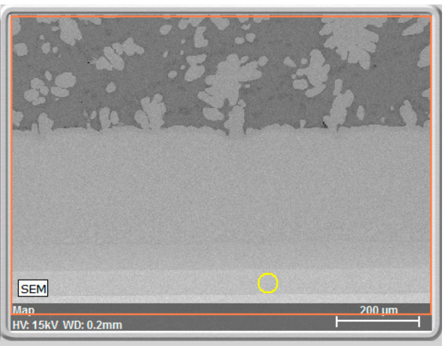

Results

	AN	Series	unn. C [wt.%]	nor. C [wt.%]	Atom C [at.%]
Copper	29	K series	80.14	87.26	74.42
Aluminium	13	K series	11.70	12.74	25.58
		Total	91.83	100.00	100.00

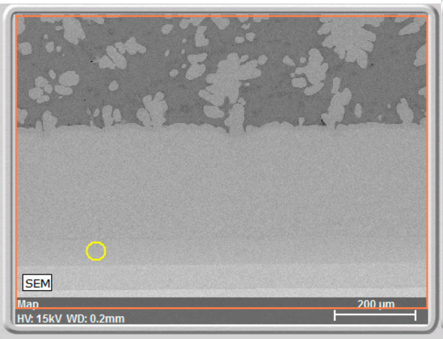

Results

Spectrum | Results | Graphic

	AN	Series	unn. C [wt.%]	nor. C [wt.%]	Atom C [at.%]
Copper	29	K series	89.23	81.93	65.81
Aluminium	13	K series	19.68	18.07	34.19
		Total	108.91	100.00	100.00

Elemer

H Li Be Na Mg

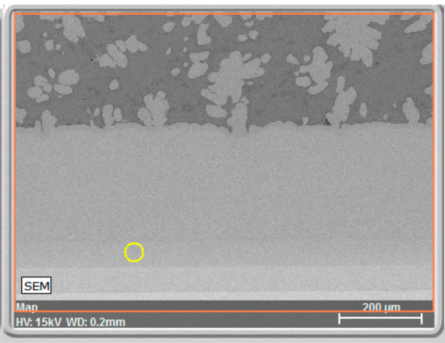

Results

Spectrum | Results | Graphic

	AN	Series	unn. C [wt.%]	nor. C [wt.%]	Atom C [at.%]
Copper	29	K series	71.43	80.13	63.13
Aluminium	13	K series	17.71	19.87	36.87
		Total	89.15	100.00	100.00

Eleme

H Li Be Na Mg

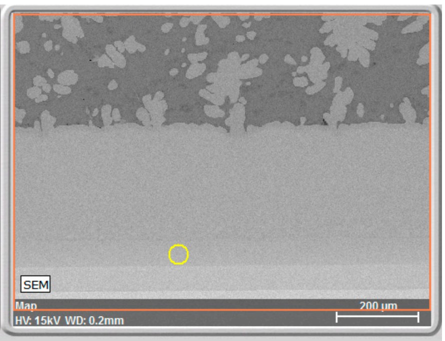

Results

Spectrum | Results | Graphic

	AN	Series	unn. C [wt.%]	nor. C [wt.%]	Atom C [at.%]
Copper	29	K series	72.79	80.83	64.17
Aluminium	13	K series	17.26	19.17	35.83
		Total	90.05	100.00	100.00

Eleme

H Li Be Na Mg

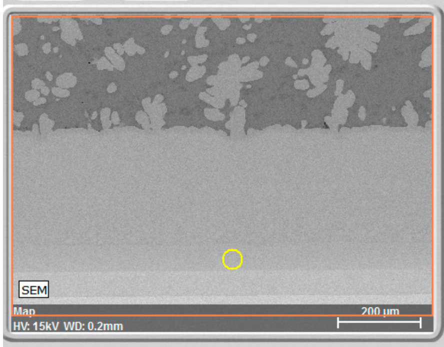

Results

Spectrum | Results | Graphic

	AN	Series	unn. C [wt.%]	nor. C [wt.%]	Atom C [at.%]
Copper	29	K series	69.25	79.67	62.46
Aluminium	13	K series	17.67	20.33	37.54
		Total	86.92	100.00	100.00

Eleme

H Li Be Na Mg

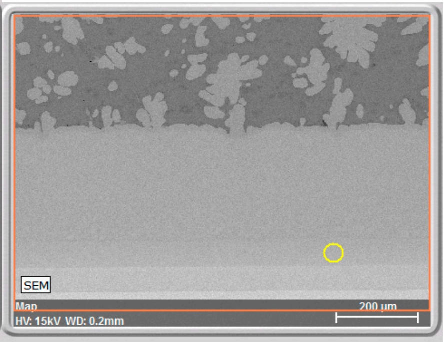

Results

Spectrum | Results | Graphic

	AN	Series	unn. C [wt.%]	nor. C [wt.%]	Atom C [at.%]
Copper	29	K series	94.10	83.11	67.64
Aluminium	13	K series	19.12	16.89	32.36
		Total	113.22	100.00	100.00

Elemer

H Li Be Na Mg
