# Supplementary material for: Effect of Ni Addition on the Solidification of Liquid Al and Solid Cu Diffusion Couples
Source: Materials (Basel). 2025 Dec 18;18(24):5689. doi: 10.3390/ma18245689 (PMC12735080; doi:10.3390/ma18245689)
Supplement: Supplementary file 1 [file materials-18-05689-s001.zip › Supplementary Figures/Files S1/Al-Ni 1800s diffusion zone/EDS 1.pdf]

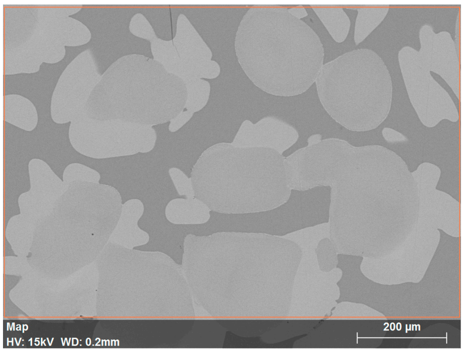

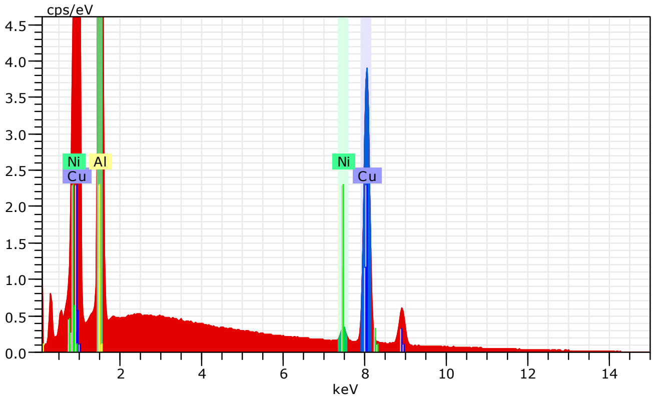

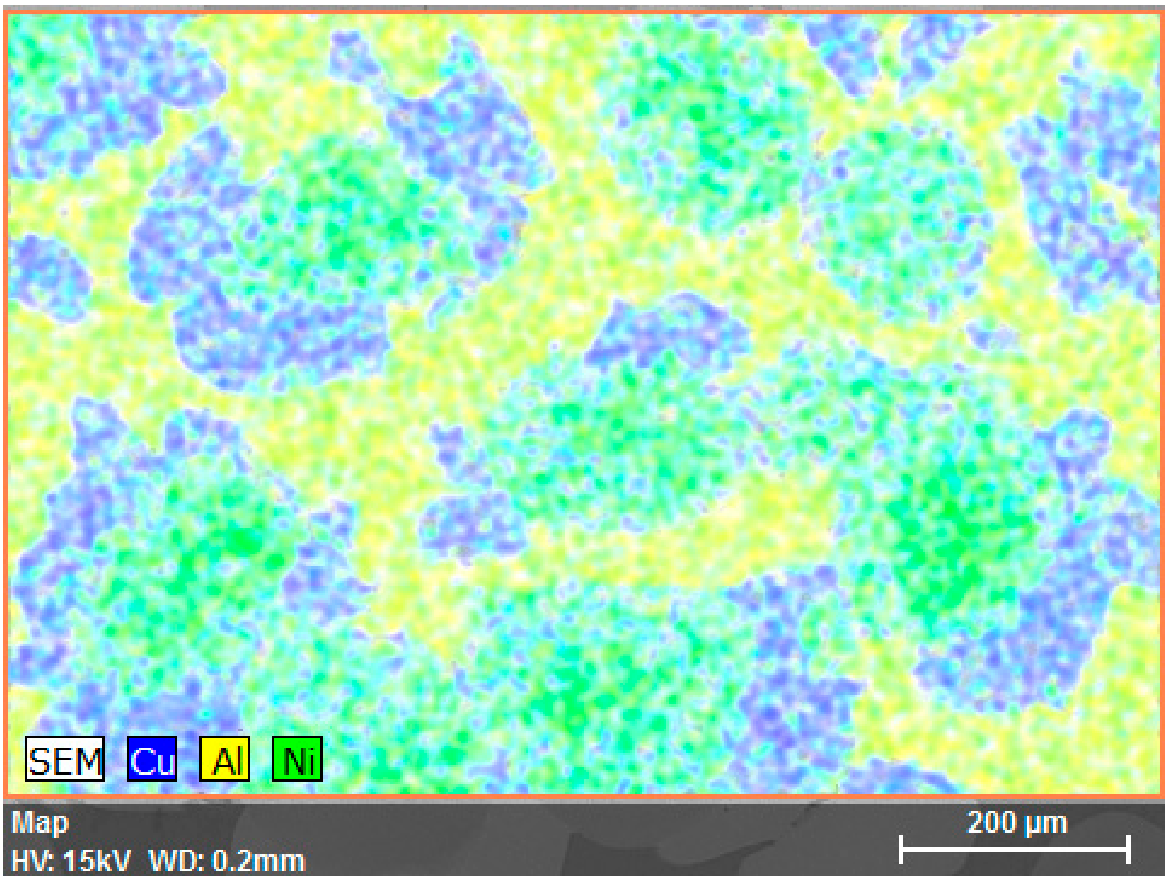

MapDate:07-Aug-25 5:38:35 PMImage size:480 x 360

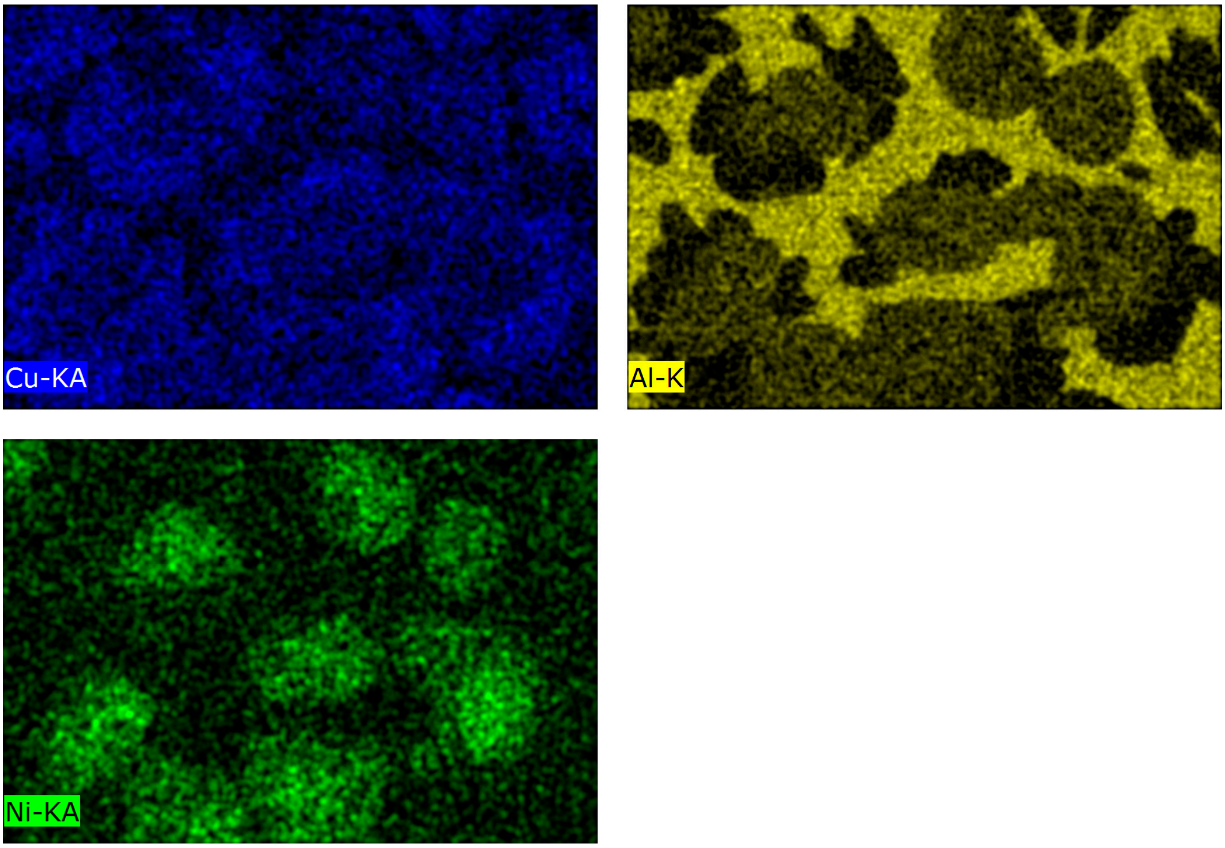

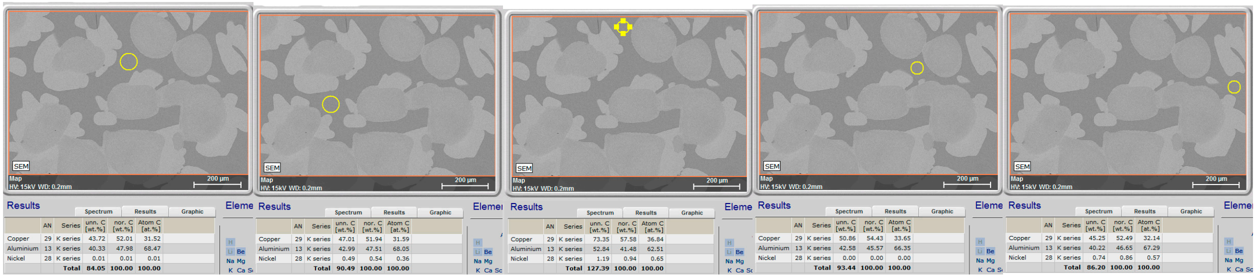

	AN	Series	unn. C [wt.%]	nor. C [wt.%]	Atom C [at.%]
Copper	29	K series	43.72	52.01	31.52
Aluminium	13	K series	40.33	47.98	68.47
Nickel	28	K series	0.01	0.01	0.01
		Total	84.05	100.00	100.00

	AN	Series	unn. C [wt.%]	nor. C [wt.%]	Atom C [at.%]
Copper	29	K series	47.01	51.94	31.59
Aluminium	13	K series	42.99	47.51	68.05
Nickel	28	K series	0.49	0.54	0.36
		Total	90.49	100.00	100.00

	AN	Series	unn. C [wt.%]	nor. C [wt.%]	Atom C [at.%]
Copper	29	K series	73.35	57.58	36.84
Aluminium	13	K series	52.84	41.48	62.51
Nickel	28	K series	1.19	0.94	0.65
		Total	127.39	100.00	100.00

	AN	Series	unn. C [wt.%]	nor. C [wt.%]	Atom C [at.%]
Copper	29	K series	50.86	54.43	33.65
Aluminium	13	K series	42.58	45.57	66.35
Nickel	28	K series	0.00	0.00	0.00
		Total	93.44	100.00	100.00

	AN	Series	unn. C [wt.%]	nor. C [wt.%]	Atom C [at.%]
Copper	29	K series	45.25	52.49	32.14
Aluminium	13	K series	40.22	46.65	67.29
Nickel	28	K series	0.74	0.86	0.57
		Total	86.20	100.00	100.00

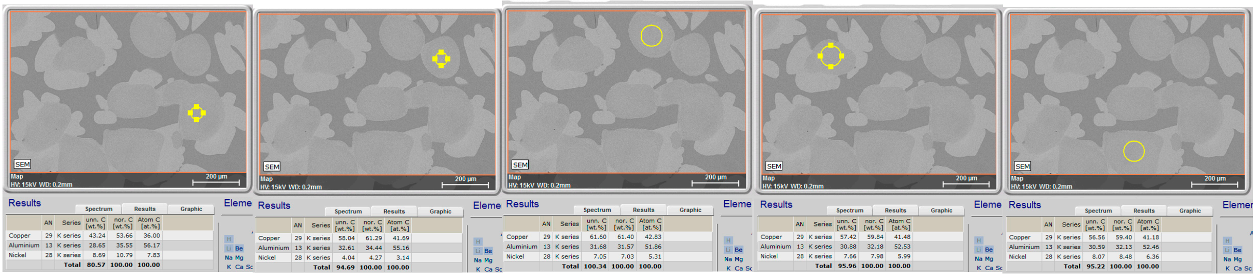

	AN	Series	unn. C [wt.%]	nor. C [wt.%]	Atom C [at.%]
Copper	29	K series	43.24	53.66	36.00
Aluminium	13	K series	28.65	35.55	56.17
Nickel	28	K series	8.69	10.79	7.83
		Total	80.57	100.00	100.00

	AN	Series	unn. C [wt.%]	nor. C [wt.%]	Atom C [at.%]
Copper	29	K series	58.04	61.29	41.69
Aluminium	13	K series	32.61	34.44	55.16
Nickel	28	K series	4.04	4.27	3.14
		Total	94.69	100.00	100.00

	AN	Series	unn. C [wt.%]	nor. C [wt.%]	Atom C [at.%]
Copper	29	K series	61.60	61.40	42.83
Aluminium	13	K series	31.68	31.57	51.86
Nickel	28	K series	7.05	7.03	5.31
		Total	100.34	100.00	100.00

	AN	Series	unn. C [wt.%]	nor. C [wt.%]	Atom C [at.%]
Copper	29	K series	57.42	59.84	41.48
Aluminium	13	K series	30.88	32.18	52.53
Nickel	28	K series	7.66	7.98	5.99
		Total	95.96	100.00	100.00

	AN	Series	unn. C [wt.%]	nor. C [wt.%]	Atom C [at.%]
Copper	29	K series	56.56	59.40	41.18
Aluminium	13	K series	30.59	32.13	52.46
Nickel	28	K series	8.07	8.48	6.36
		Total	95.22	100.00	100.00

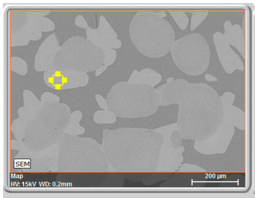

Results

Spectrum | Results | Graphic

	AN	Series	unn. C [wt.%]	nor. C [wt.%]	Atom C [at.%]
Copper	29	K series	77.61	73.13	54.16
Aluminium	13	K series	27.35	25.77	44.95
Nickel	28	K series	1.17	1.10	0.88
		**Total**	**106.13**	**100.00**	**100.00**

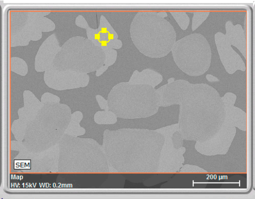

Results

Spectrum | Results | Graphic

	AN	Series	unn. C [wt.%]	nor. C [wt.%]	Atom C [at.%]
Copper	29	K series	72.29	70.04	50.22
Aluminium	13	K series	29.99	29.06	49.08
Nickel	28	K series	0.93	0.90	0.70
		**Total**	**103.21**	**100.00**	**100.00**

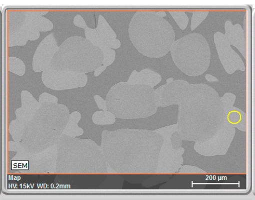

Results

Spectrum | Results | Graphic

	AN	Series	unn. C [wt.%]	nor. C [wt.%]	Atom C [at.%]
Copper	29	K series	55.18	68.57	48.45
Aluminium	13	K series	24.62	30.59	50.91
Nickel	28	K series	0.68	0.84	0.64
		**Total**	**80.48**	**100.00**	**100.00**

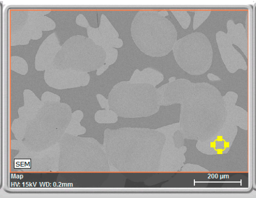

Results

Spectrum | Results | Graphic

	AN	Series	unn. C [wt.%]	nor. C [wt.%]	Atom C [at.%]
Copper	29	K series	59.92	70.52	50.67
Aluminium	13	K series	24.54	28.88	48.86
Nickel	28	K series	0.51	0.60	0.47
		**Total**	**84.97**	**100.00**	**100.00**

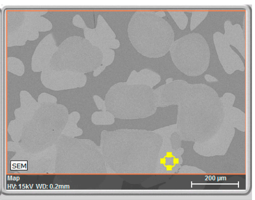

Results

Spectrum | Results | Graphic

	AN	Series	unn. C [wt.%]	nor. C [wt.%]	Atom C [at.%]
Copper	29	K series	73.22	74.01	54.97
Aluminium	13	K series	25.27	25.54	44.68
Nickel	28	K series	0.44	0.44	0.36
		**Total**	**98.93**	**100.00**	**100.00**
