# Supplementary material for: Effect of Ni Addition on the Solidification of Liquid Al and Solid Cu Diffusion Couples
Source: Materials (Basel). 2025 Dec 18;18(24):5689. doi: 10.3390/ma18245689 (PMC12735080; doi:10.3390/ma18245689)
Supplement: Supplementary file 1 [file materials-18-05689-s001.zip › Supplementary Figures/Files S1/Al-Ni 1800s diffusion zone/EDS 2.pdf]

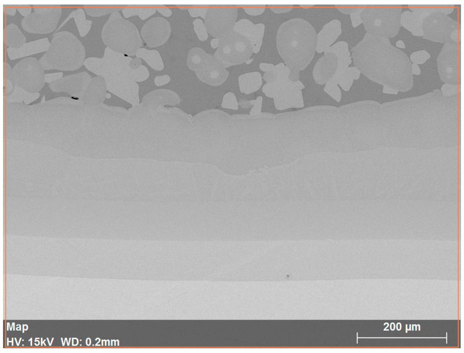

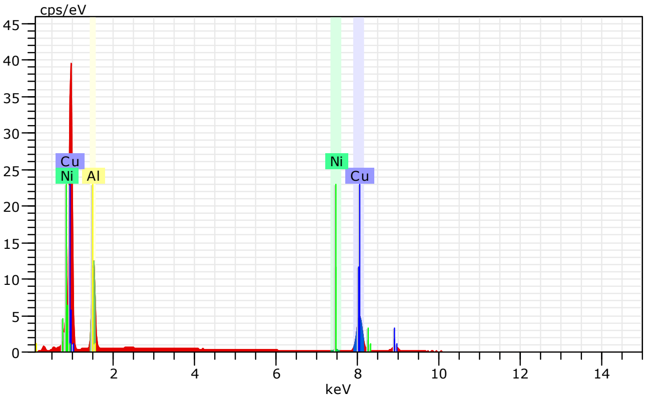

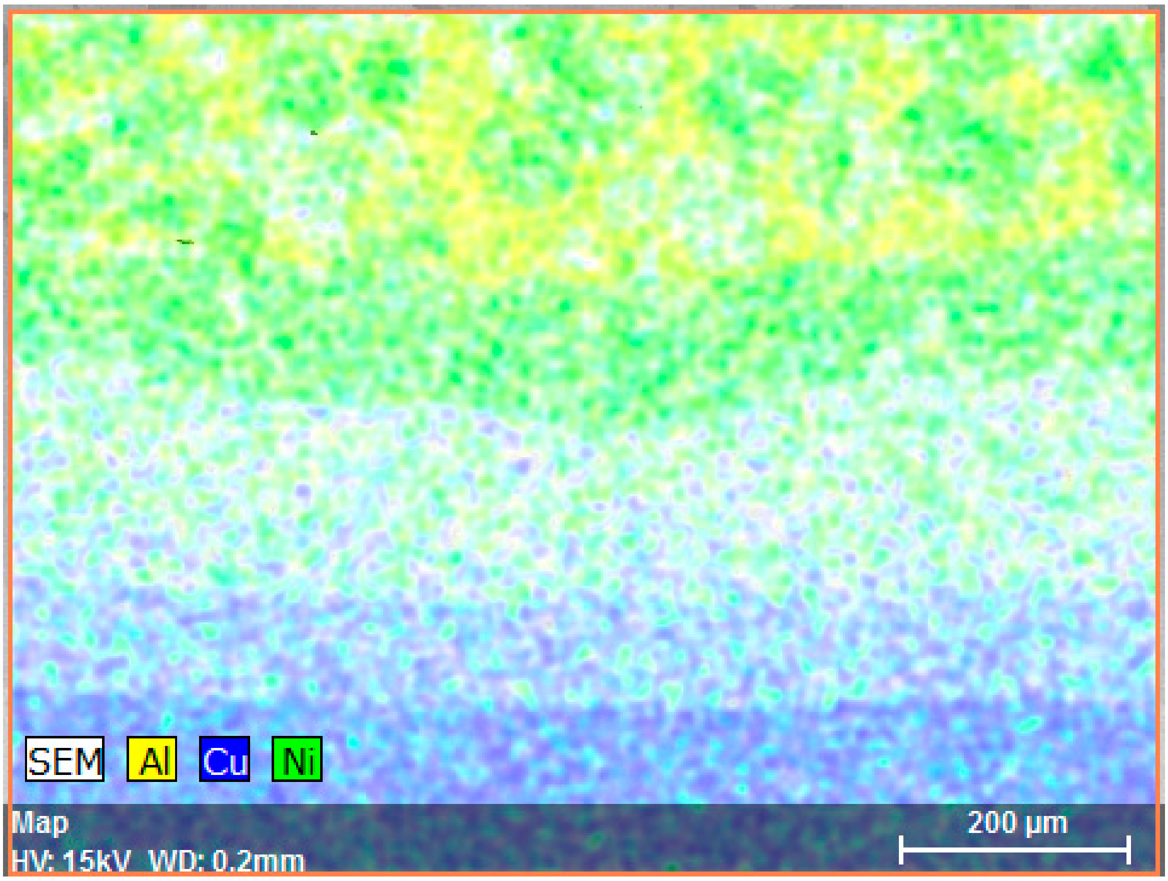

MapDate:15-Sep-25 4:39:06 PMImage size:480 x 360

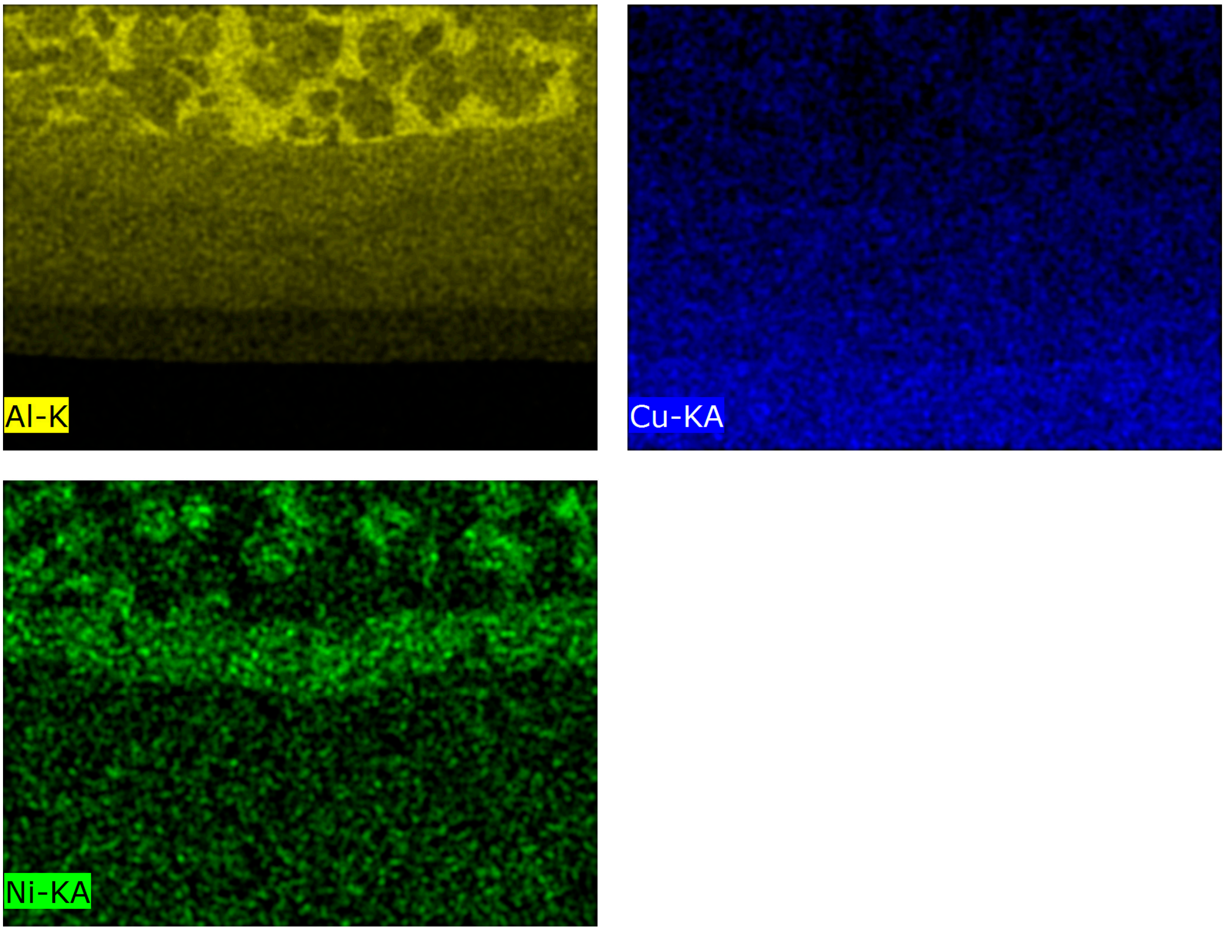

Cu side

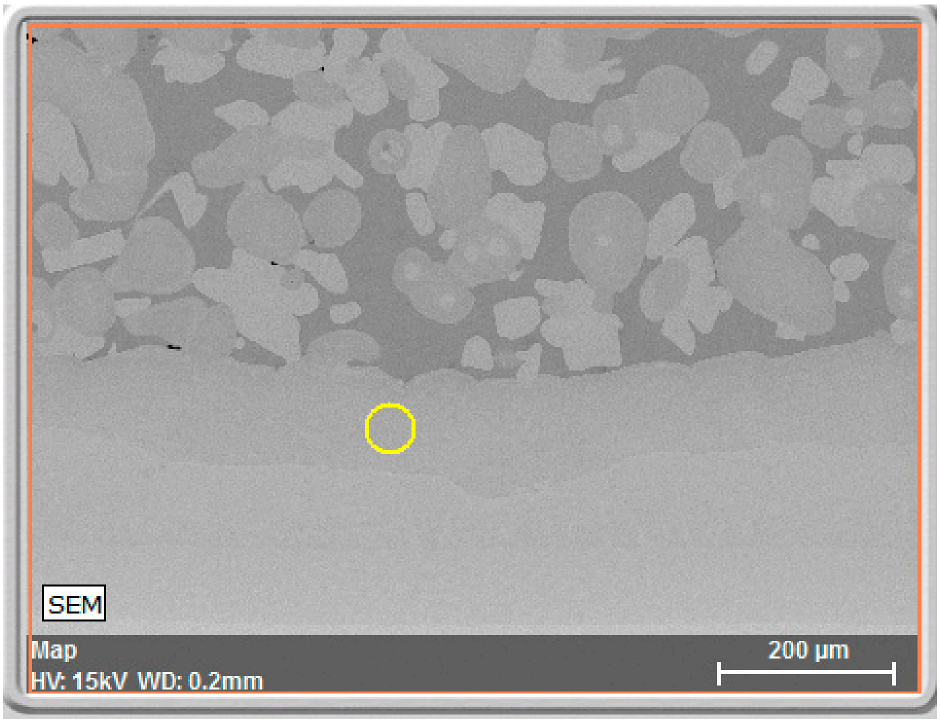

Results

Spectrum | Results | Graphic

	AN	Series	unn. C [wt.%]	nor. C [wt.%]	Atom C [at.%]
Copper	29	K series	57.42	66.83	48.14
Aluminium	13	K series	24.37	28.36	48.11
Nickel	28	K series	4.14	4.81	3.75
		**Total**	**85.92**	**100.00**	**100.00**

Eleme

H
Li Be
Na Mg
K Ca S

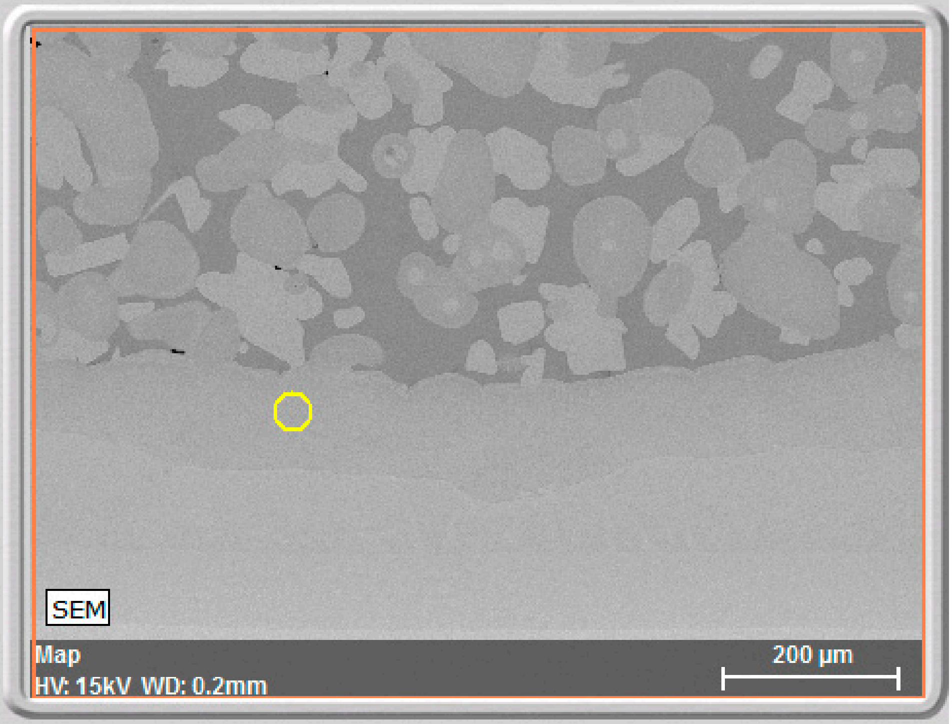

Results

Spectrum | Results | Graphic

	AN	Series	unn. C [wt.%]	nor. C [wt.%]	Atom C [at.%]
Aluminium	13	K series	25.38	30.00	50.09
Copper	29	K series	55.15	65.20	46.22
Nickel	28	K series	4.06	4.80	3.68
		**Total**	**84.59**	**100.00**	**100.00**

Eleme

H
Li Be
Na Mg
K Ca Sc

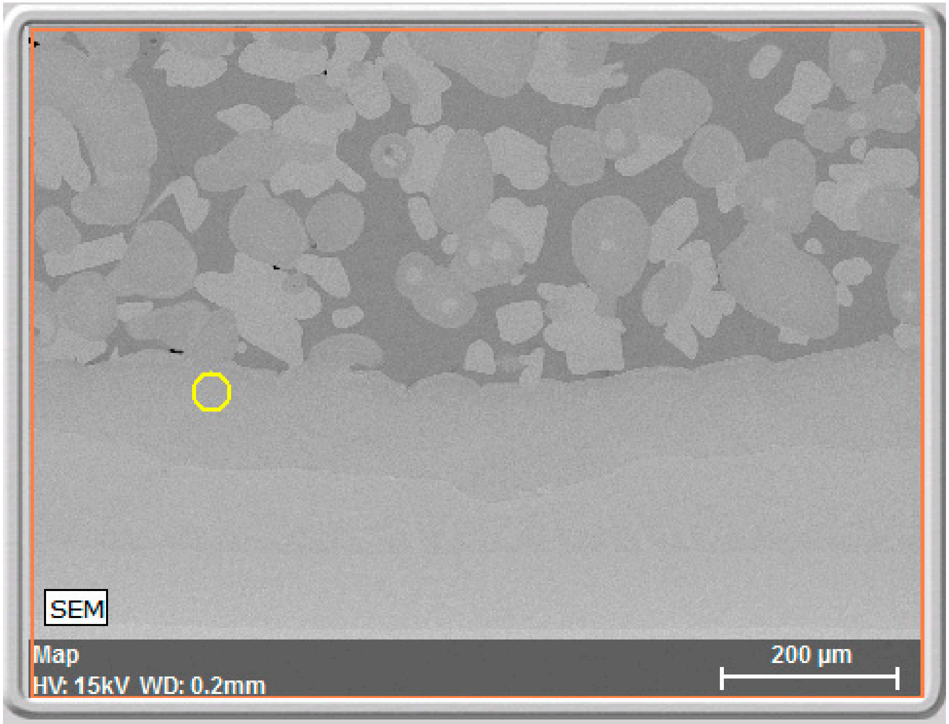

Results

Spectrum | Results | Graphic

	AN	Series	unn. C [wt.%]	nor. C [wt.%]	Atom C [at.%]
Aluminium	13	K series	32.05	30.09	50.21
Copper	29	K series	69.82	65.54	46.44
Nickel	28	K series	4.66	4.38	3.36
		**Total**	**106.54**	**100.00**	**100.00**

Eleme

H
Li Be
Na Mg
K Ca Sc

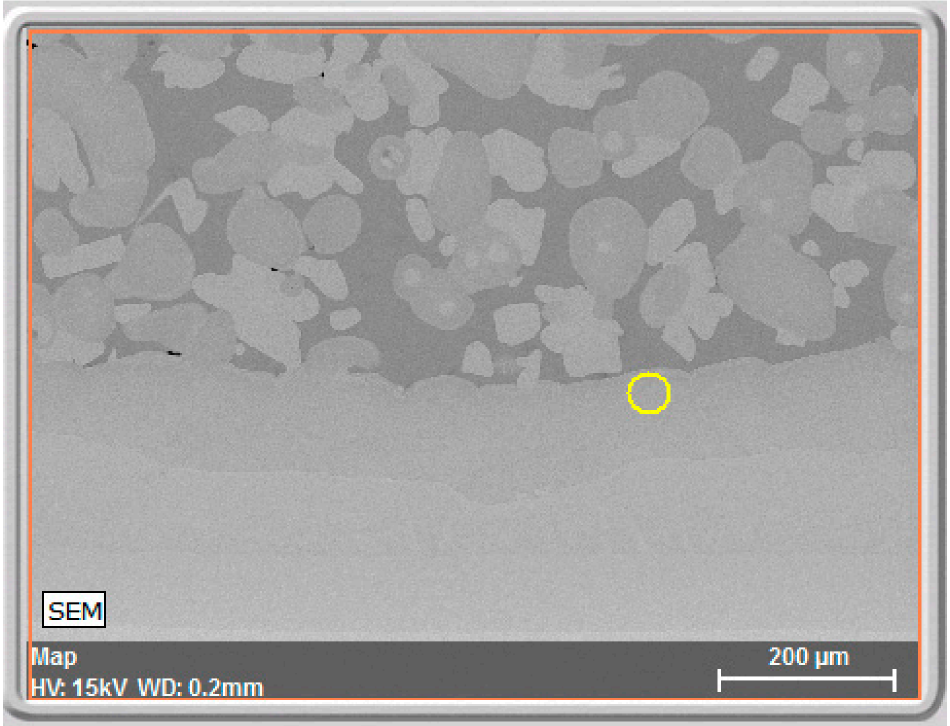

Results

Spectrum | Results | Graphic

	AN	Series	unn. C [wt.%]	nor. C [wt.%]	Atom C [at.%]
Aluminium	13	K series	26.96	31.04	51.36
Copper	29	K series	56.98	65.62	46.10
Nickel	28	K series	2.89	3.33	2.53
		**Total**	**86.83**	**100.00**	**100.00**

Elemen

H
Li Be
Na Mg
K Ca Sc

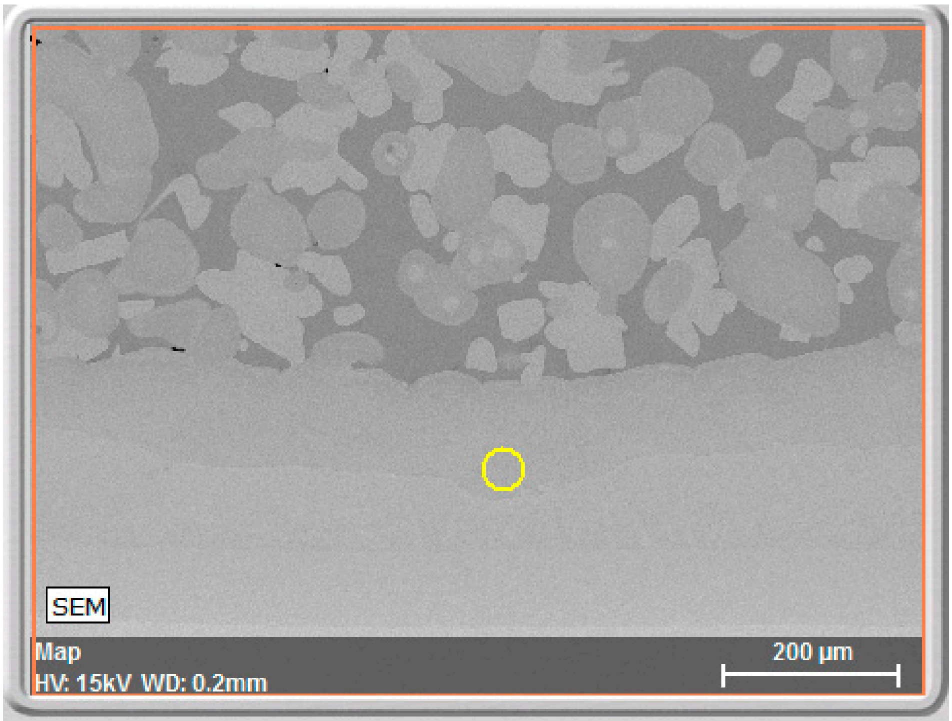

Results

Spectrum | Results | Graphic

	AN	Series	unn. C [wt.%]	nor. C [wt.%]	Atom C [at.%]
Copper	29	K series	66.88	70.90	52.69
Aluminium	13	K series	23.83	25.27	44.22
Nickel	28	K series	3.62	3.83	3.08
		**Total**	**94.34**	**100.00**	**100.00**

Eleme

H
Li Be
Na Mg
K Ca Sc

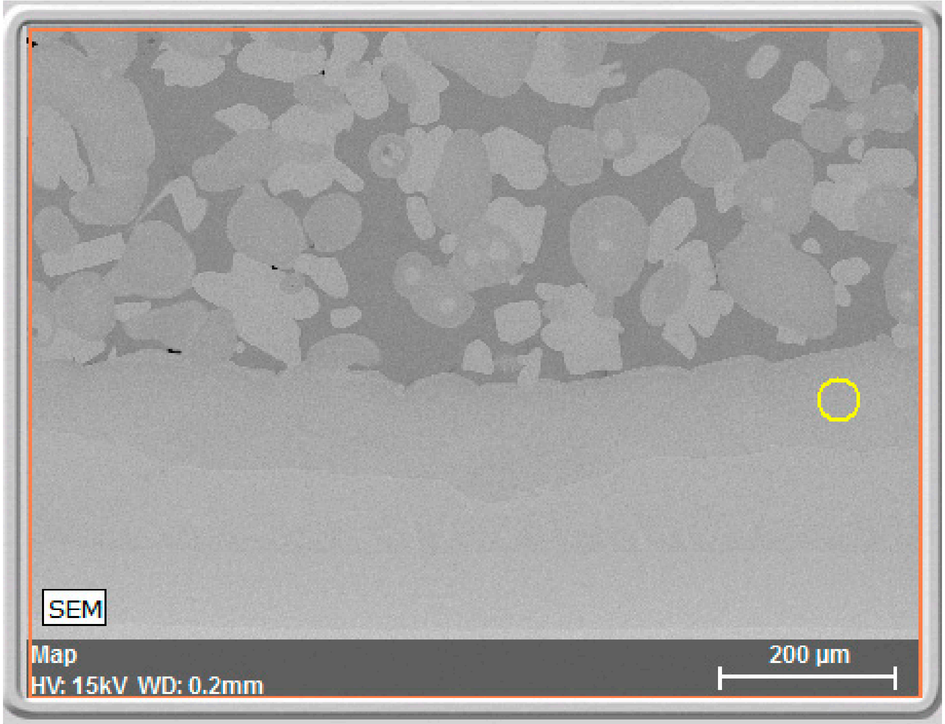

Results

Spectrum | Results | Graphic

	AN	Series	unn. C [wt.%]	nor. C [wt.%]	Atom C [at.%]
Copper	29	K series	69.12	69.36	50.90
Aluminium	13	K series	26.41	26.51	45.81
Nickel	28	K series	4.12	4.14	3.29
		**Total**	**99.65**	**100.00**	**100.00**

2nd layer

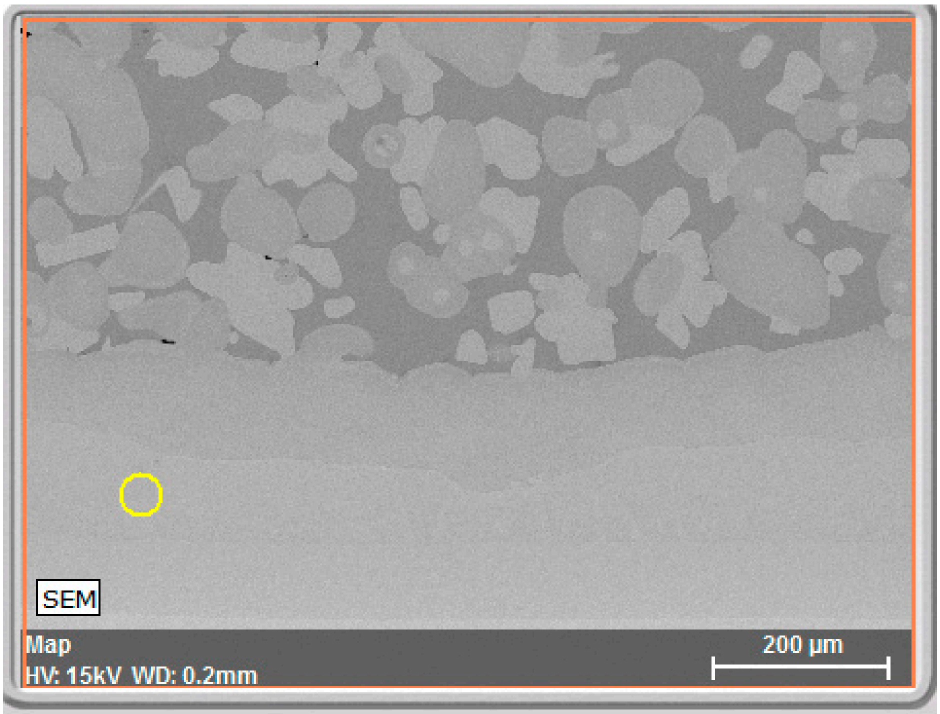

Results

Spectrum | Results | Graphic

	AN	Series	unn. C [wt.%]	nor. C [wt.%]	Atom C [at.%]
Copper	29	K series	60.71	75.48	56.75
Aluminium	13	K series	19.59	24.35	43.11
Nickel	28	K series	0.14	0.17	0.14
		**Total**	**80.44**	**100.00**	**100.00**

Eleme

H
Li Be
Na Mg
K Ca Sc

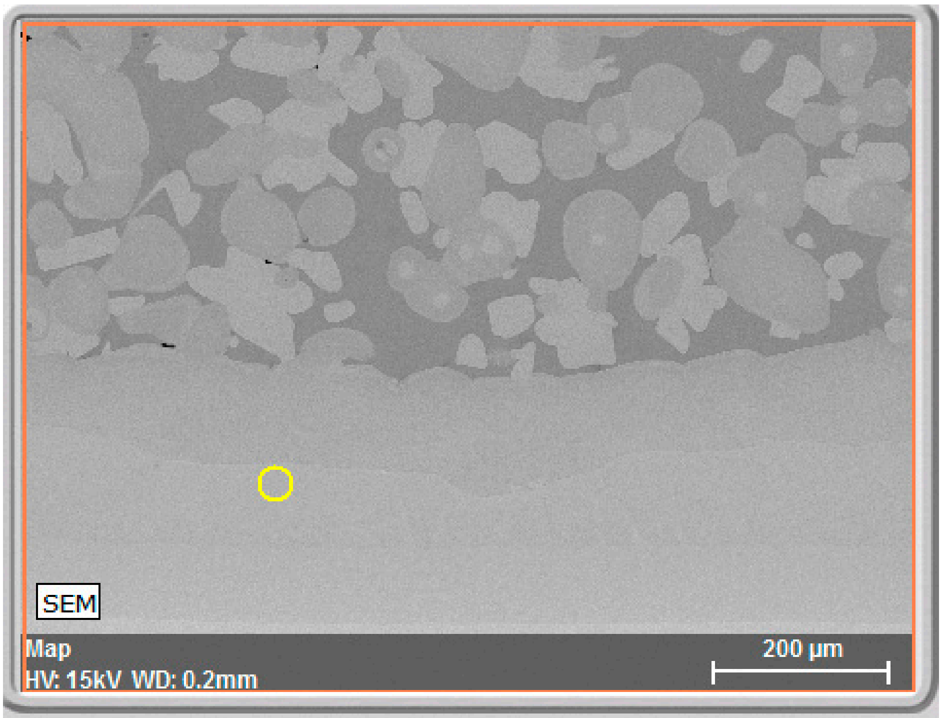

Results

Spectrum | Results | Graphic

	AN	Series	unn. C [wt.%]	nor. C [wt.%]	Atom C [at.%]
Copper	29	K series	65.02	75.80	57.08
Aluminium	13	K series	20.76	24.20	42.92
Nickel	28	K series	0.00	0.00	0.00
		**Total**	**85.78**	**100.00**	**100.00**

Eleme

H
Li Be
Na Mg
K Ca Sc

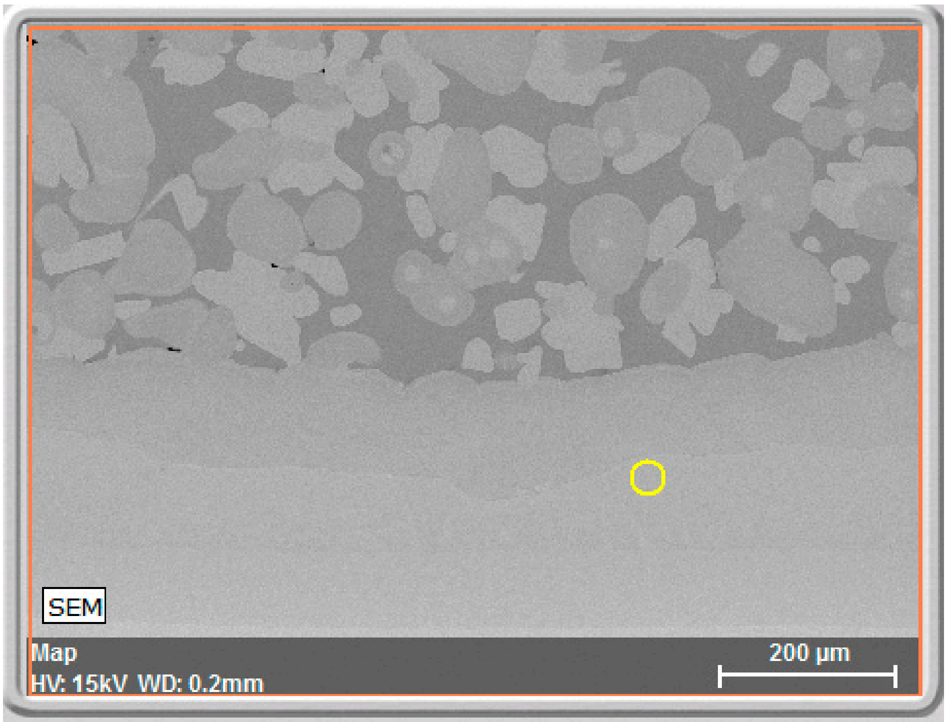

Results

Spectrum | Results | Graphic

	AN	Series	unn. C [wt.%]	nor. C [wt.%]	Atom C [at.%]
Copper	29	K series	81.17	79.42	62.11
Aluminium	13	K series	21.03	20.58	37.89
Nickel	28	K series	0.00	0.00	0.00
		**Total**	**102.20**	**100.00**	**100.00**

Eleme

H
Li Be
Na Mg
K Ca Sc

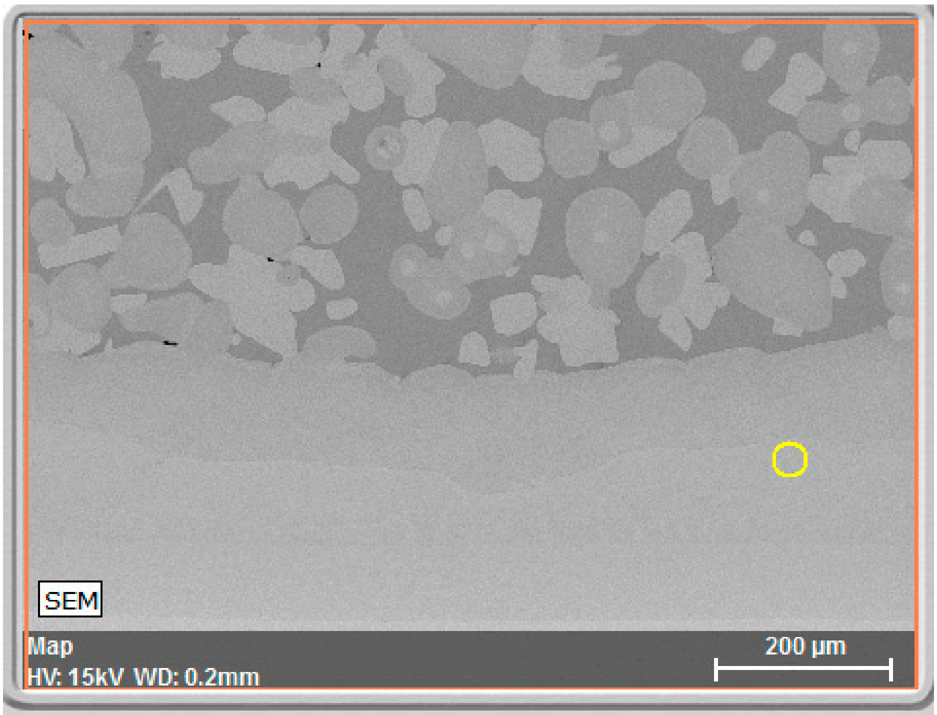

Results

Spectrum | Results | Graphic

	AN	Series	unn. C [wt.%]	nor. C [wt.%]	Atom C [at.%]
Copper	29	K series	71.40	76.86	58.51
Aluminium	13	K series	21.49	23.14	41.48
Nickel	28	K series	0.00	0.00	0.00
		**Total**	**92.90**	**100.00**	**100.00**

Eleme

H
Li Be
Na Mg
K Ca S

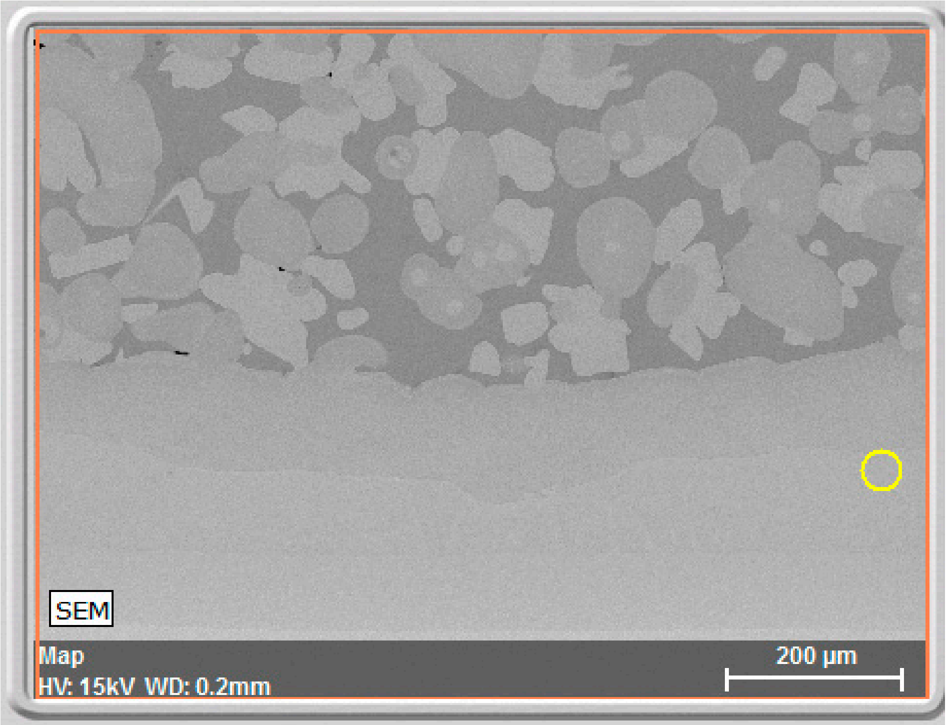

Results

Spectrum | Results | Graphic

	AN	Series	unn. C [wt.%]	nor. C [wt.%]	Atom C [at.%]
Copper	29	K series	77.90	78.68	61.05
Aluminium	13	K series	21.11	21.32	38.95
Nickel	28	K series	0.00	0.00	0.00
		**Total**	**99.01**	**100.00**	**100.00**

Eleme

H
Li Be
Na Mg
K Ca S

3rd layer

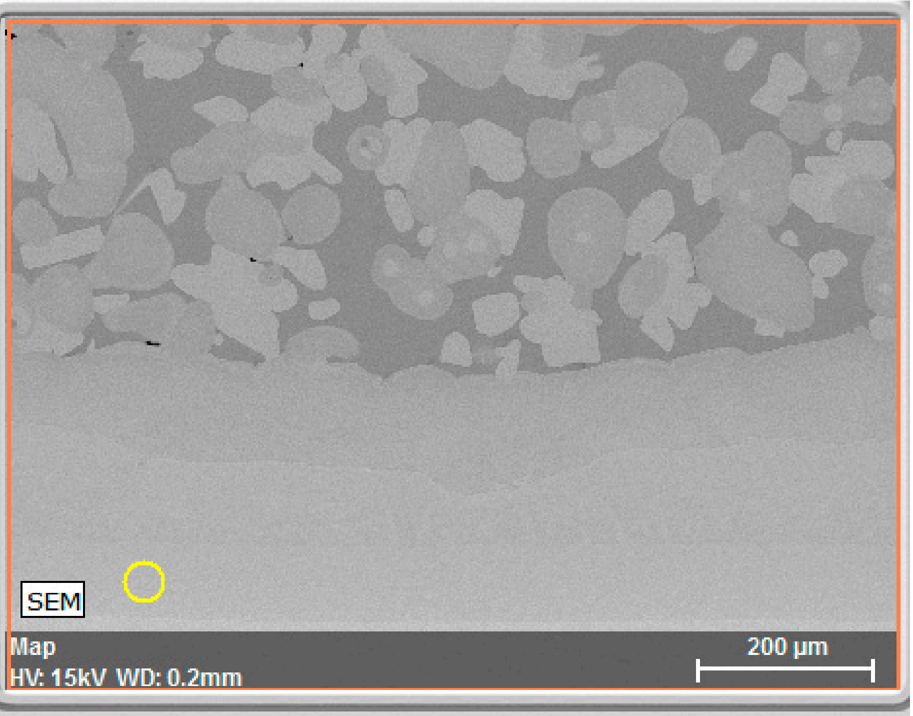

Results

Spectrum | Results | Graphic

	AN	Series	unn. C [wt.%]	nor. C [wt.%]	Atom C [at.%]
Copper	29	K series	90.11	82.53	67.07
Aluminium	13	K series	18.53	16.97	32.49
Nickel	28	K series	0.55	0.50	0.44
		**Total**	**109.19**	**100.00**	**100.00**

Eleme

H
Li Be
Na Mg
K Ca S

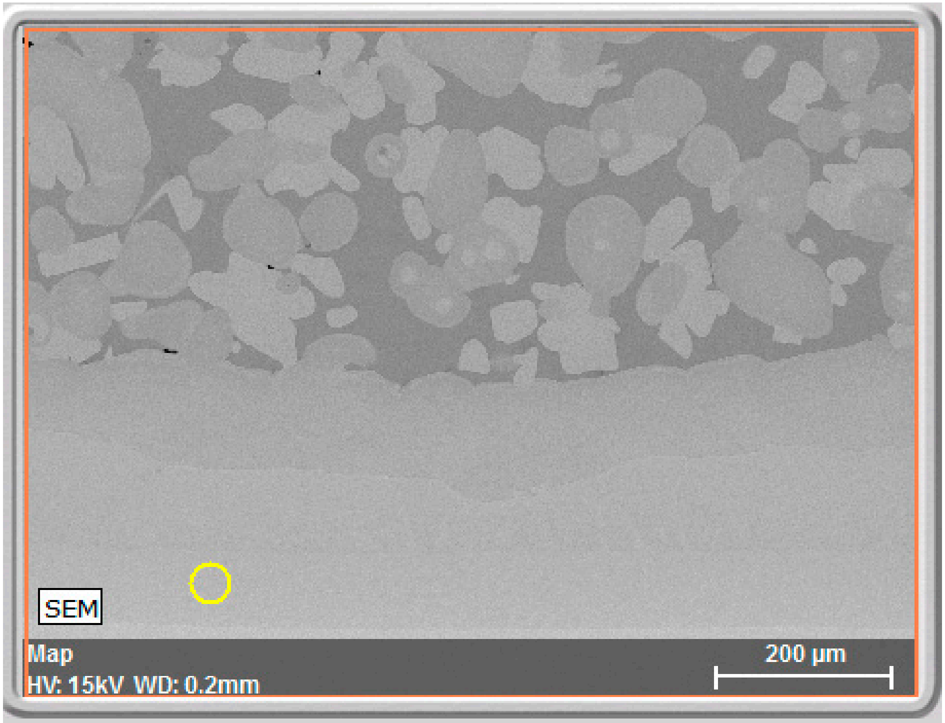

Results

Spectrum | Results | Graphic

	AN	Series	unn. C [wt.%]	nor. C [wt.%]	Atom C [at.%]
Copper	29	K series	69.10	79.41	62.09
Aluminium	13	K series	17.91	20.59	37.91
Nickel	28	K series	0.00	0.00	0.00
		Total	87.01	100.00	100.00

Eleme

H
Li Be
Na Mg
K Ca Sc

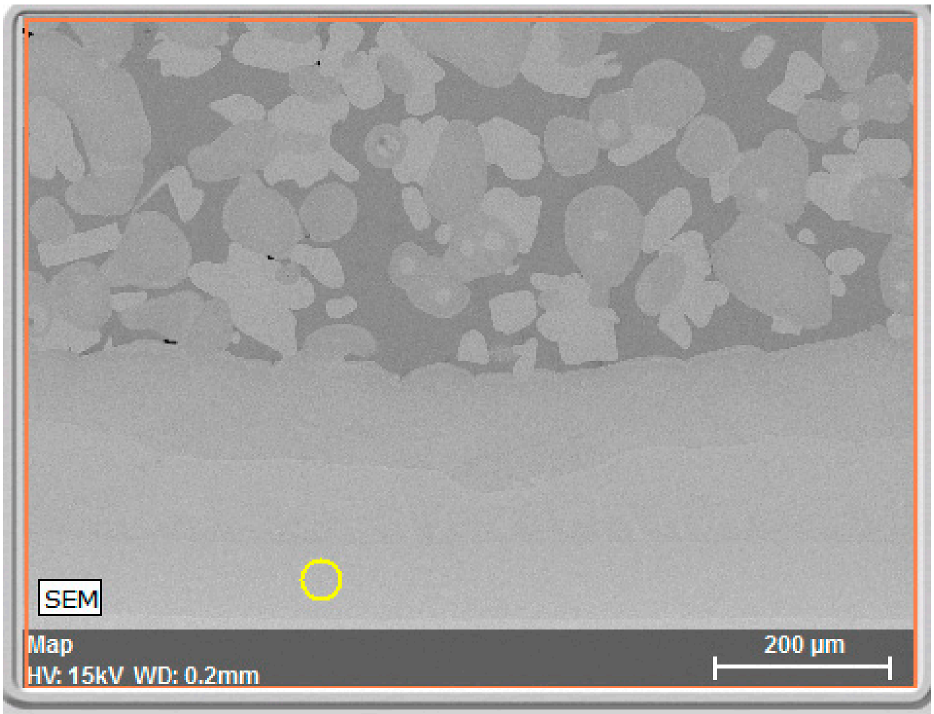

Results

Spectrum | Results | Graphic

	AN	Series	unn. C [wt.%]	nor. C [wt.%]	Atom C [at.%]
Copper	29	K series	74.97	81.06	64.98
Aluminium	13	K series	16.85	18.22	34.39
Nickel	28	K series	0.67	0.73	0.63
		**Total**	**92.48**	**100.00**	**100.00**

Eleme

H
Li Be
Na Mg
K Ca S

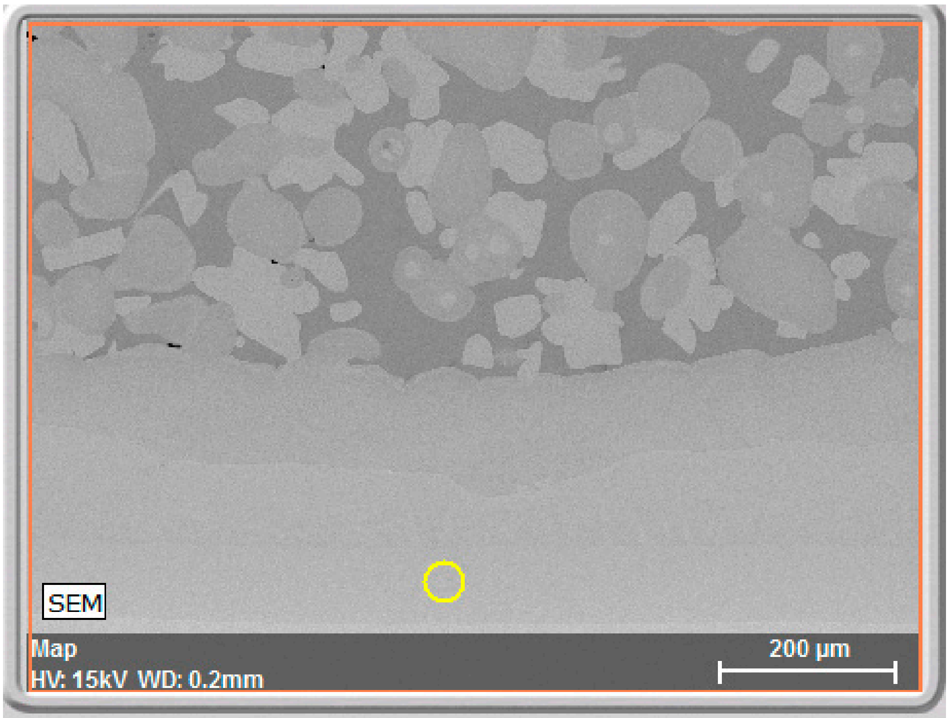

Results

	AN	Series	unn. C [wt.%]	nor. C [wt.%]	Atom C [at.%]
Copper	29	K series	85.10	79.42	62.29
Aluminium	13	K series	21.73	20.28	37.46
Nickel	28	K series	0.32	0.30	0.25
		**Total**	**107.15**	**100.00**	**100.00**

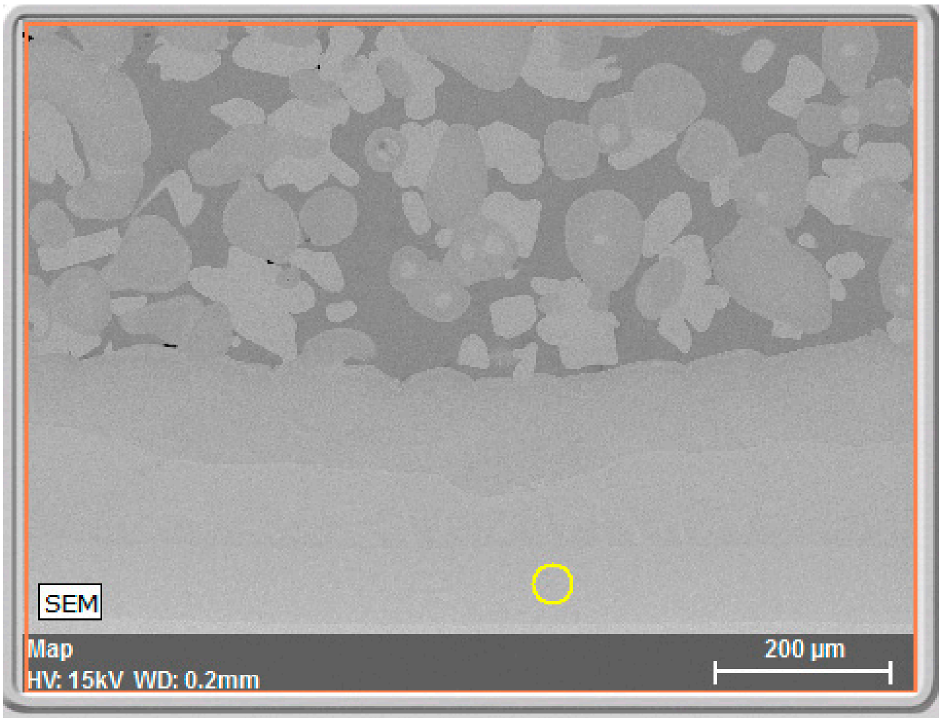

Results

Spectrum | Results | Graphic

	AN	Series	unn. C [wt.%]	nor. C [wt.%]	Atom C [at.%]
Copper	29	K series	64.64	76.27	57.71
Aluminium	13	K series	20.11	23.73	42.29
Nickel	28	K series	0.00	0.00	0.00
		**Total**	**84.76**	**100.00**	**100.00**

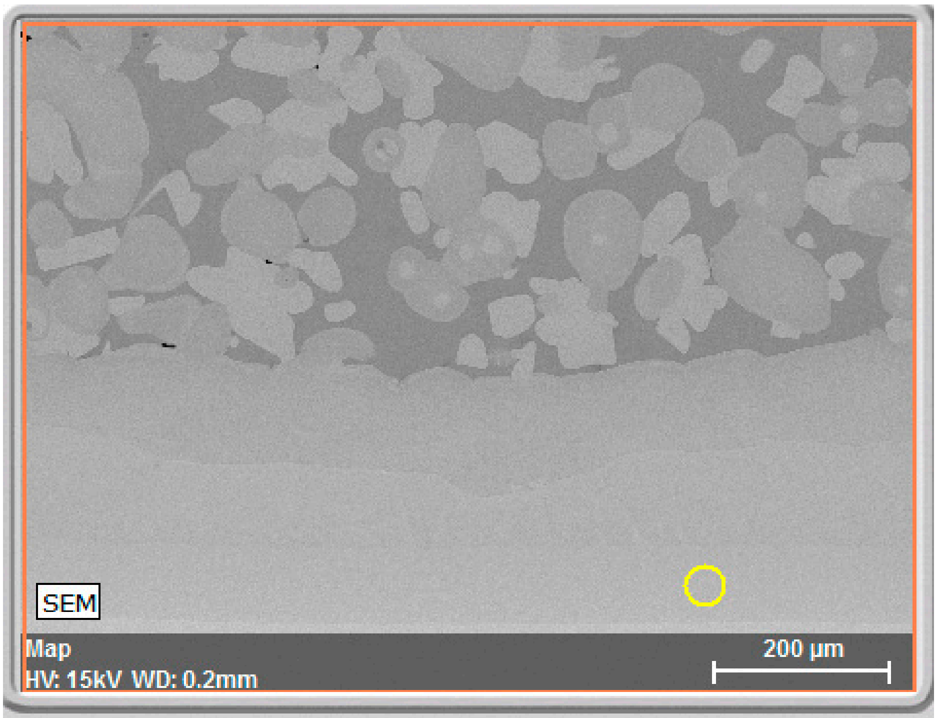

Results

Spectrum | Results | Graphic

	AN	Series	unn. C [wt.%]	nor. C [wt.%]	Atom C [at.%]
Copper	29	K series	71.00	80.59	64.24
Aluminium	13	K series	16.50	18.73	35.17
Nickel	28	K series	0.60	0.68	0.59
		**Total**	**88.10**	**100.00**	**100.00**

Eleme

H
Li Be
Na Mg
K Ca S

Layer 4

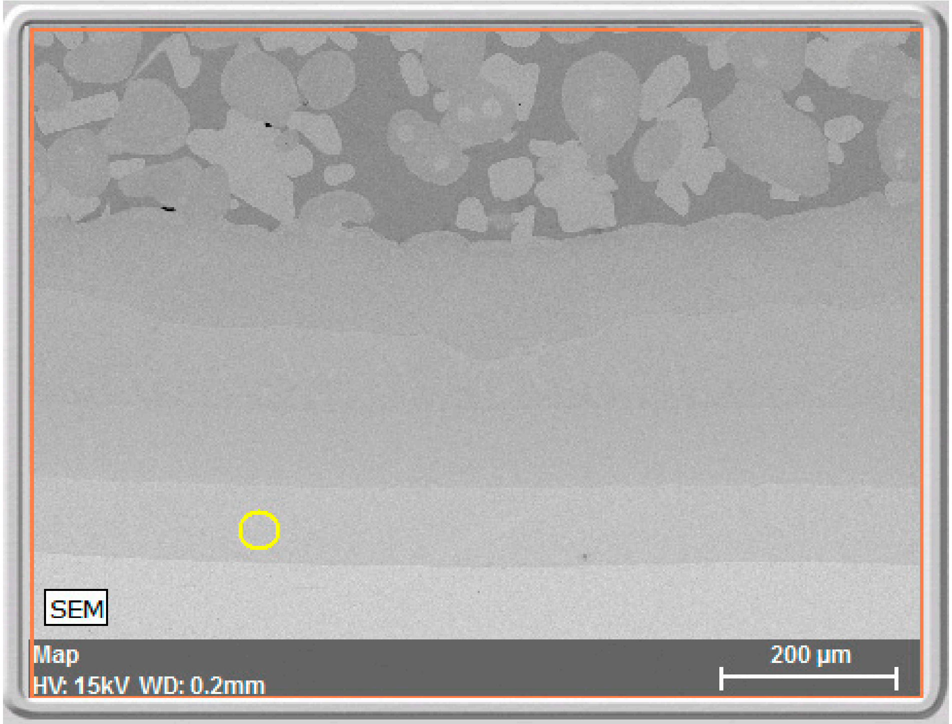

Results

Spectrum | Results | Graphic

	AN	Series	unn. C [wt.%]	nor. C [wt.%]	Atom C [at.%]	
Copper	29	K series	138.16	92.47	84.02	
Aluminium	13	K series	11.08	7.42	15.87	
Nickel	28	K series	0.17	0.11	0.11	
		**Total**	**149.41**	**100.00**	**100.00**	

Elemen

H
Li Be
Na Mg
K Ca Sc

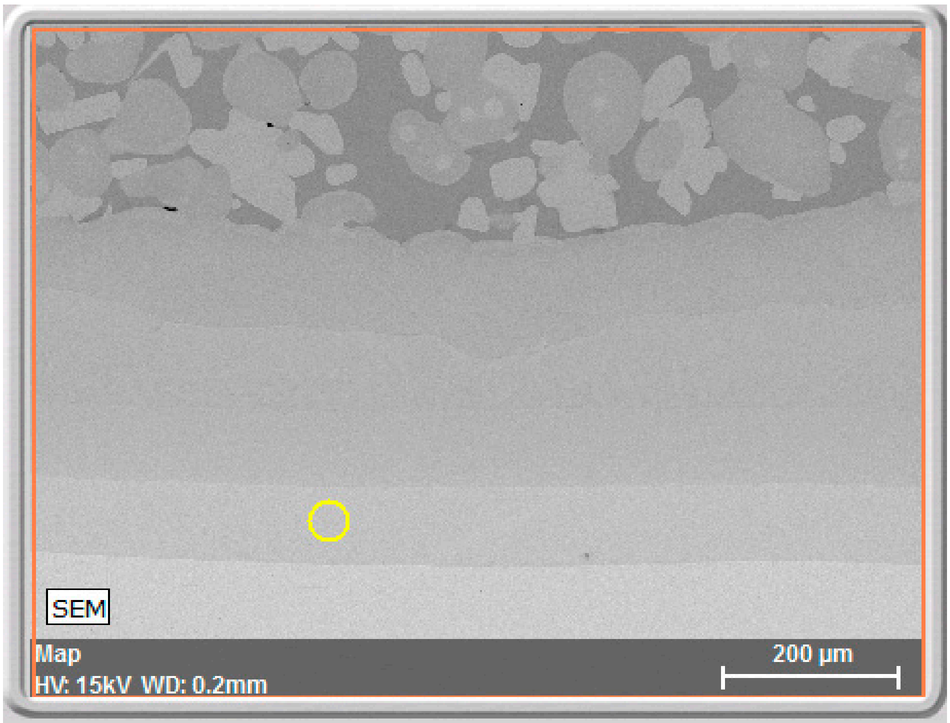

Results

Spectrum | Results | Graphic

	AN	Series	unn. C [wt.%]	nor. C [wt.%]	Atom C [at.%]
Copper	29	K series	87.25	88.33	77.36
Aluminium	13	K series	10.26	10.39	21.43
Nickel	28	K series	1.27	1.28	1.22
		**Total**	**98.77**	**100.00**	**100.00**

Eleme

H
Li Be
Na Mg
K Ca Sc

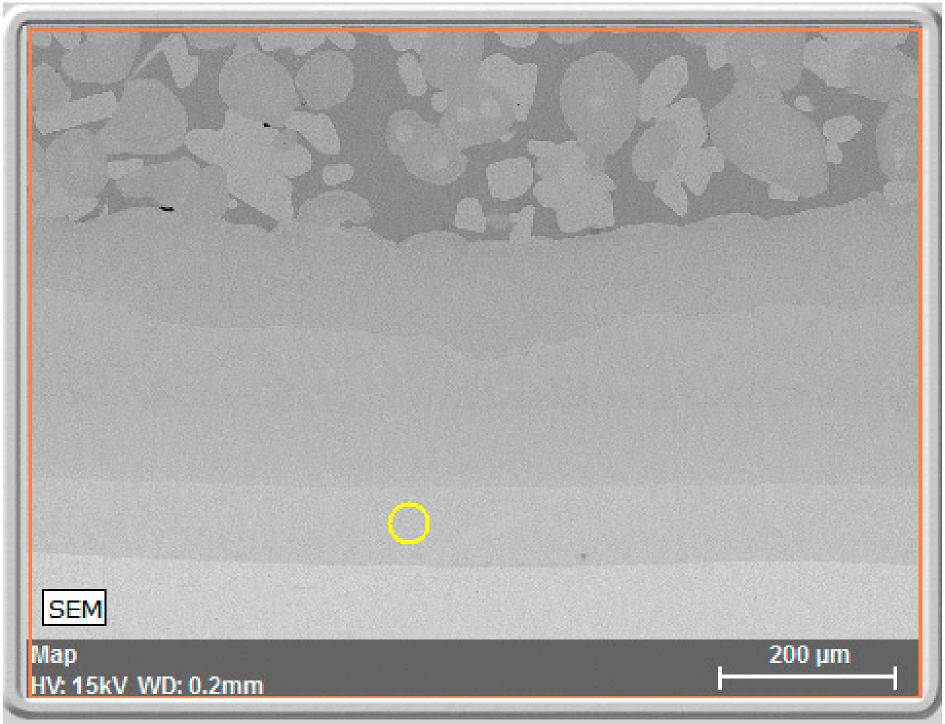

Results

Spectrum | Results | Graphic

	AN	Series	unn. C [wt.%]	nor. C [wt.%]	Atom C [at.%]
Copper	29	K series	100.13	89.40	78.99
Aluminium	13	K series	10.82	9.66	20.11
Nickel	28	K series	1.05	0.94	0.90
		**Total**	**112.00**	**100.00**	**100.00**

Eleme

H
Li Be
Na Mg
K Ca Sc

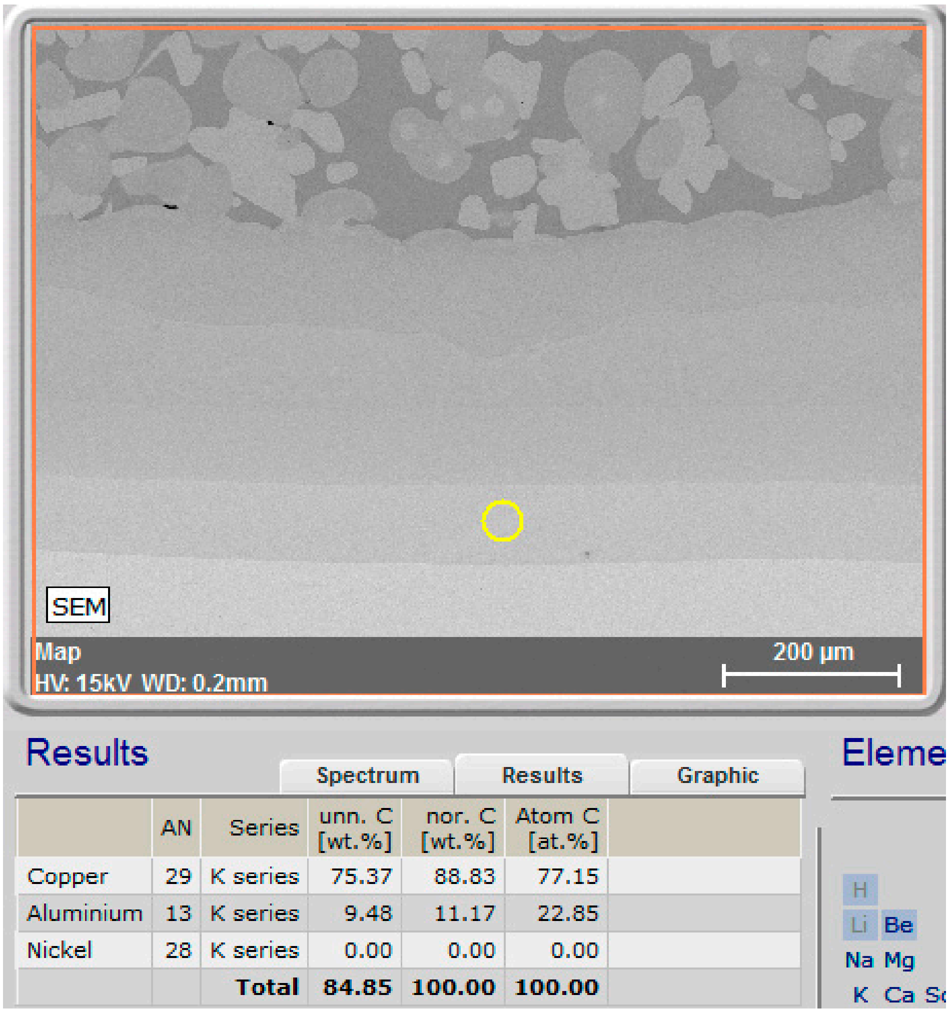

	AN	Series	unn. C [wt.%]	nor. C [wt.%]	Atom C [at.%]
Copper	29	K series	75.37	88.83	77.15
Aluminium	13	K series	9.48	11.17	22.85
Nickel	28	K series	0.00	0.00	0.00
		**Total**	**84.85**	**100.00**	**100.00**

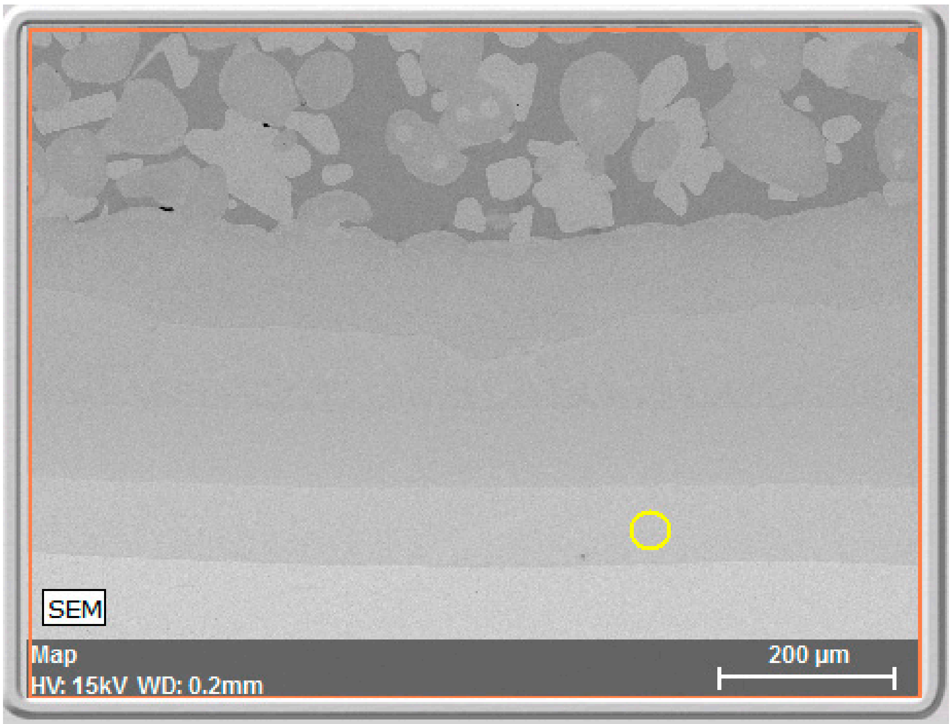

Results

Spectrum | Results | Graphic

	AN	Series	unn. C [wt.%]	nor. C [wt.%]	Atom C [at.%]
Copper	29	K series	58.81	84.23	69.50
Aluminium	13	K series	10.91	15.63	30.37
Nickel	28	K series	0.10	0.14	0.13
		**Total**	**69.82**	**100.00**	**100.00**

Elemen

H
Li Be
Na Mg
K Ca Sc

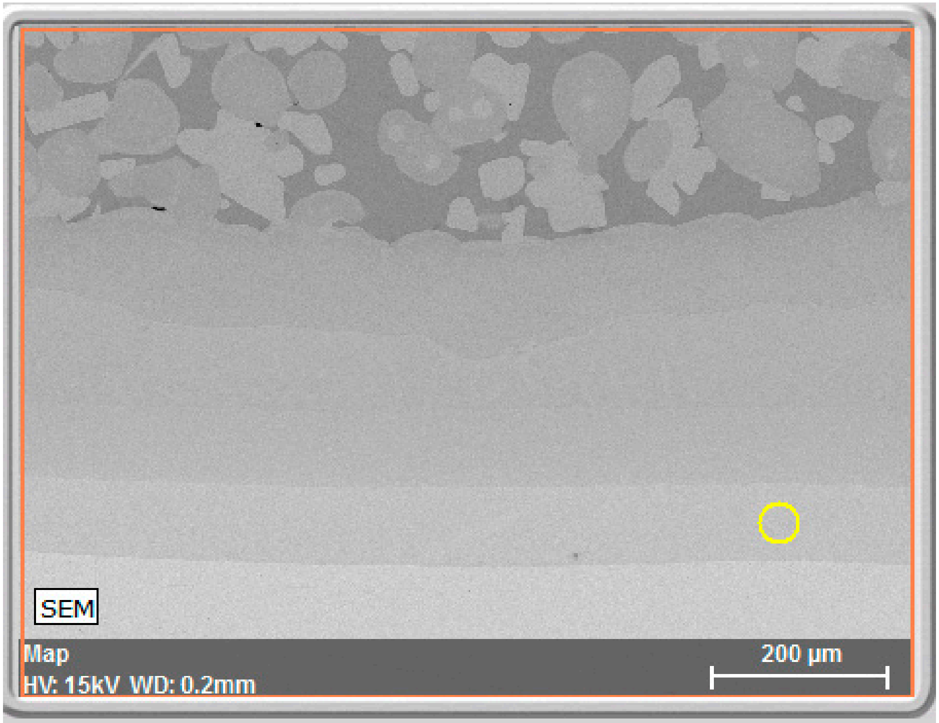

Results

Spectrum | Results | Graphic

	AN	Series	unn. C [wt.%]	nor. C [wt.%]	Atom C [at.%]
Copper	29	K series	90.92	89.32	78.09
Aluminium	13	K series	10.80	10.61	21.84
Nickel	28	K series	0.08	0.07	0.07
		**Total**	**101.79**	**100.00**	**100.00**

Eleme

H
Li Be
Na Mg
K Ca Sc

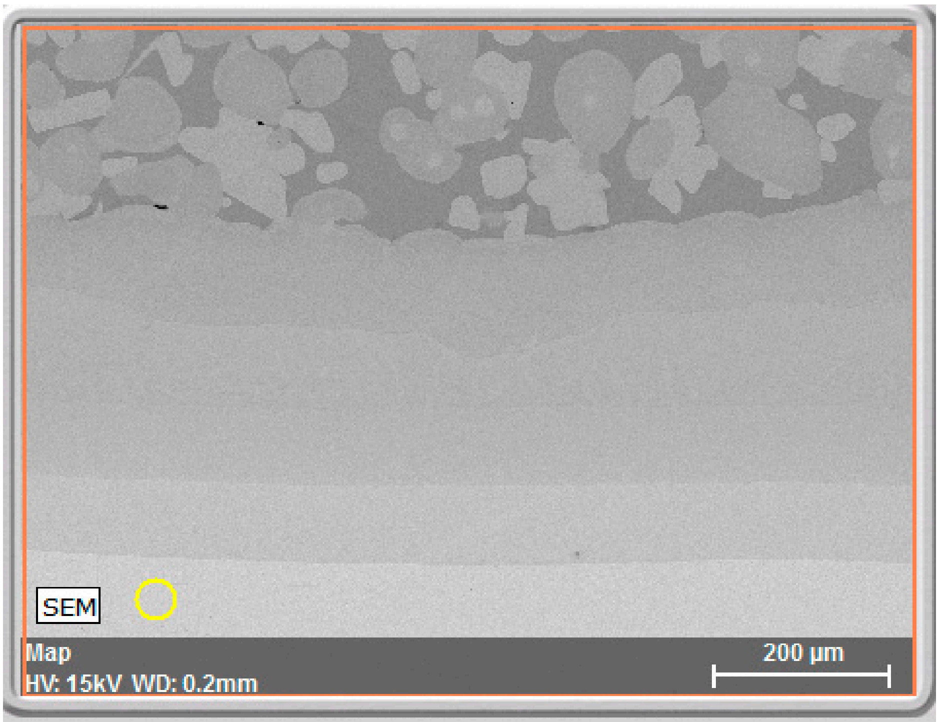

Results

Spectrum | Results | Graphic

	AN	Series	unn. C [wt.%]	nor. C [wt.%]	Atom C [at.%]
Copper	29	K series	79.55	99.63	99.32
Aluminium	13	K series	0.18	0.22	0.52
Nickel	28	K series	0.12	0.15	0.16
		**Total**	**79.84**	**100.00**	**100.00**

Eleme

H
Li Be
Na Mg
K Ca Sc

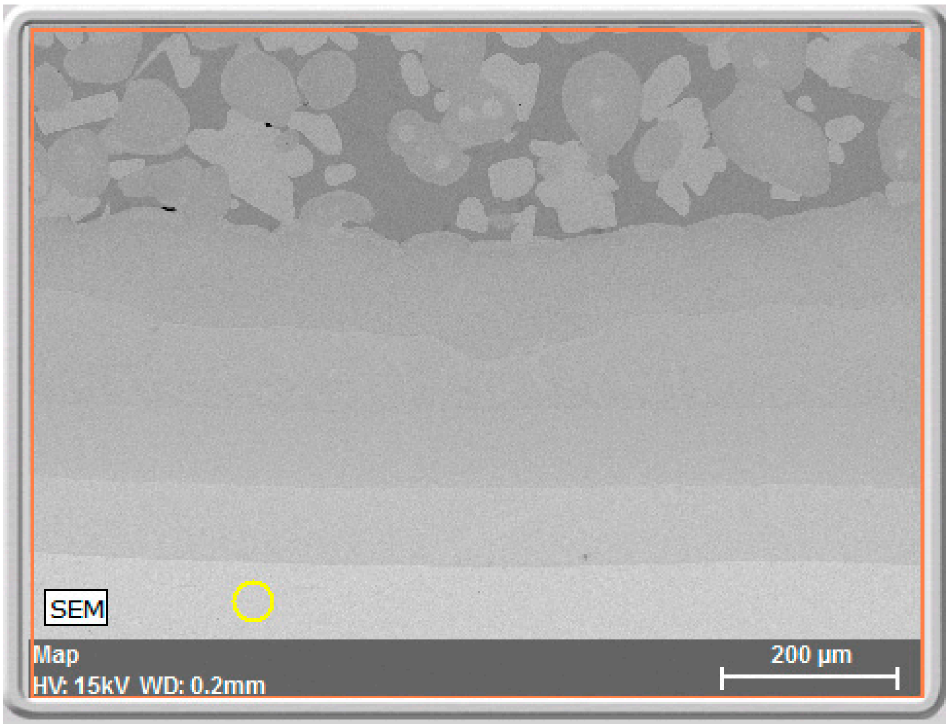

Results

Spectrum | Results | Graphic

	AN	Series	unn. C [wt.%]	nor. C [wt.%]	Atom C [at.%]	
Copper	29	K series	93.07	99.14	98.76	
Nickel	28	K series	0.57	0.61	0.66	
Aluminium	13	K series	0.23	0.25	0.58	
		**Total**	**93.88**	**100.00**	**100.00**	

Eleme

H
Li Be
Na Mg
K Ca Sc

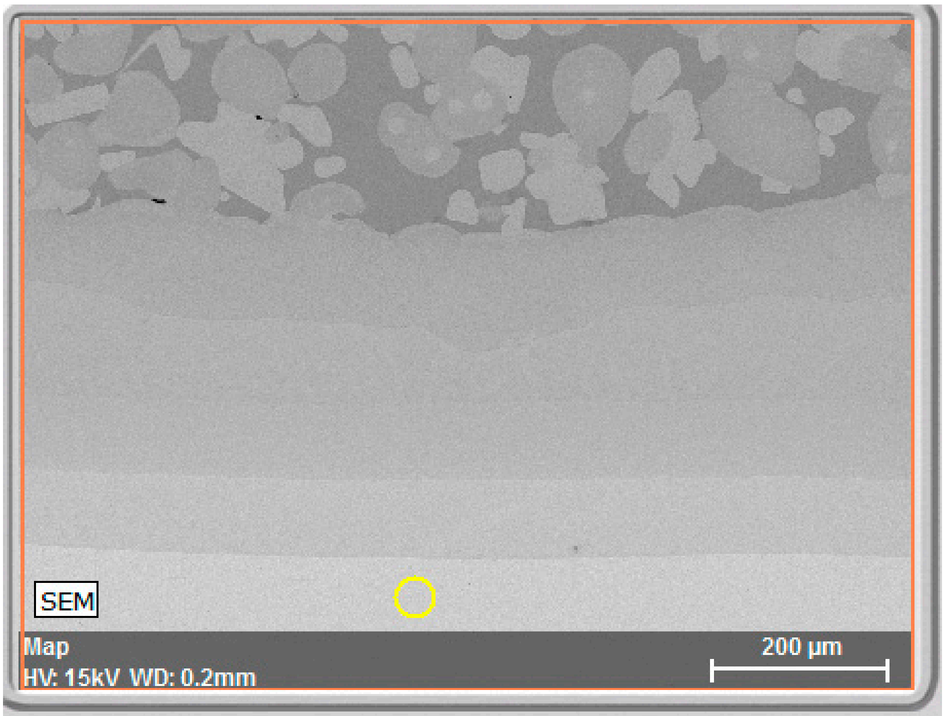

Results

Spectrum | Results | Graphic

	AN	Series	unn. C [wt.%]	nor. C [wt.%]	Atom C [at.%]
Copper	29	K series	87.11	99.42	98.64
Aluminium	13	K series	0.51	0.58	1.36
Nickel	28	K series	0.00	0.00	0.00
		**Total**	**87.62**	**100.00**	**100.00**

Elemen

H
Li Be
Na Mg
K Ca Sc

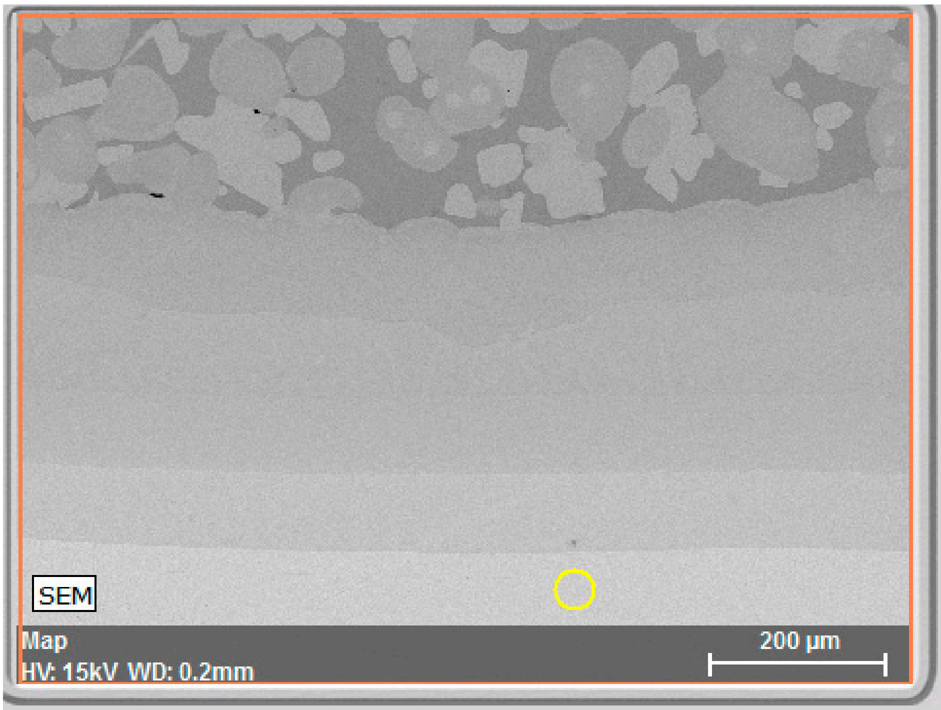

Results

Spectrum | Results | Graphic

	AN	Series	unn. C [wt.%]	nor. C [wt.%]	Atom C [at.%]
Copper	29	K series	98.19	98.84	98.69
Nickel	28	K series	1.10	1.11	1.20
Aluminium	13	K series	0.05	0.05	0.11
		**Total**	**99.34**	**100.00**	**100.00**

Elemen

H
Li Be
Na Mg
K Ca Sc

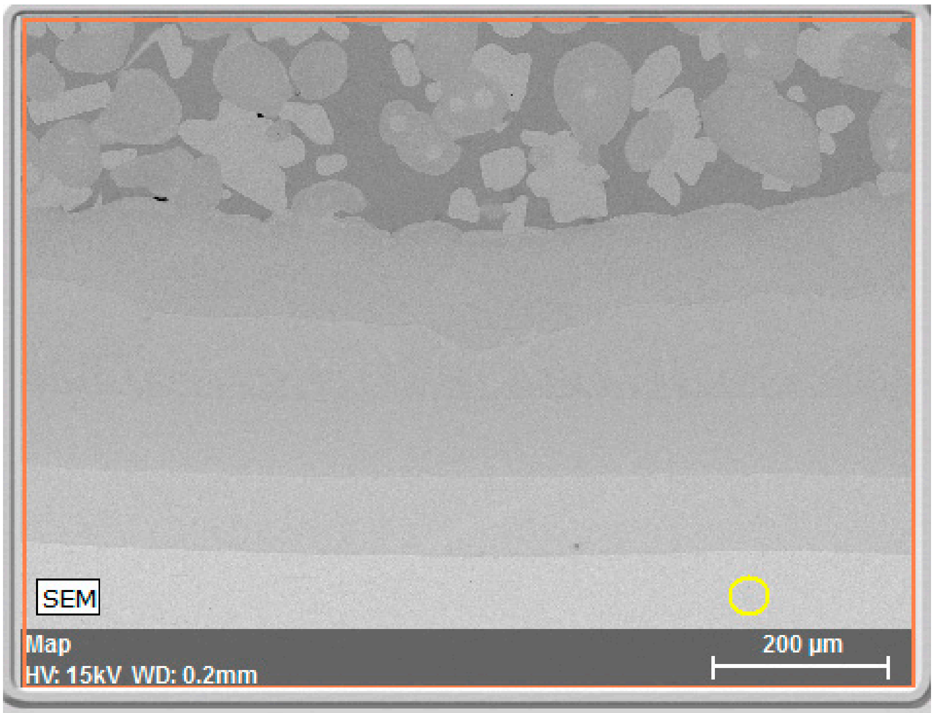

Results

Spectrum | Results | Graphic

	AN	Series	unn. C [wt.%]	nor. C [wt.%]	Atom C [at.%]
Copper	29	K series	96.31	98.64	98.12
Nickel	28	K series	1.00	1.03	1.10
Aluminium	13	K series	0.32	0.33	0.78
		**Total**	**97.64**	**100.00**	**100.00**

Eleme

H
Li Be
Na Mg
K Ca S
