# Supplementary material for: Effect of Ni Addition on the Solidification of Liquid Al and Solid Cu Diffusion Couples
Source: Materials (Basel). 2025 Dec 18;18(24):5689. doi: 10.3390/ma18245689 (PMC12735080; doi:10.3390/ma18245689)
Supplement: Supplementary file 1 [file materials-18-05689-s001.zip › Supplementary Figures/Files S1/Figure 6/Figure 6 EDS.pdf]

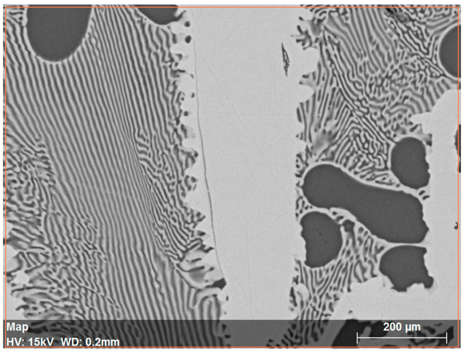

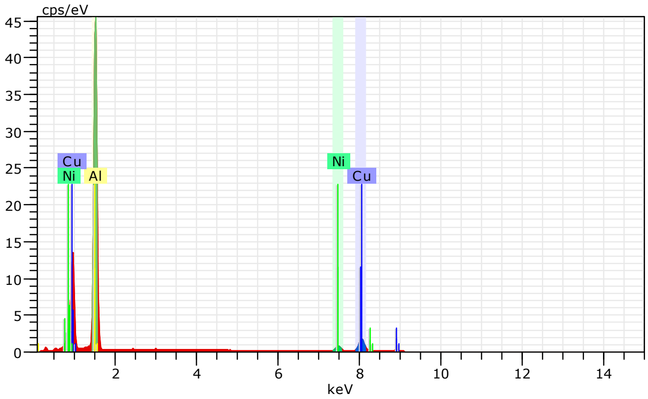

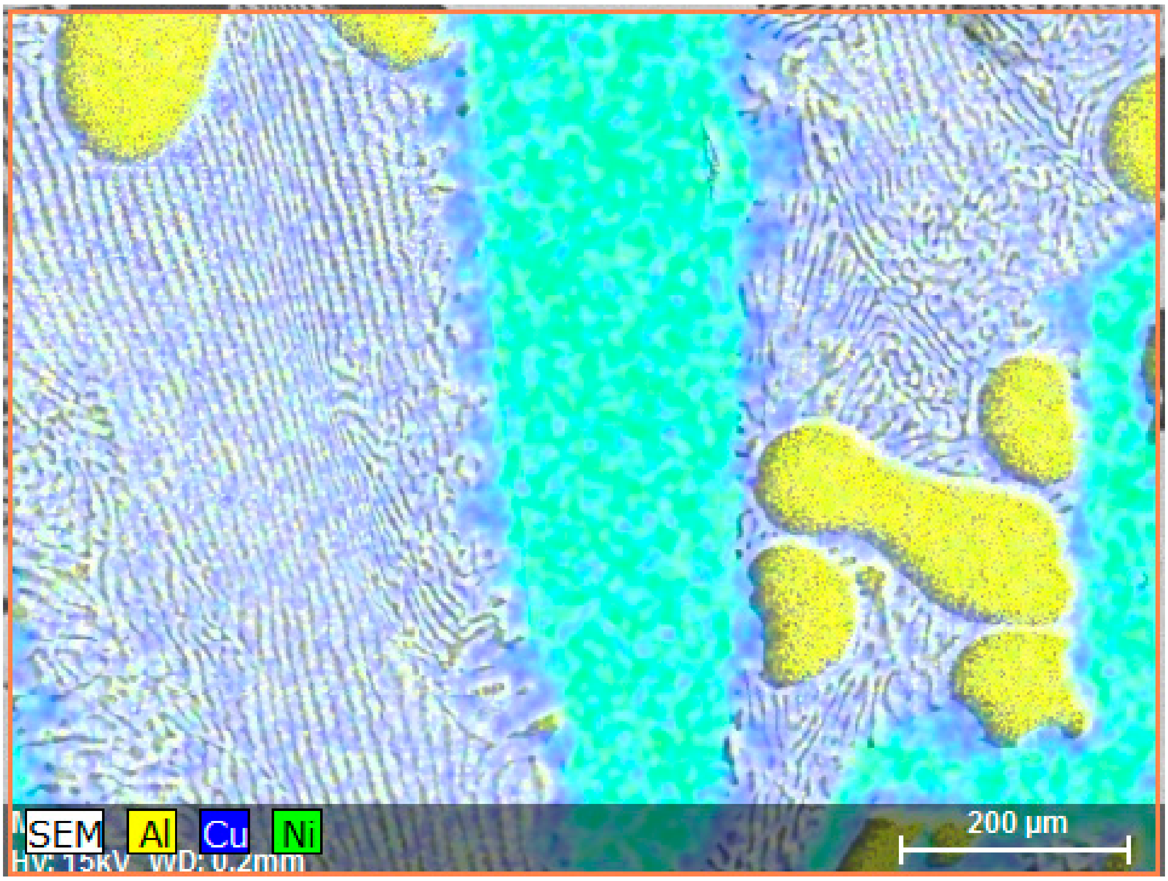

MapDate:15-Sep-25 5:21:15 PMImage size:480 x 360

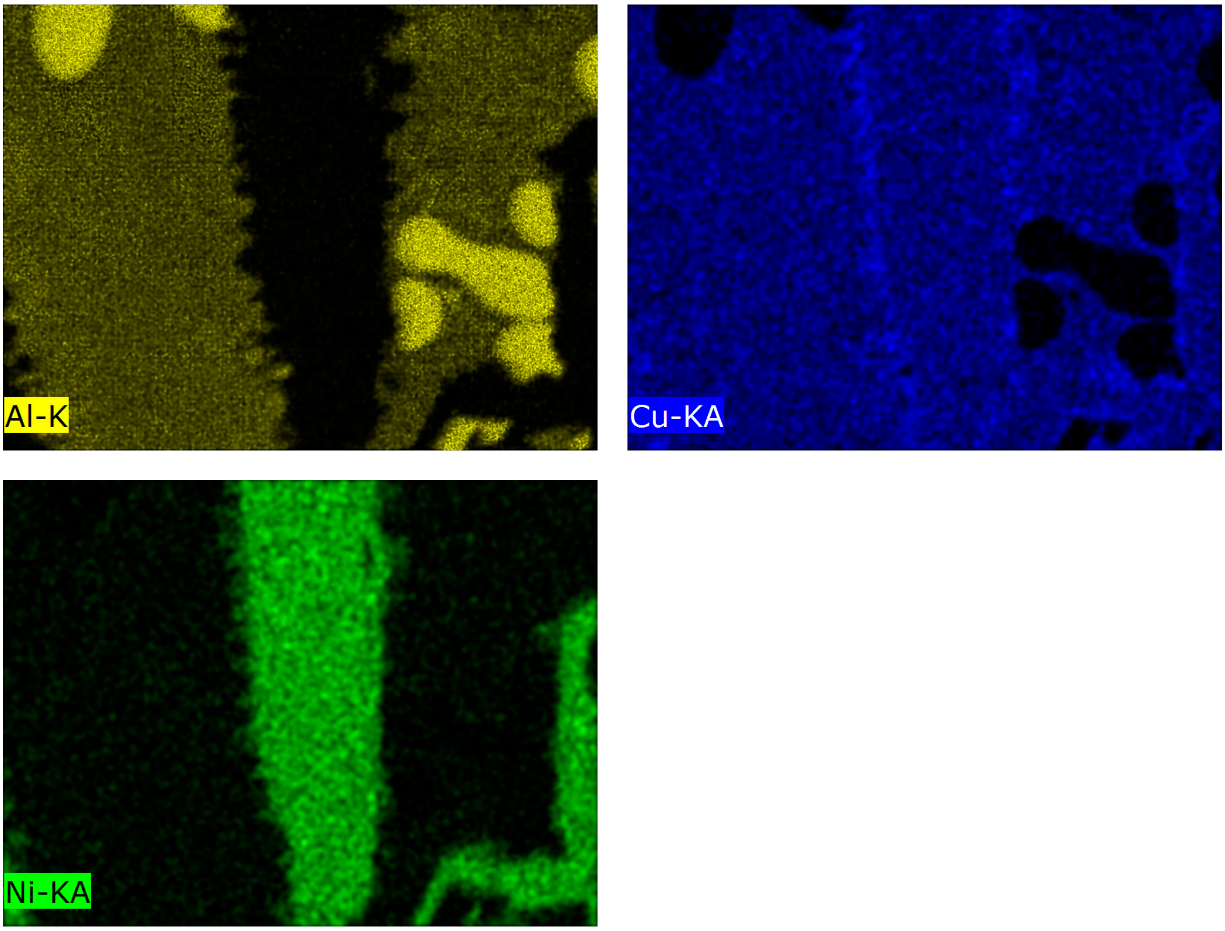
Al-K
Cu-KA
Ni-KA

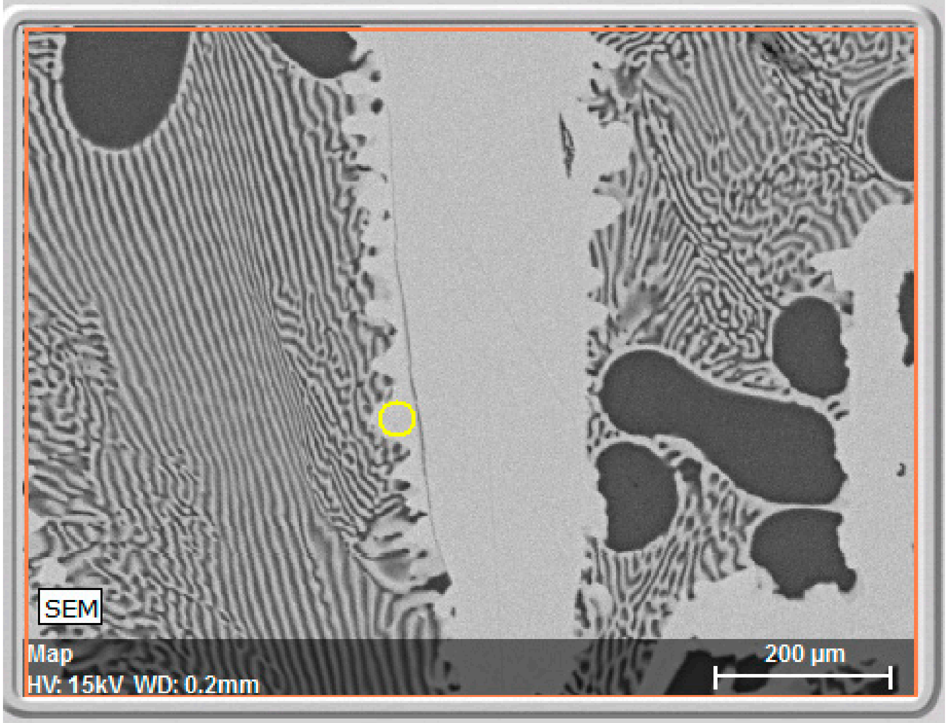

Results

Spectrum | Results | Graphic

	AN	Series	unn. C [wt.%]	nor. C [wt.%]	Atom C [at.%]
Aluminium	13	K series	36.09	47.52	67.70
Copper	29	K series	31.44	41.39	25.04
Nickel	28	K series	8.42	11.09	7.26
		Total	75.95	100.00	100.00

Elemen

H
Li Be
Na Mg
K Ca Sc

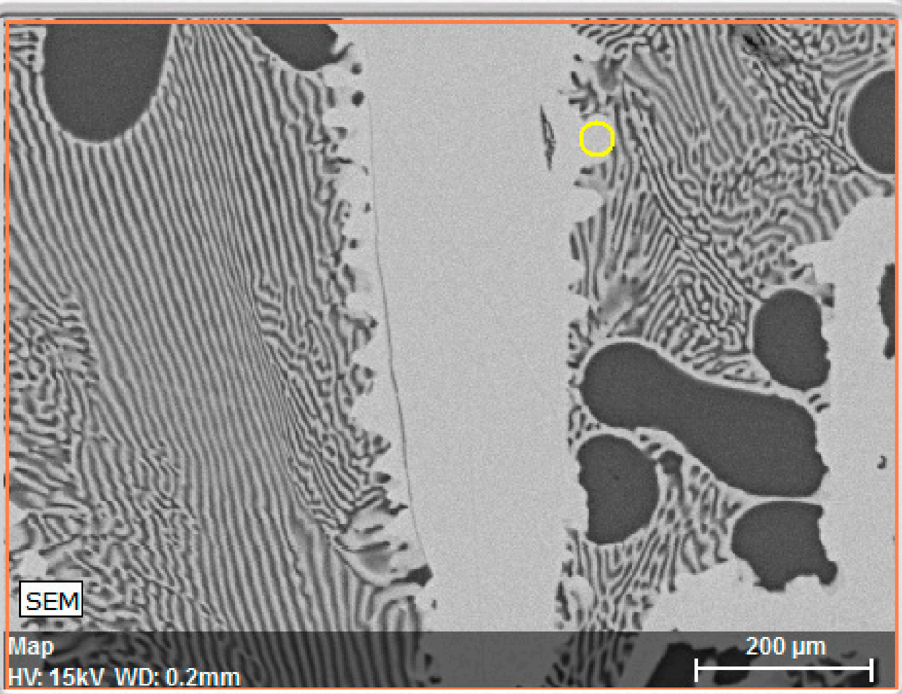

Results

Spectrum | Results | Graphic

	AN	Series	unn. C [wt.%]	nor. C [wt.%]	Atom C [at.%]
Aluminium	13	K series	37.06	40.27	60.97
Copper	29	K series	43.88	47.69	30.65
Nickel	28	K series	11.08	12.04	8.38
		**Total**	**92.02**	**100.00**	**100.00**

Eleme

H
Li Be
Na Mg
K Ca Sc

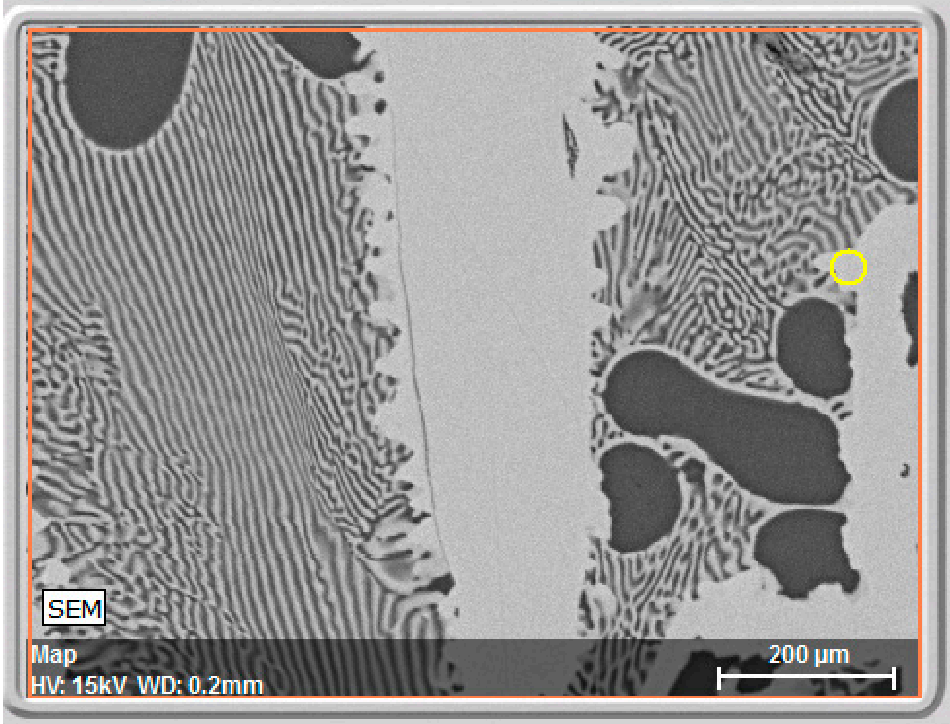

Results

Spectrum | Results | Graphic

	AN	Series	unn. C [wt.%]	nor. C [wt.%]	Atom C [at.%]
Aluminium	13	K series	47.47	40.27	61.04
Copper	29	K series	58.94	50.00	32.18
Nickel	28	K series	11.46	9.72	6.78
		**Total**	**117.88**	**100.00**	**100.00**

Elemer

H
Li Be
Na Mg
K Ca Sc

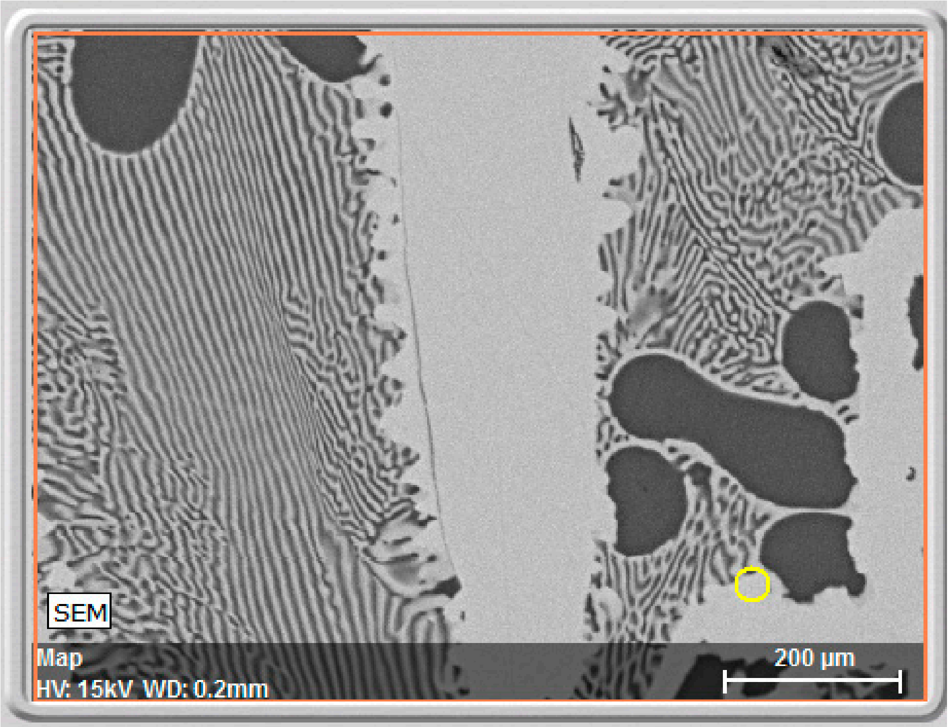

Results

Spectrum | Results | Graphic

	AN	Series	unn. C [wt.%]	nor. C [wt.%]	Atom C [at.%]
Aluminium	13	K series	39.89	37.86	58.49
Copper	29	K series	51.13	48.52	31.83
Nickel	28	K series	14.36	13.63	9.68
		**Total**	**105.38**	**100.00**	**100.00**

Eleme

H
Li Be
Na Mg
K Ca Sc

Al_3Ni_2

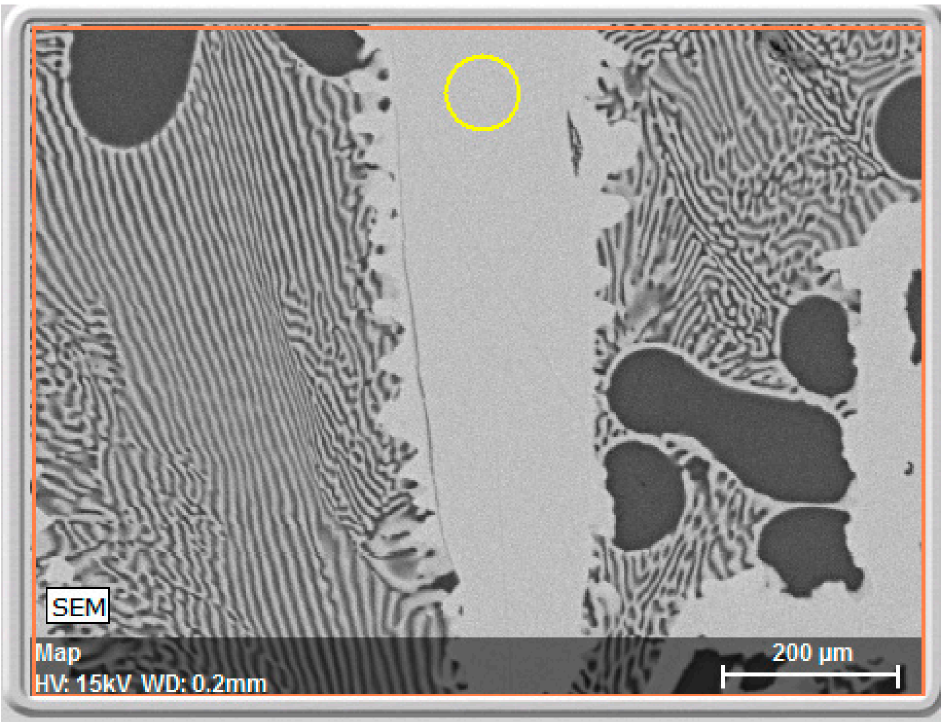

Results

Spectrum | Results | Graphic

	AN	Series	unn. C [wt.%]	nor. C [wt.%]	Atom C [at.%]
Aluminium	13	K series	36.68	42.28	62.36
Nickel	28	K series	25.10	28.94	19.62
Copper	29	K series	24.97	28.78	18.02
		**Total**	**86.75**	**100.00**	**100.00**

Eleme

H
Li Be
Na Mg
K Ca S

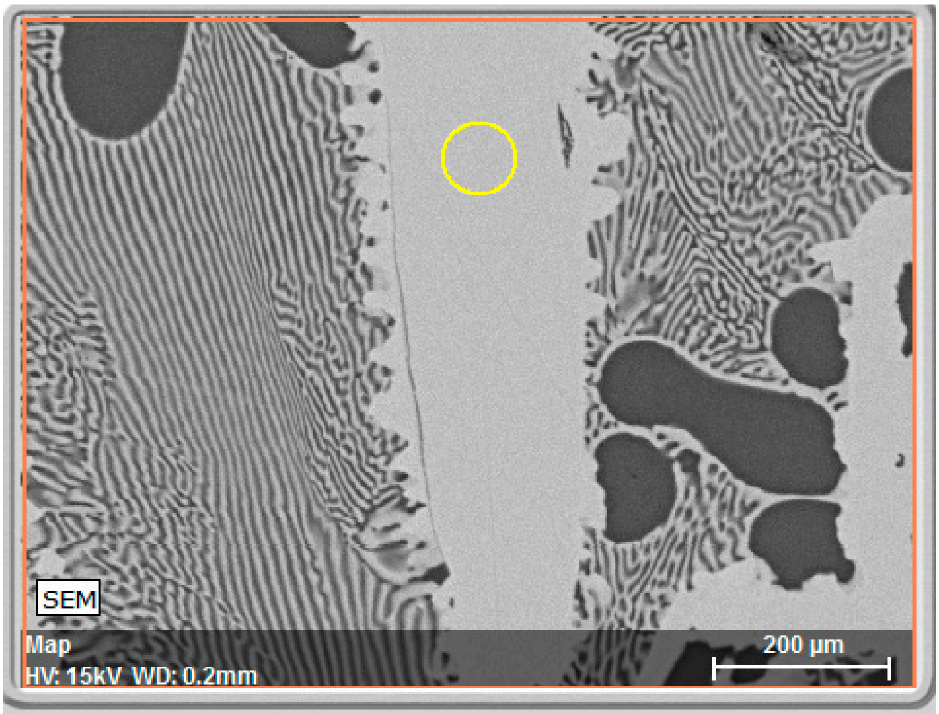

Results

Spectrum | Results | Graphic

	AN	Series	unn. C [wt.%]	nor. C [wt.%]	Atom C [at.%]
Aluminium	13	K series	33.27	47.37	66.90
Nickel	28	K series	21.82	31.08	20.18
Copper	29	K series	15.13	21.55	12.92
		**Total**	**70.22**	**100.00**	**100.00**

Eleme

H
Li Be
Na Mg
K Ca Sc

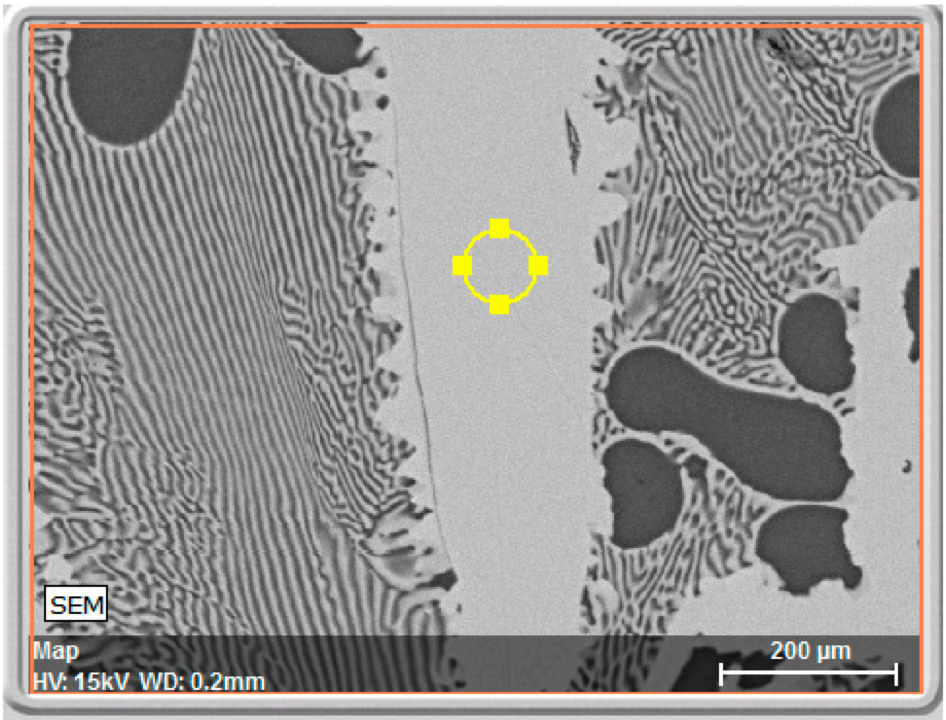

Results

Spectrum | Results | Graphic

	AN	Series	unn. C [wt.%]	nor. C [wt.%]	Atom C [at.%]
Aluminium	13	K series	34.54	45.31	65.13
Nickel	28	K series	22.43	29.43	19.45
Copper	29	K series	19.26	25.26	15.42
		**Total**	**76.23**	**100.00**	**100.00**

Eleme

H
Li Be
Na Mg
K Ca Sc

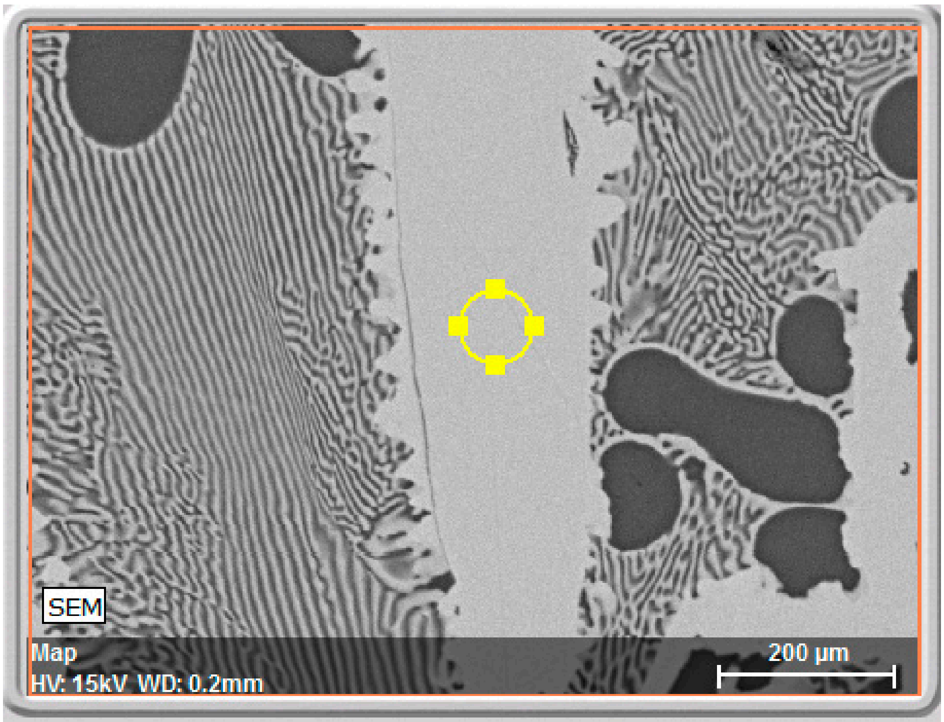

Results

Spectrum | Results | Graphic

	AN	Series	unn. C [wt.%]	nor. C [wt.%]	Atom C [at.%]
Aluminium	13	K series	34.03	50.57	69.69
Nickel	28	K series	19.38	28.80	18.25
Copper	29	K series	13.88	20.63	12.07
		**Total**	**67.30**	**100.00**	**100.00**

Eleme

H
Li Be
Na Mg
K Ca Sc

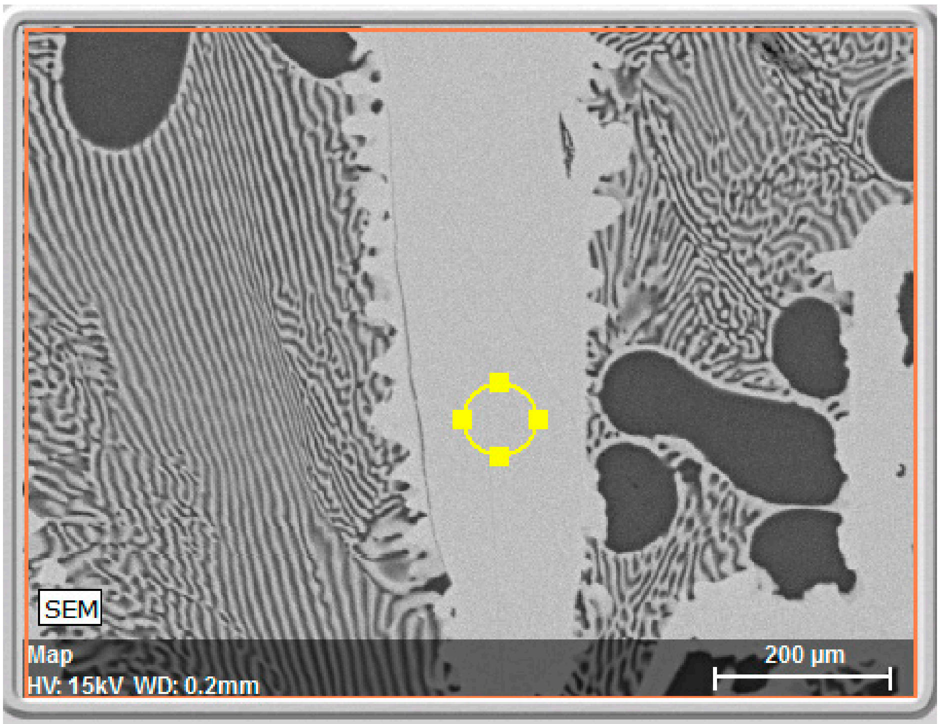

Results

Spectrum | Results | Graphic

	AN	Series	unn. C [wt.%]	nor. C [wt.%]	Atom C [at.%]
Aluminium	13	K series	34.04	46.48	66.21
Nickel	28	K series	20.85	28.47	18.64
Copper	29	K series	18.35	25.06	15.15
		**Total**	**73.25**	**100.00**	**100.00**

Eleme

H
Li Be
Na Mg
K Ca Sc

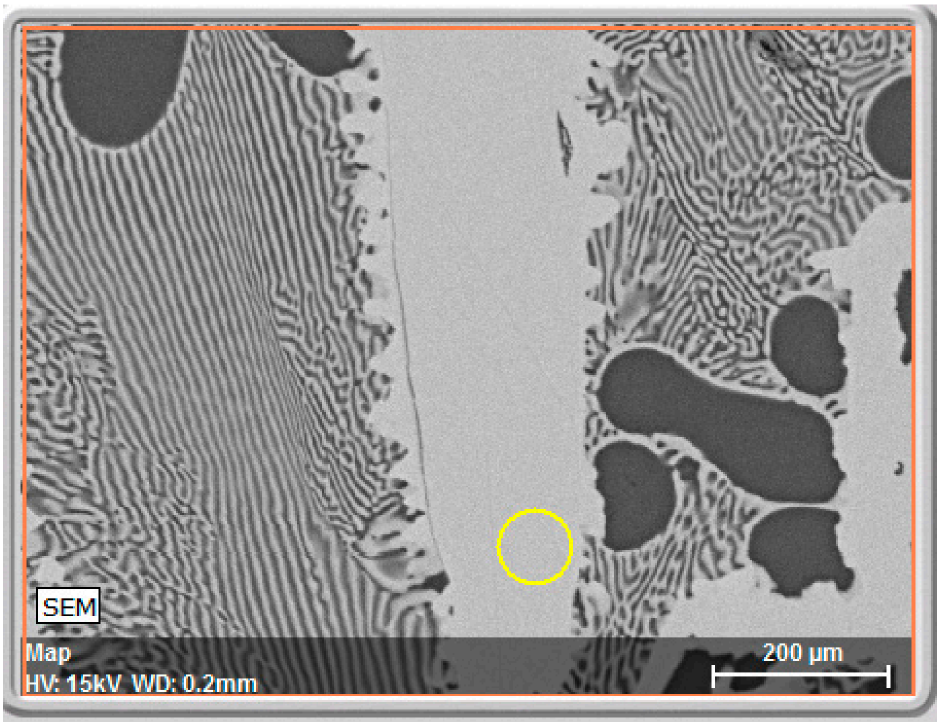

Results

Spectrum | Results | Graphic

	AN	Series	unn. C [wt.%]	nor. C [wt.%]	Atom C [at.%]
Aluminium	13	K series	35.48	47.09	66.80
Nickel	28	K series	20.18	26.78	17.46
Copper	29	K series	19.68	26.12	15.73
		**Total**	**75.35**	**100.00**	**100.00**

Eleme

H
Li Be
Na Mg
K Ca Sc

Eutectic Al-Al2Cu

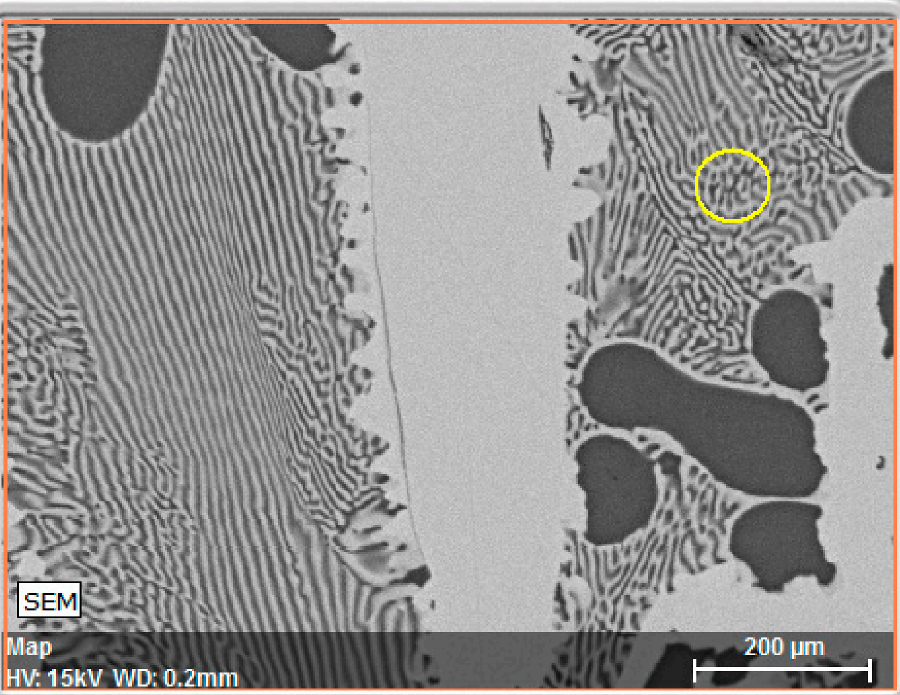

Results

Spectrum | Results | Graphic

	AN	Series	unn. C [wt.%]	nor. C [wt.%]	Atom C [at.%]	
Aluminium	13	K series	68.08	68.32	83.52	
Copper	29	K series	30.71	30.82	16.00	
Nickel	28	K series	0.86	0.86	0.49	
		**Total**	**99.65**	**100.00**	**100.00**	

Eleme

H
Li Be
Na Mg
K Ca Sc

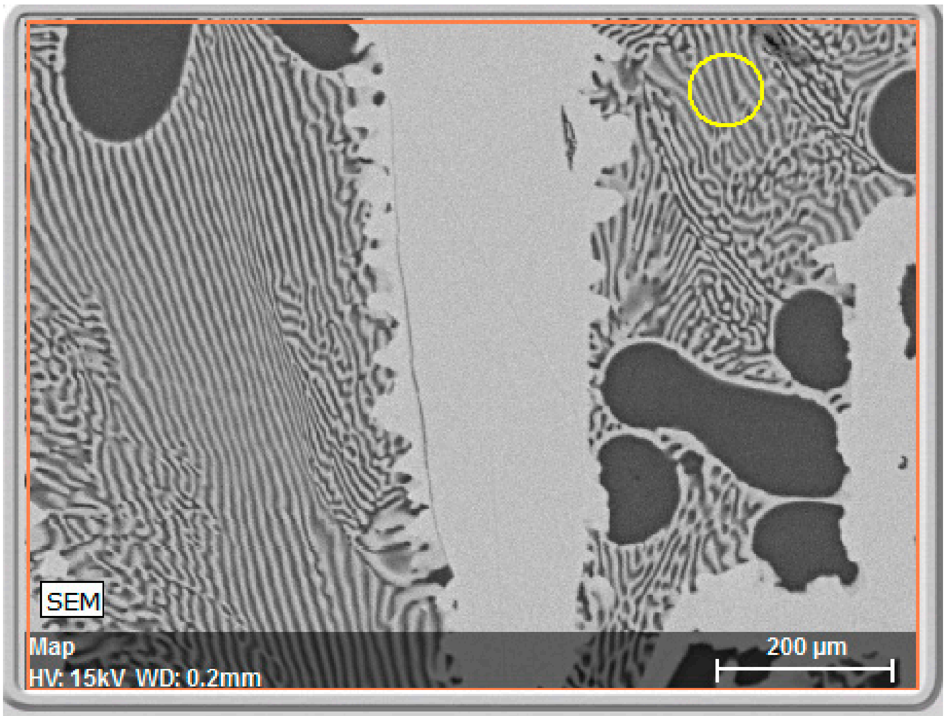

Results

Spectrum | Results | Graphic

	AN	Series	unn. C [wt.%]	nor. C [wt.%]	Atom C [at.%]	
Aluminium	13	K series	69.60	71.08	85.24	
Copper	29	K series	27.54	28.12	14.32	
Nickel	28	K series	0.78	0.80	0.44	
		**Total**	**97.91**	**100.00**	**100.00**	

Eleme

H
Li Be
Na Mg
K Ca Sc

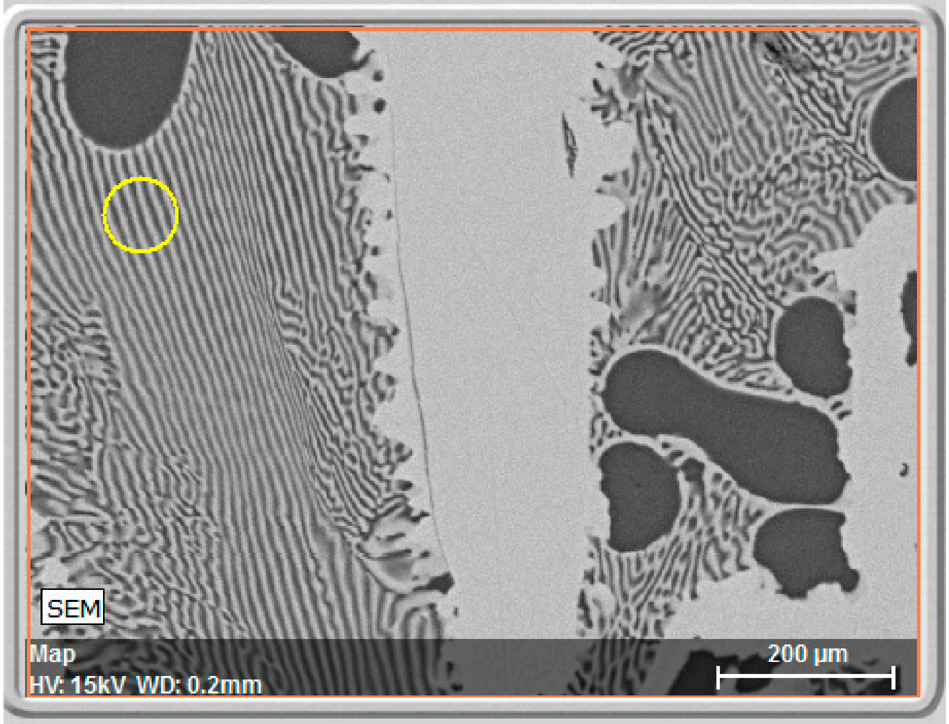

Results

Spectrum | Results | Graphic

	AN	Series	unn. C [wt.%]	nor. C [wt.%]	Atom C [at.%]
Aluminium	13	K series	63.61	70.97	85.18
Copper	29	K series	25.34	28.27	14.40
Nickel	28	K series	0.68	0.76	0.42
		**Total**	**89.63**	**100.00**	**100.00**

Elemen

H
Li Be
Na Mg
K Ca Sc

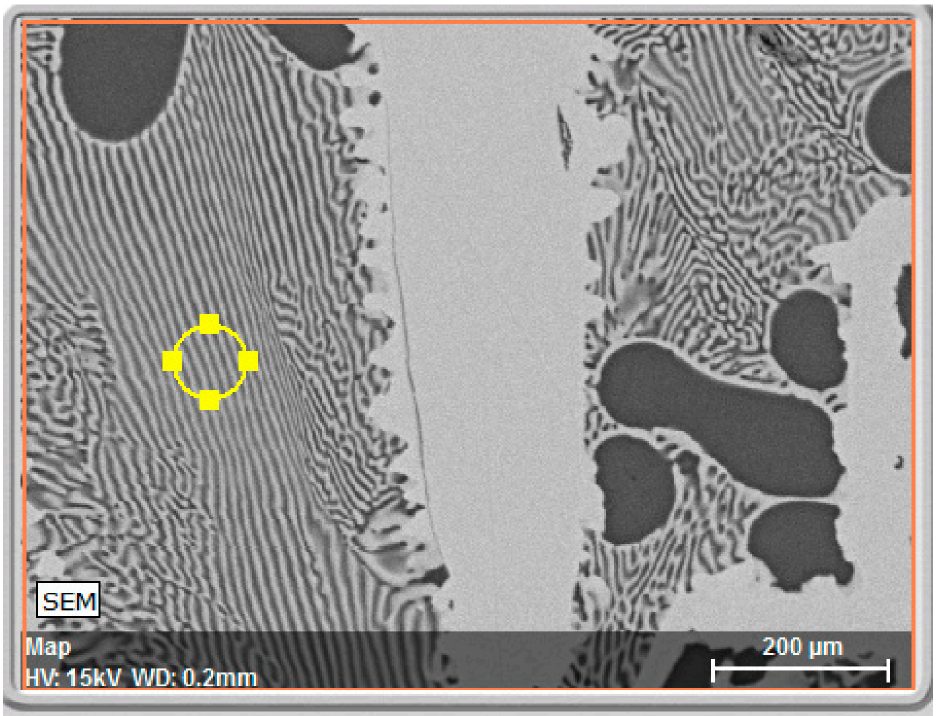

Results

Spectrum | Results | Graphic

	AN	Series	unn. C [wt.%]	nor. C [wt.%]	Atom C [at.%]
Aluminium	13	K series	66.06	72.70	86.23
Copper	29	K series	24.30	26.74	13.47
Nickel	28	K series	0.51	0.56	0.31
		**Total**	**90.87**	**100.00**	**100.00**

Eleme

H
Li Be
Na Mg
K Ca S

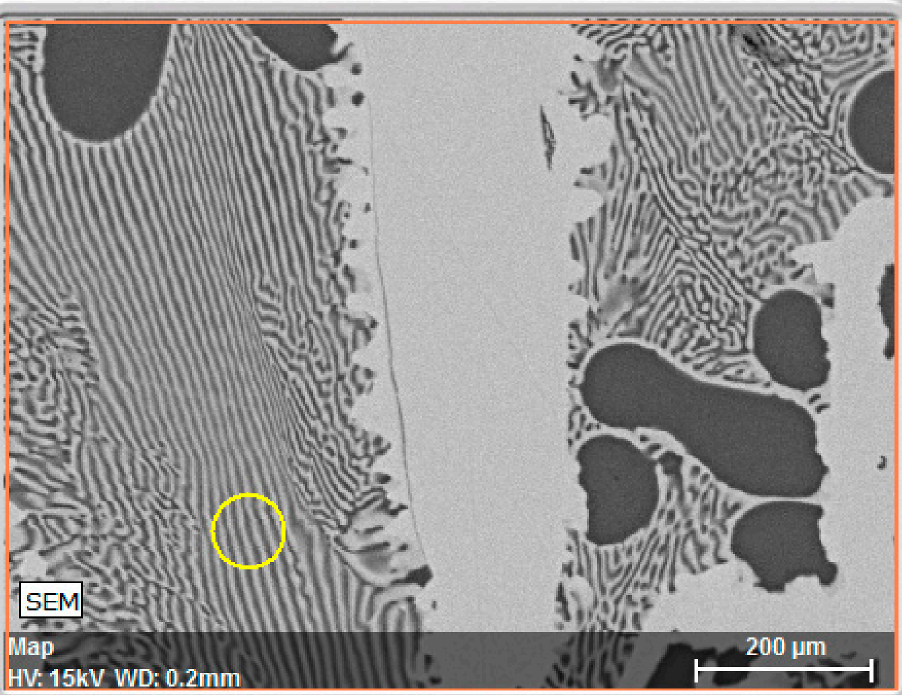

Results

Spectrum | Results | Graphic

	AN	Series	unn. C [wt.%]	nor. C [wt.%]	Atom C [at.%]
Aluminium	13	K series	66.32	71.24	85.34
Copper	29	K series	26.04	27.97	14.23
Nickel	28	K series	0.73	0.79	0.43
		**Total**	**93.09**	**100.00**	**100.00**

Eleme

H
Li Be
Na Mg
K Ca Sc
