# Supplementary figures and images for: Effect of Ni Addition on the Solidification of Liquid Al and Solid Cu Diffusion Couples
Source: Materials (Basel). 2025 Dec 18;18(24):5689. doi: 10.3390/ma18245689 (PMC12735080; doi:10.3390/ma18245689)

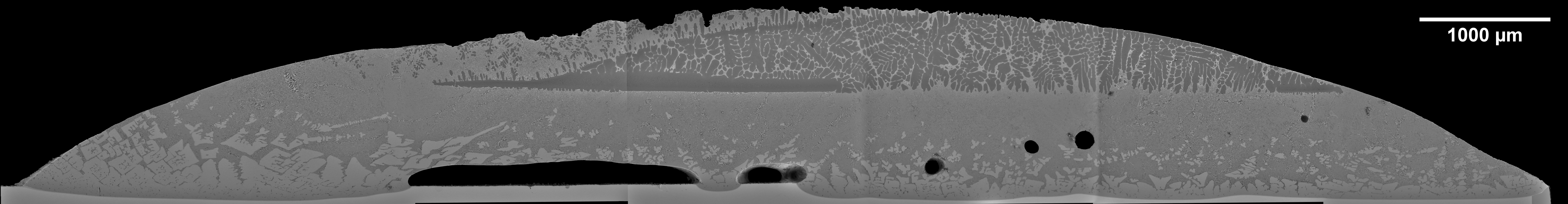

Supplement: Supplementary file 1 [file materials-18-05689-s001.zip › Supplementary Figures/Figures S1/Al 15s.tif]

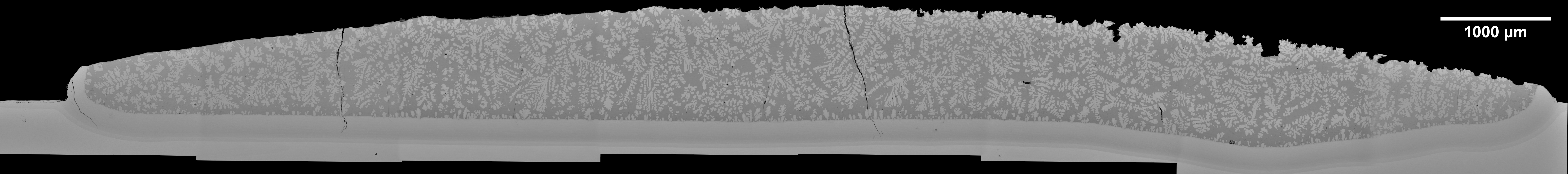

Supplement: Supplementary file 1 [file materials-18-05689-s001.zip › Supplementary Figures/Figures S1/Al 1800s.tif]

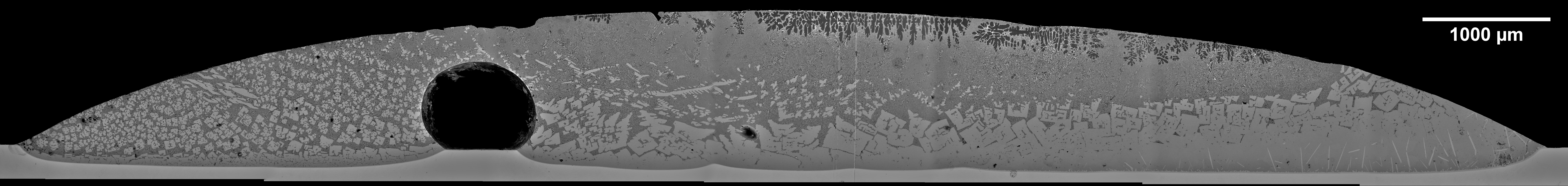

Supplement: Supplementary file 1 [file materials-18-05689-s001.zip › Supplementary Figures/Figures S1/Al 30s.tif]

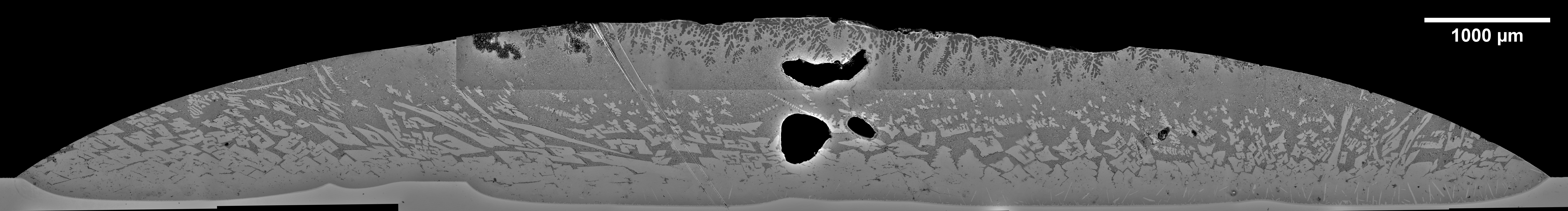

Supplement: Supplementary file 1 [file materials-18-05689-s001.zip › Supplementary Figures/Figures S1/Al 60s.tif]

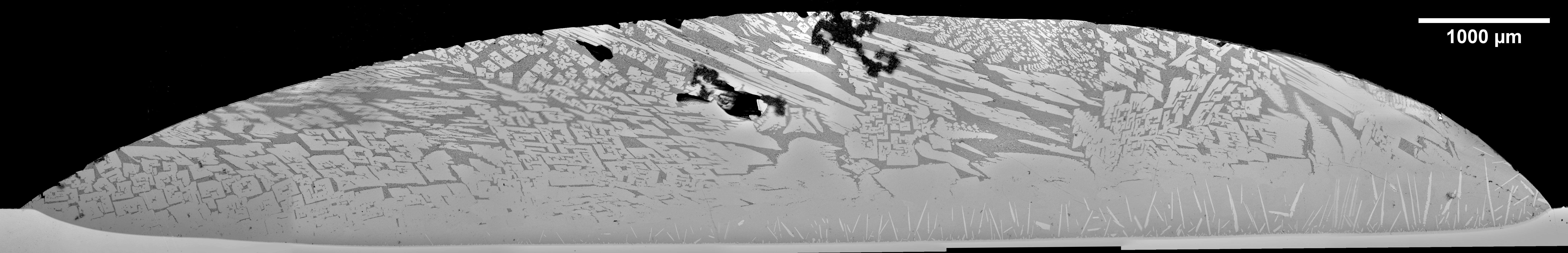

Supplement: Supplementary file 1 [file materials-18-05689-s001.zip › Supplementary Figures/Figures S1/Al 90s.tif]

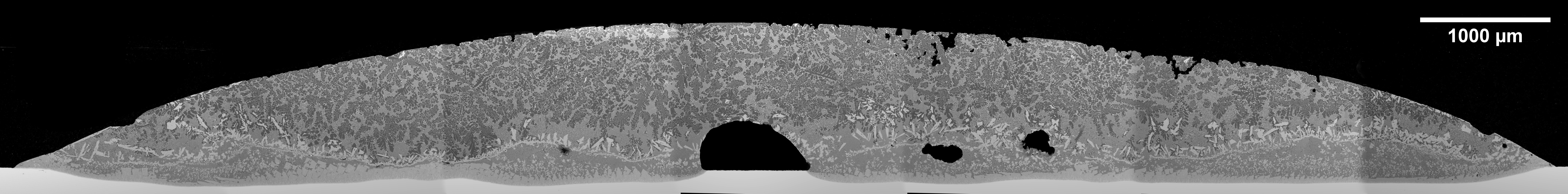

Supplement: Supplementary file 1 [file materials-18-05689-s001.zip › Supplementary Figures/Figures S1/Al-Ni 15s.tif]

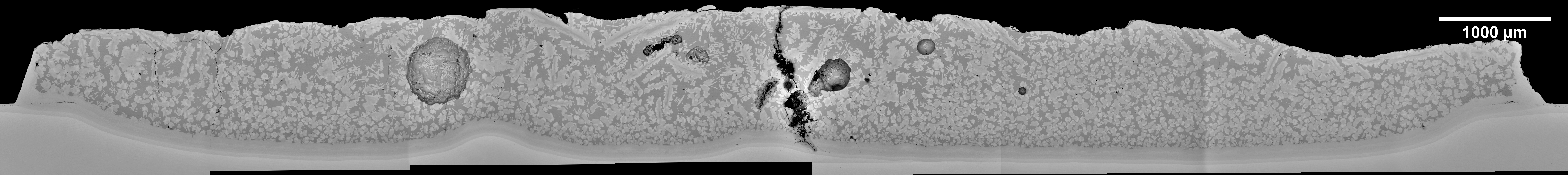

Supplement: Supplementary file 1 [file materials-18-05689-s001.zip › Supplementary Figures/Figures S1/Al-Ni 1800s.tif]

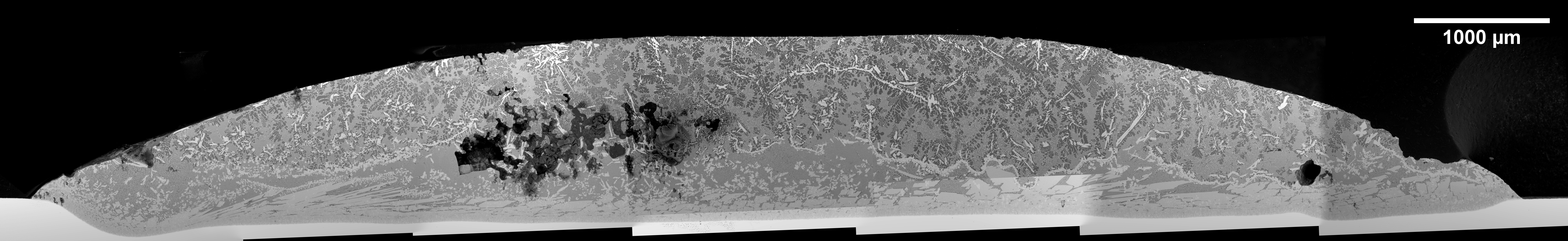

Supplement: Supplementary file 1 [file materials-18-05689-s001.zip › Supplementary Figures/Figures S1/Al-Ni 30s 2.tif]

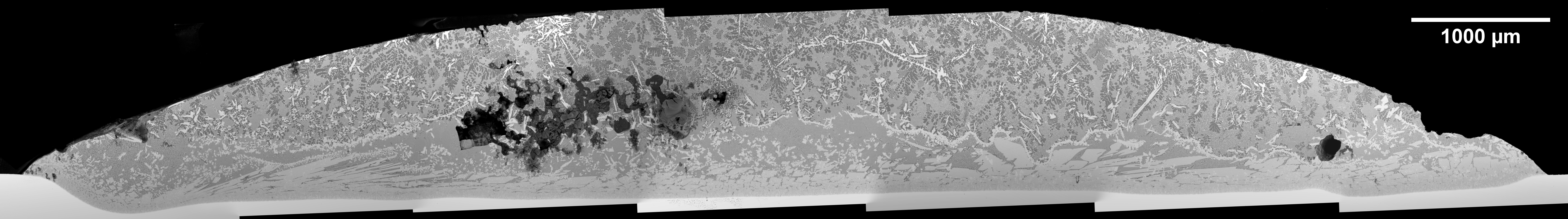

Supplement: Supplementary file 1 [file materials-18-05689-s001.zip › Supplementary Figures/Figures S1/Al-Ni 30s.tif]

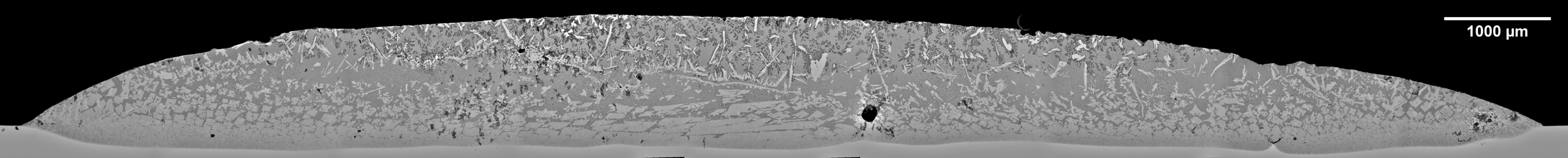

Supplement: Supplementary file 1 [file materials-18-05689-s001.zip › Supplementary Figures/Figures S1/Al-Ni 60s.tif]

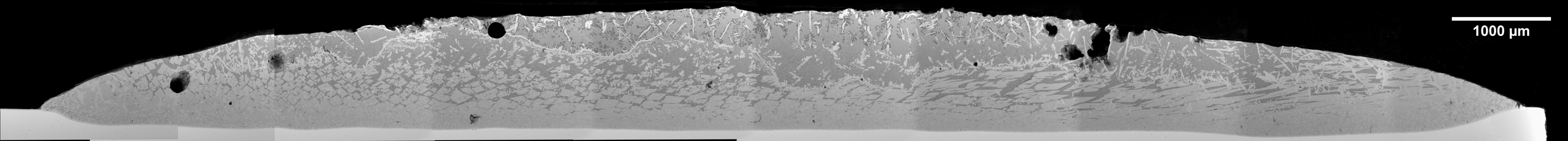

Supplement: Supplementary file 1 [file materials-18-05689-s001.zip › Supplementary Figures/Figures S1/Al-Ni 90s.tif]

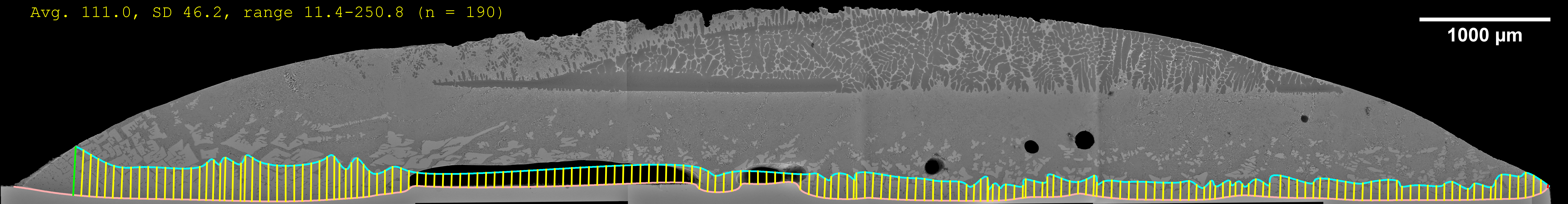

Supplement: Supplementary file 1 [file materials-18-05689-s001.zip › Supplementary Figures/Figures S2/Al 15s.tif]

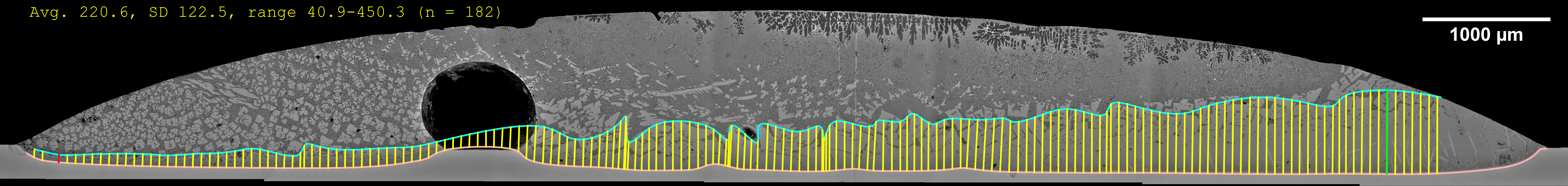

Supplement: Supplementary file 1 [file materials-18-05689-s001.zip › Supplementary Figures/Figures S2/Al 30s.tif]

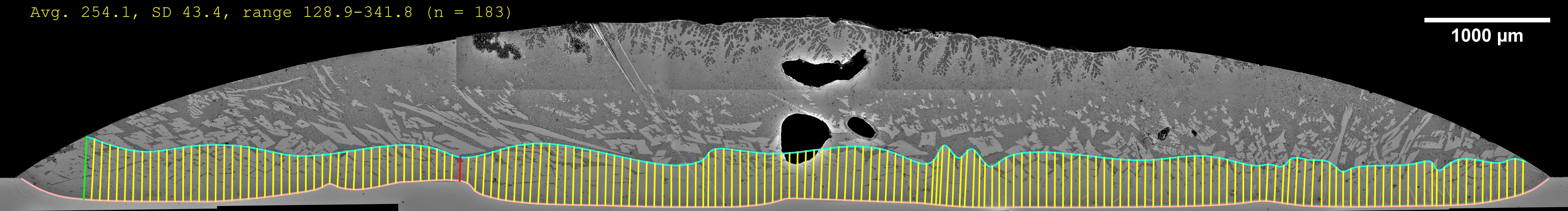

Supplement: Supplementary file 1 [file materials-18-05689-s001.zip › Supplementary Figures/Figures S2/Al 60s.tif]

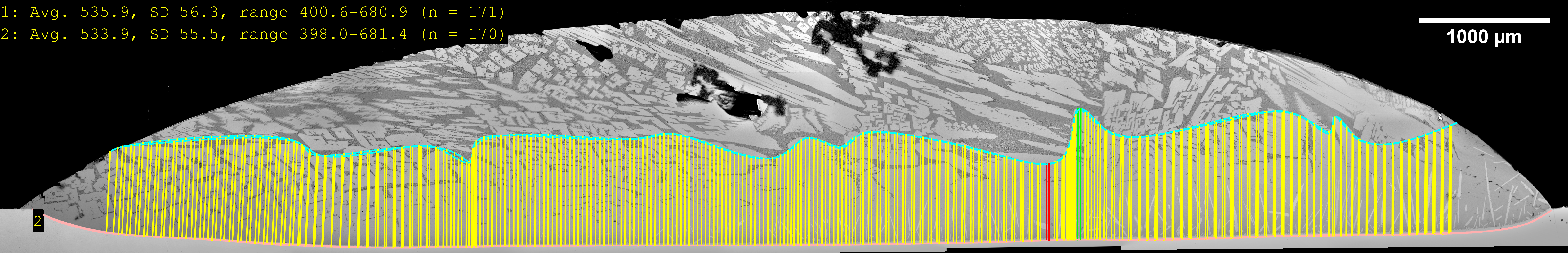

Supplement: Supplementary file 1 [file materials-18-05689-s001.zip › Supplementary Figures/Figures S2/Al 90s.tif]

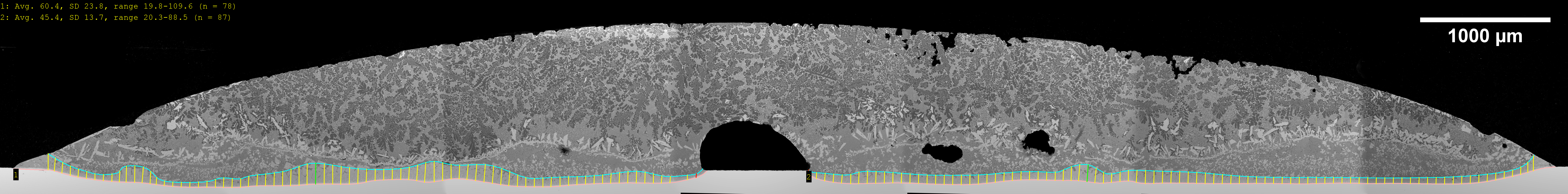

Supplement: Supplementary file 1 [file materials-18-05689-s001.zip › Supplementary Figures/Figures S2/Al-Ni 15s.tif]

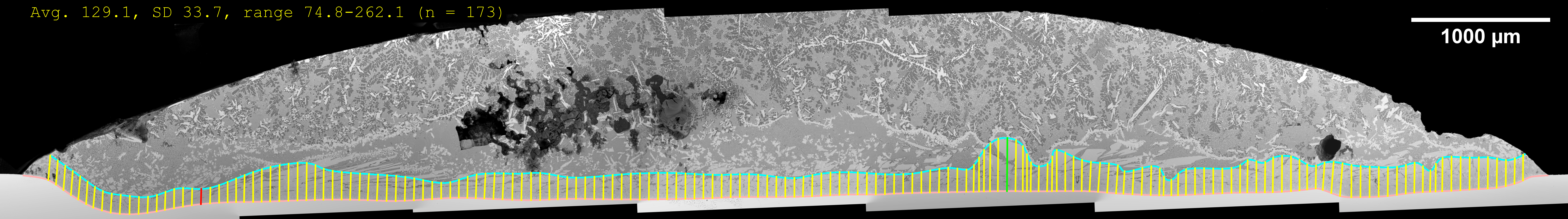

Supplement: Supplementary file 1 [file materials-18-05689-s001.zip › Supplementary Figures/Figures S2/Al-Ni 30s.tif]

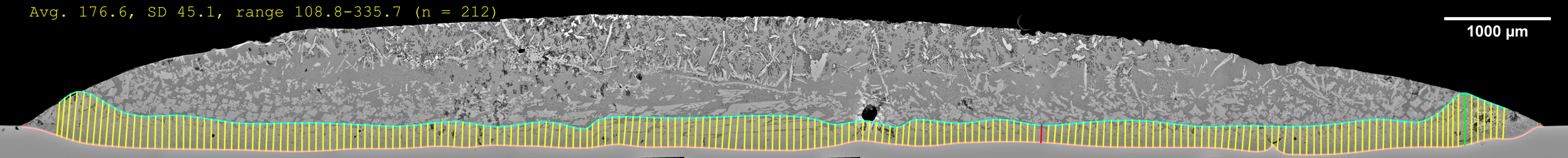

Supplement: Supplementary file 1 [file materials-18-05689-s001.zip › Supplementary Figures/Figures S2/Al-Ni 60s.tif]

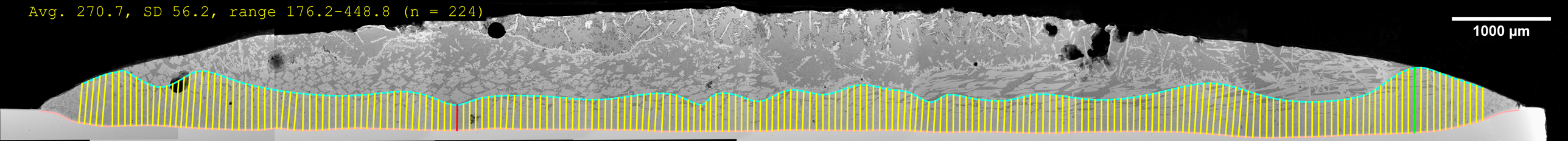

Supplement: Supplementary file 1 [file materials-18-05689-s001.zip › Supplementary Figures/Figures S2/Al-Ni 90s.tif]

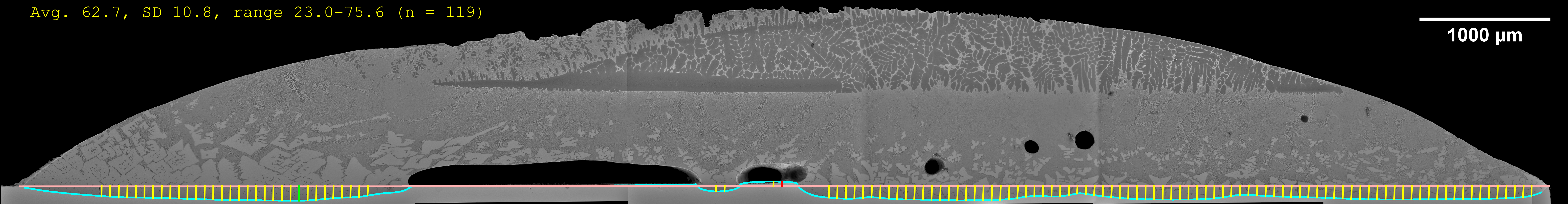

Supplement: Supplementary file 1 [file materials-18-05689-s001.zip › Supplementary Figures/Figures S3/Al 15s.tif]

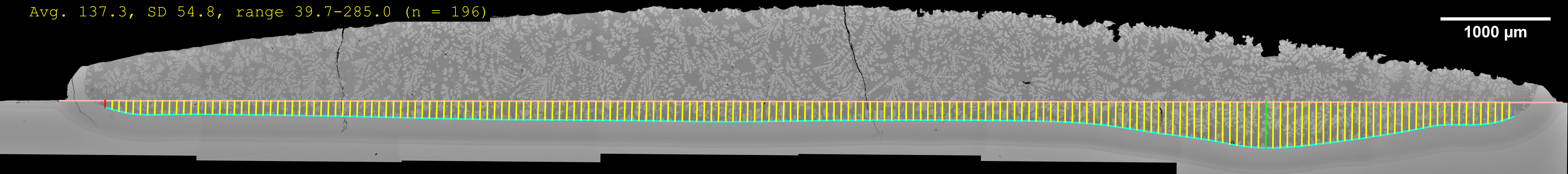

Supplement: Supplementary file 1 [file materials-18-05689-s001.zip › Supplementary Figures/Figures S3/Al 1800s.tif]

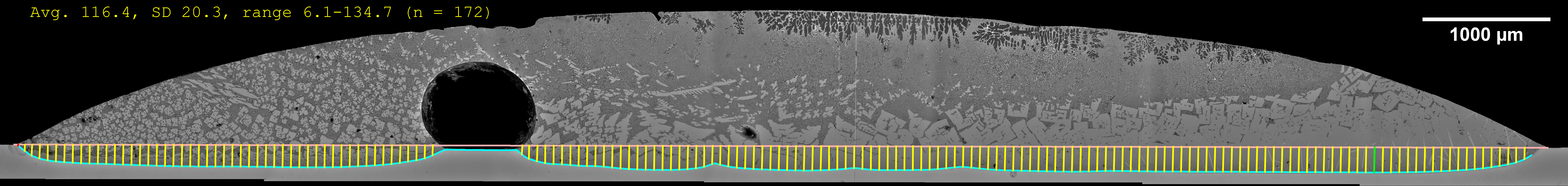

Supplement: Supplementary file 1 [file materials-18-05689-s001.zip › Supplementary Figures/Figures S3/Al 30s.tif]

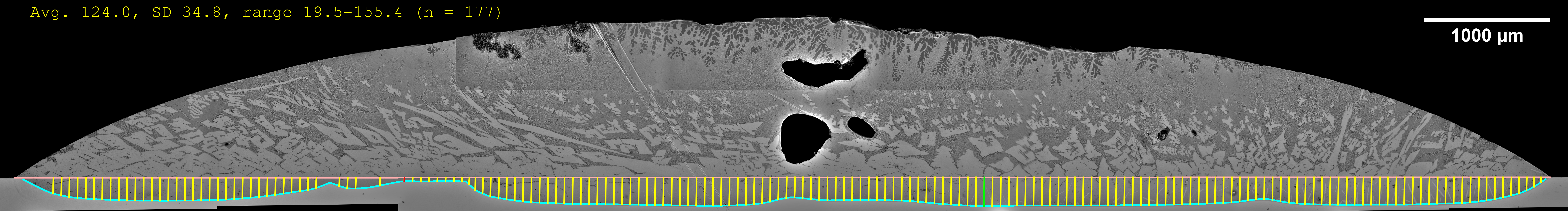

Supplement: Supplementary file 1 [file materials-18-05689-s001.zip › Supplementary Figures/Figures S3/Al 60s.tif]

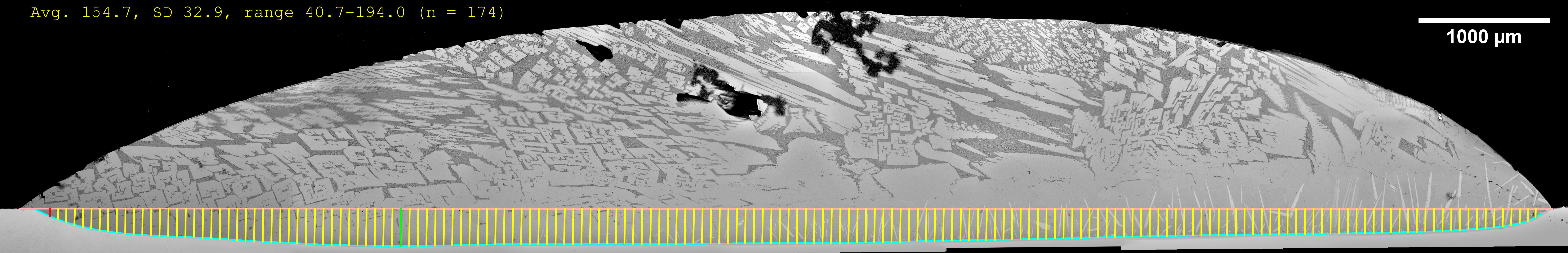

Supplement: Supplementary file 1 [file materials-18-05689-s001.zip › Supplementary Figures/Figures S3/Al 90s.tif]

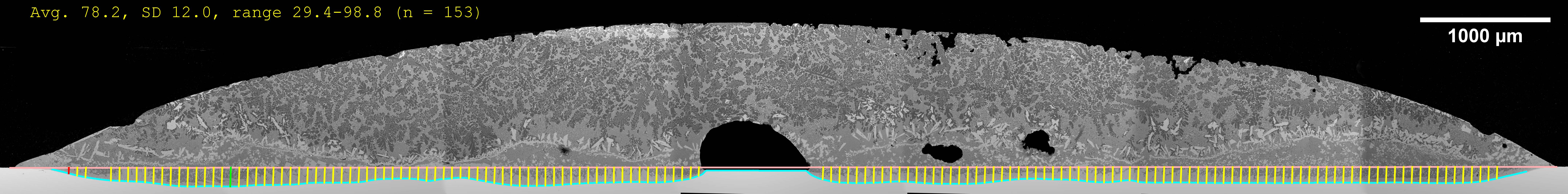

Supplement: Supplementary file 1 [file materials-18-05689-s001.zip › Supplementary Figures/Figures S3/Al-Ni 15s.tif]

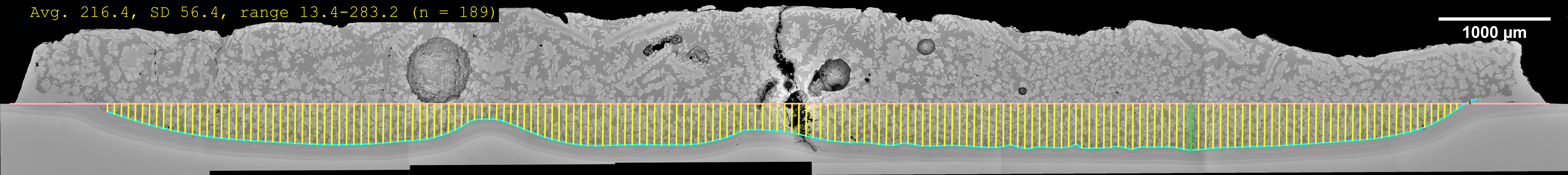

Supplement: Supplementary file 1 [file materials-18-05689-s001.zip › Supplementary Figures/Figures S3/Al-Ni 1800s.tif]

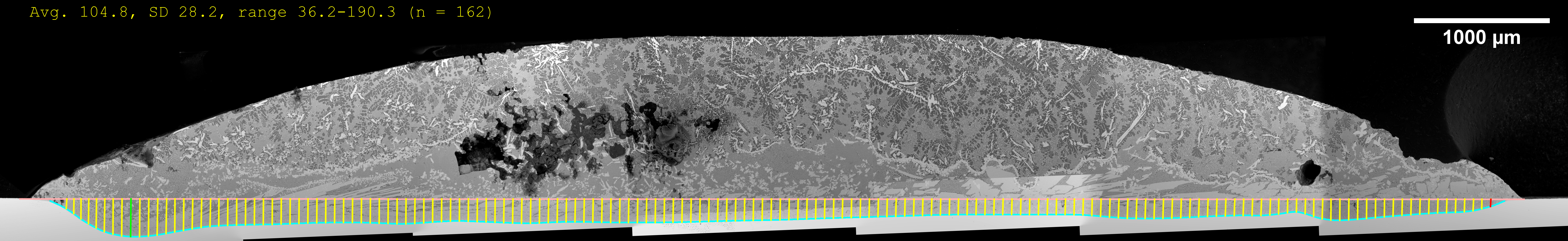

Supplement: Supplementary file 1 [file materials-18-05689-s001.zip › Supplementary Figures/Figures S3/Al-Ni 30s.tif]

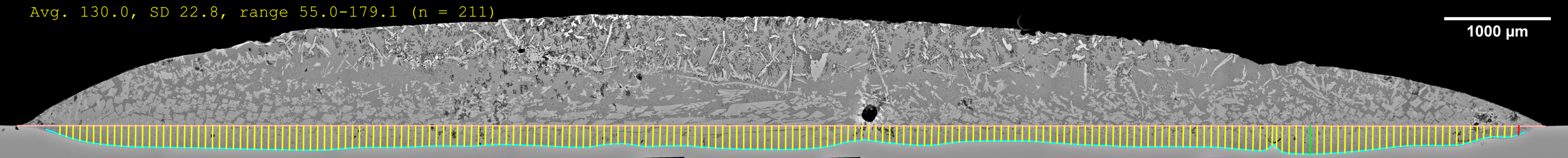

Supplement: Supplementary file 1 [file materials-18-05689-s001.zip › Supplementary Figures/Figures S3/Al-Ni 60s.tif]

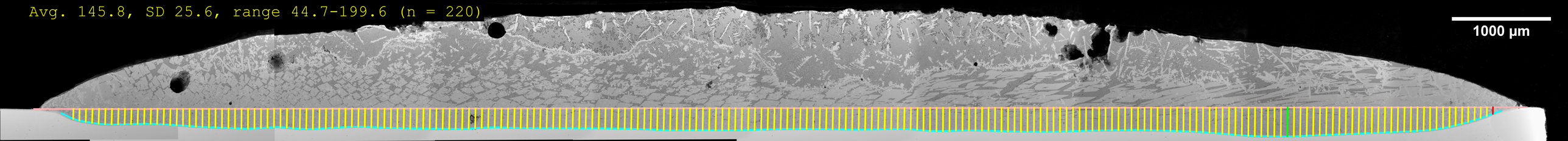

Supplement: Supplementary file 1 [file materials-18-05689-s001.zip › Supplementary Figures/Figures S3/Al-Ni 90s.tif]

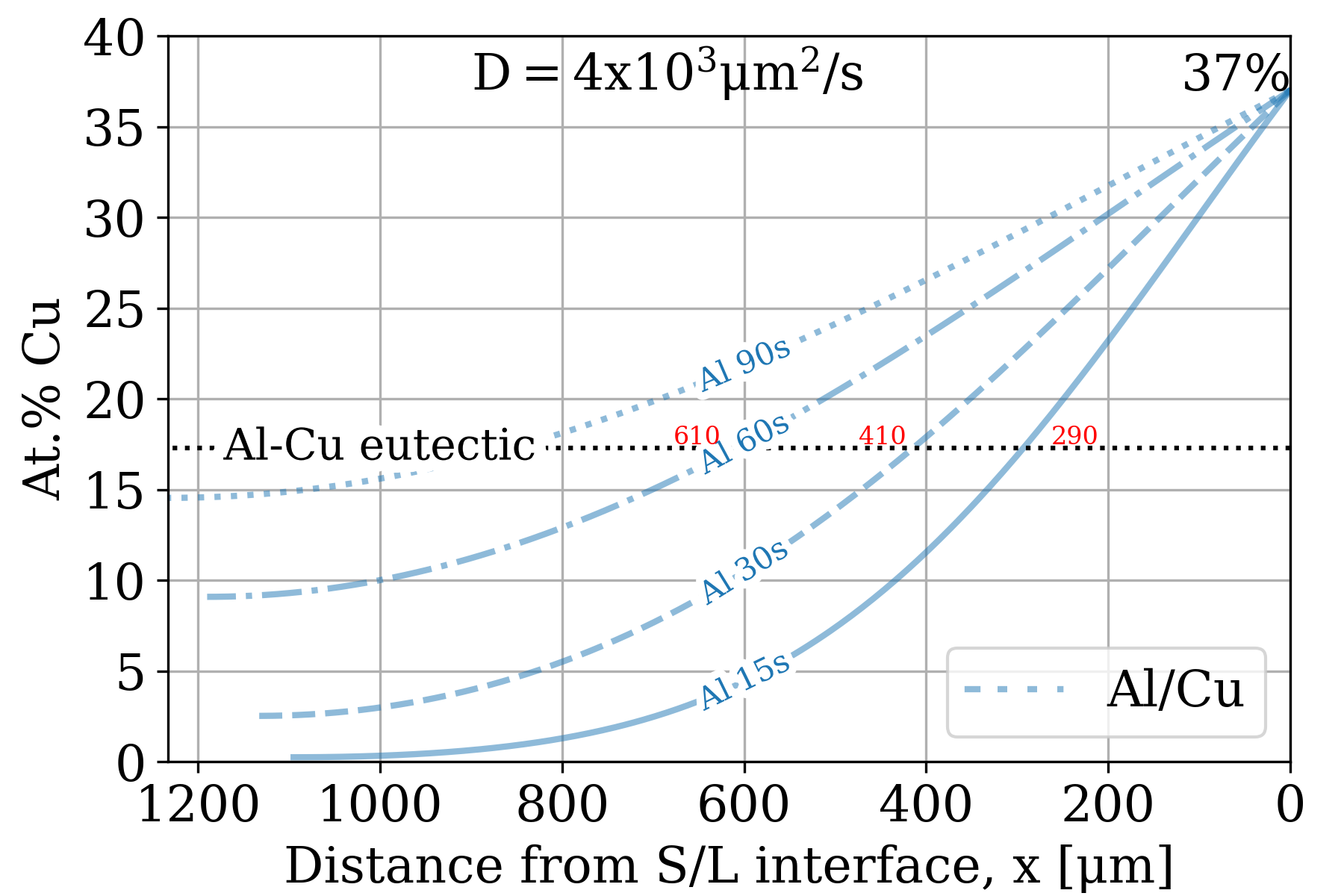

Supplement: Supplementary file 1 [file materials-18-05689-s001.zip › Supplementary Figures/Figures S4/Figure S4A.tif]

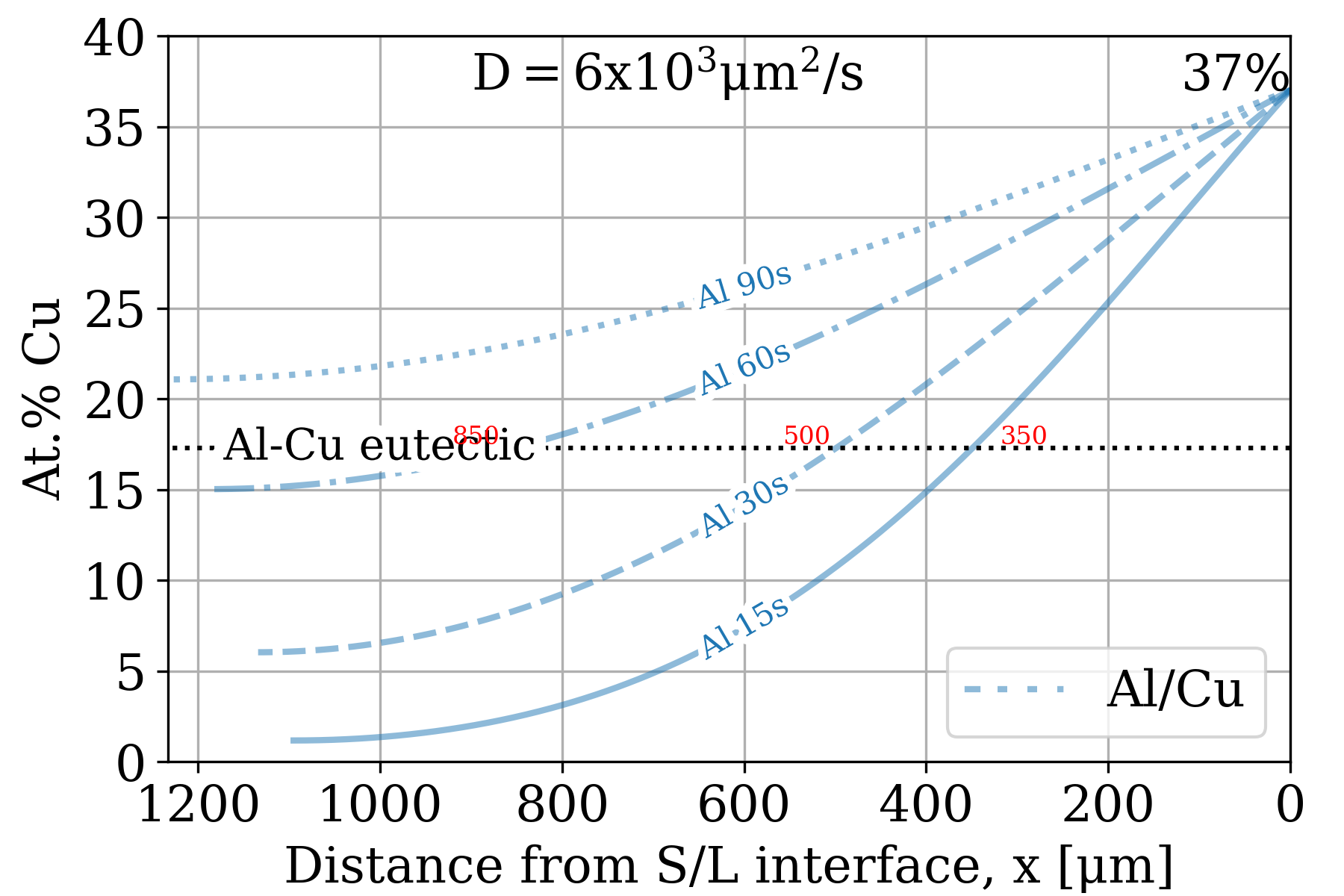

Supplement: Supplementary file 1 [file materials-18-05689-s001.zip › Supplementary Figures/Figures S4/Figure S4B.tif]

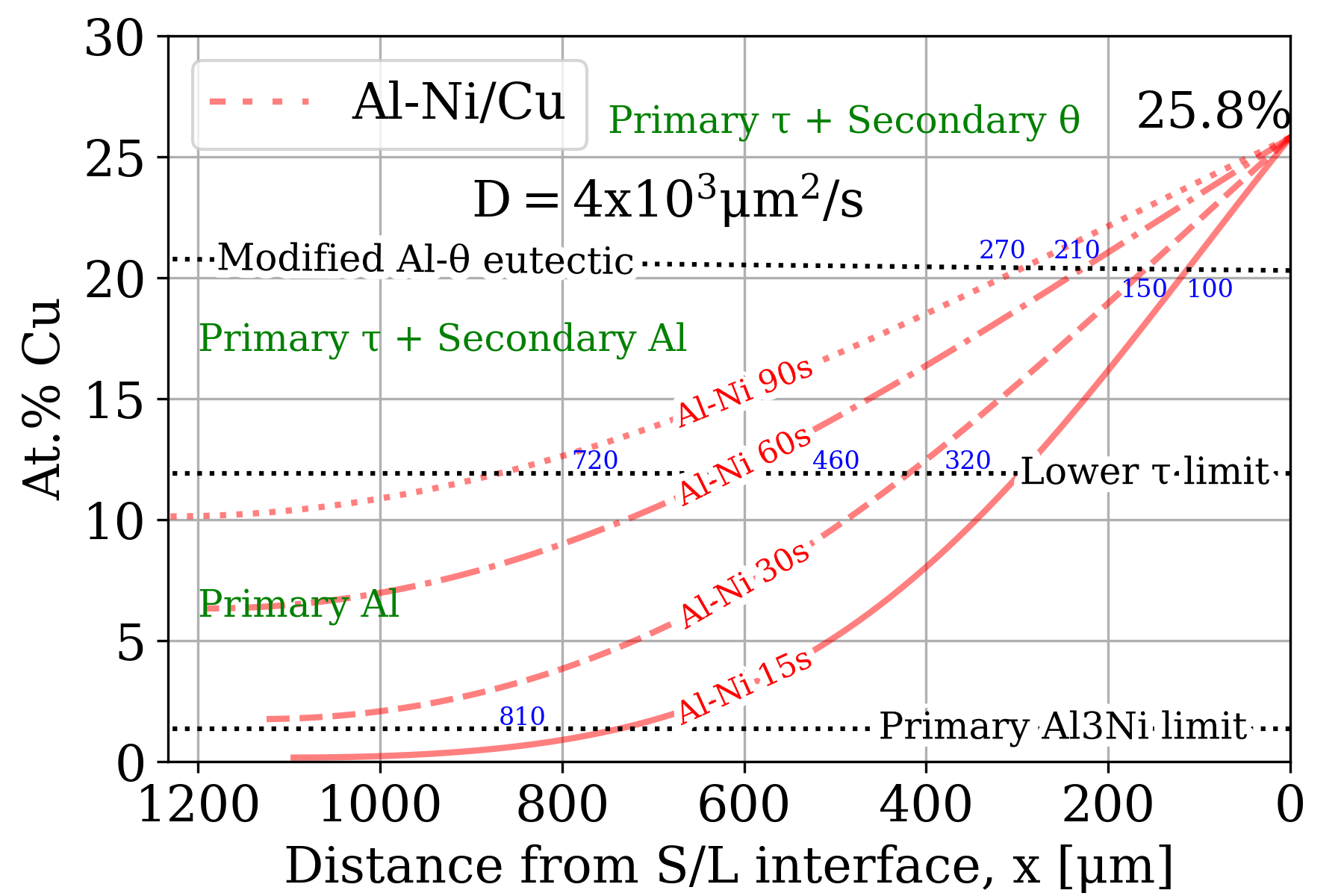

Supplement: Supplementary file 1 [file materials-18-05689-s001.zip › Supplementary Figures/Figures S4/Figure S4C.tif]

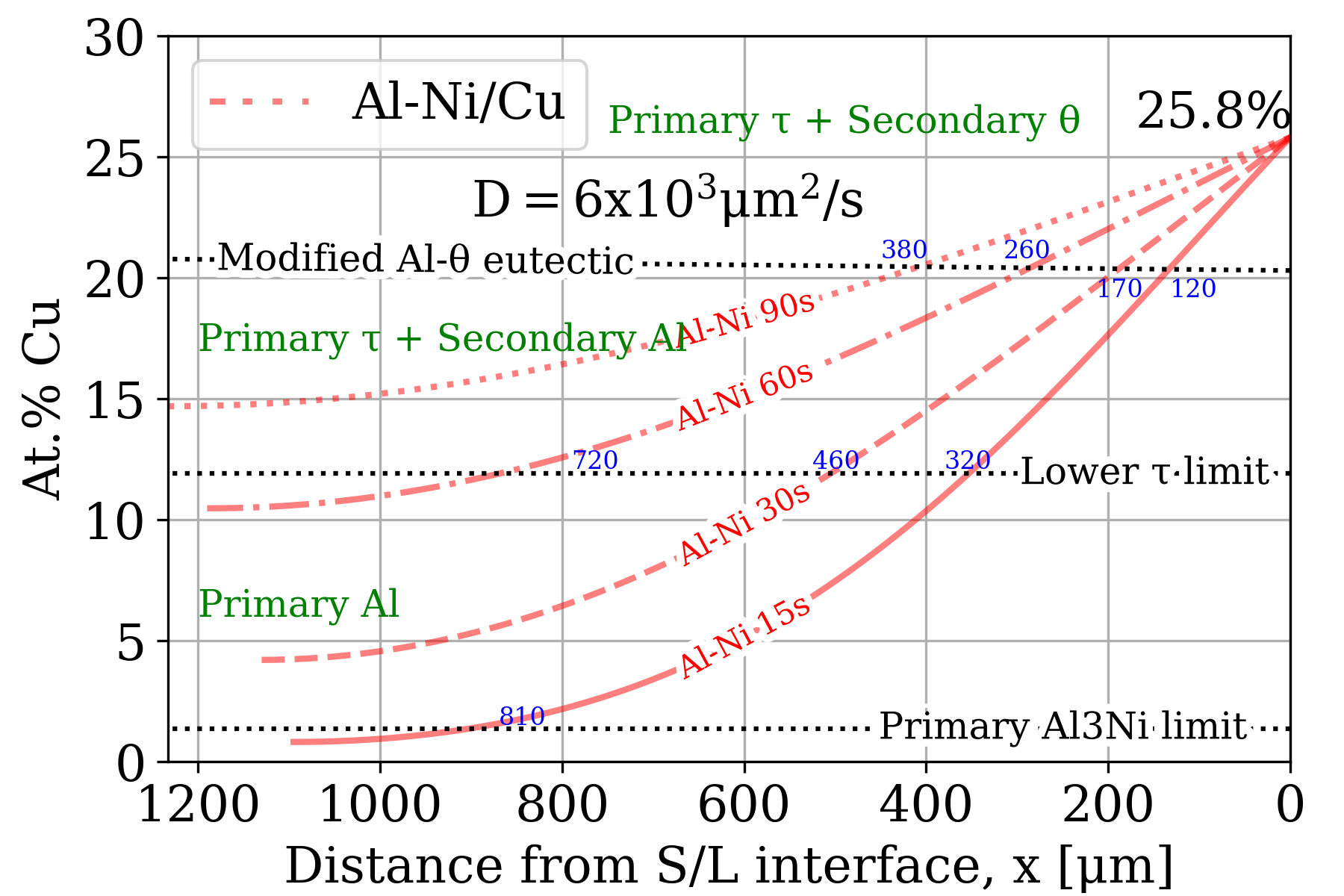

Supplement: Supplementary file 1 [file materials-18-05689-s001.zip › Supplementary Figures/Figures S4/Figure S4D.tif]

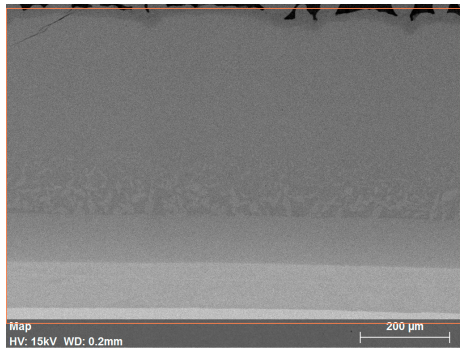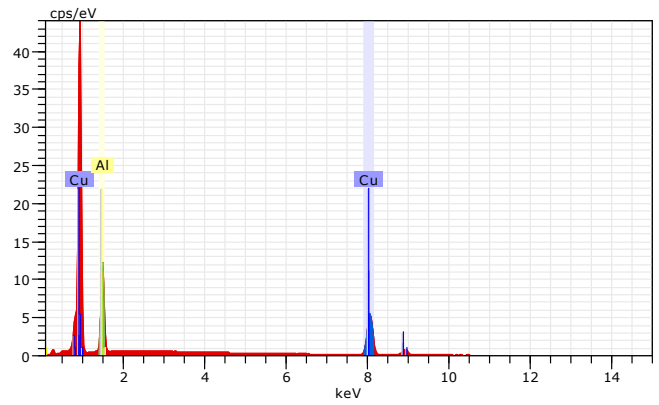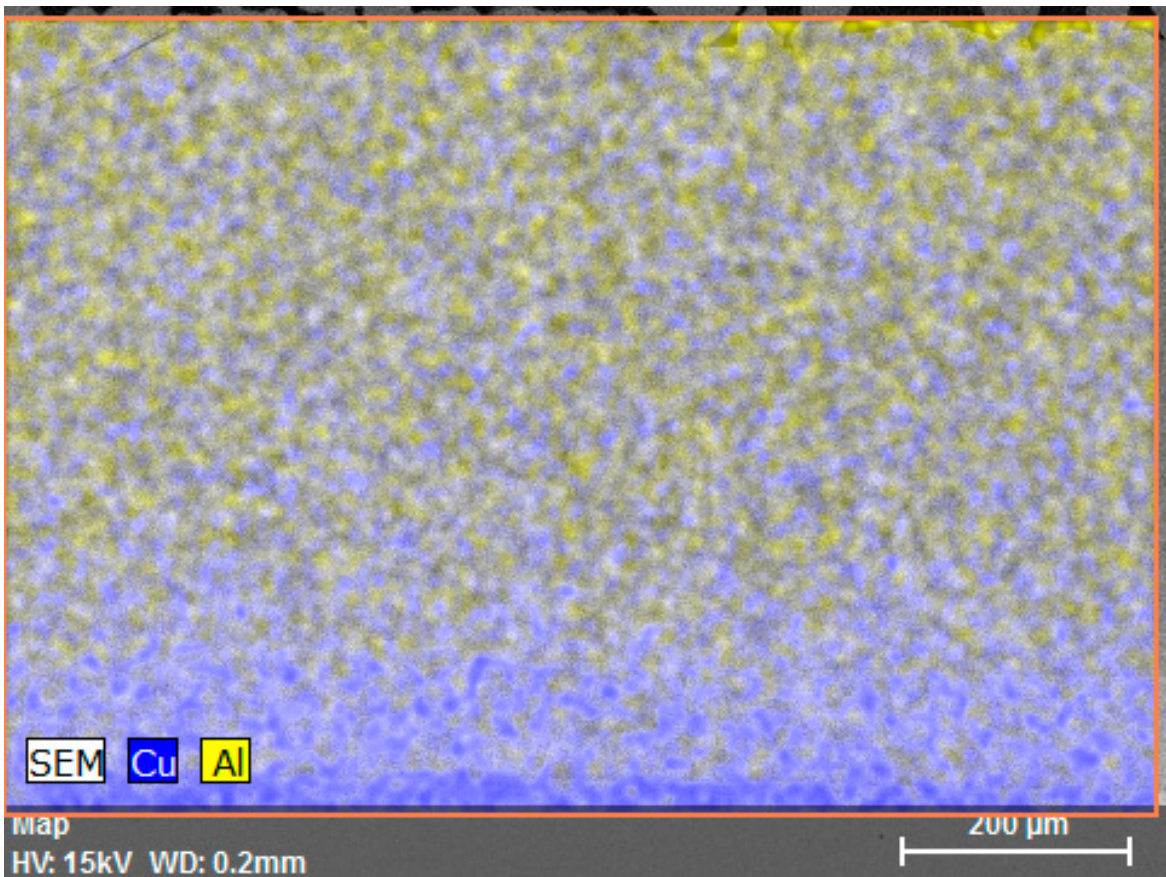

MapDate:05-Aug-25 4:45:59 PMImage size:480 x 360

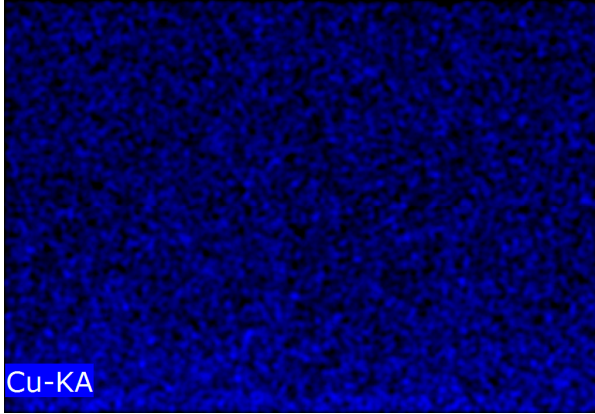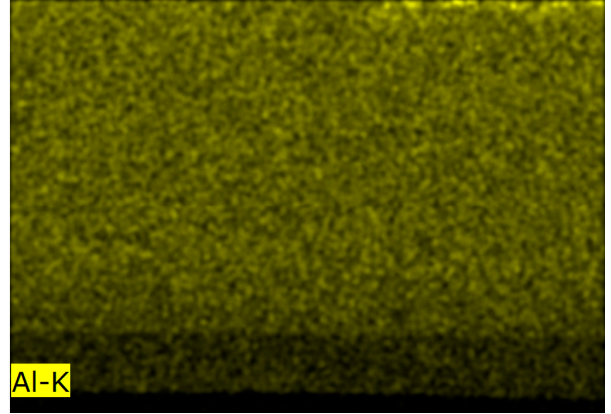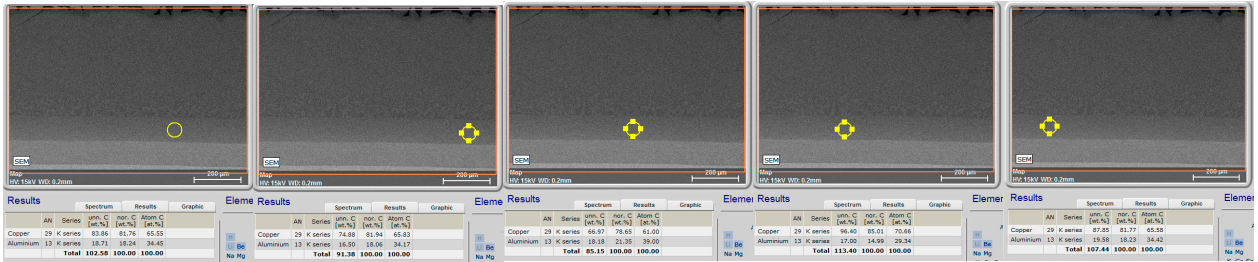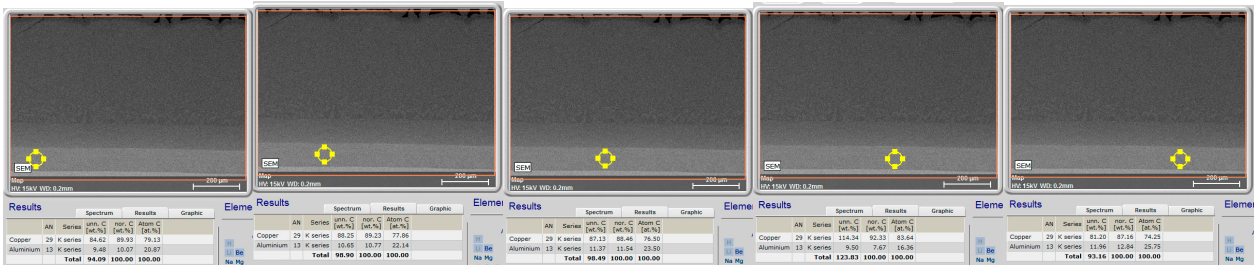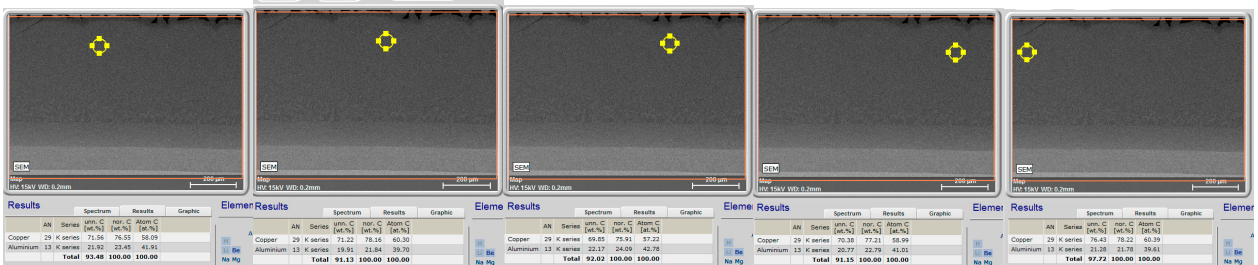

Supplement: Supplementary file 1 [file materials-18-05689-s001.zip › Supplementary Figures/Files S1/Al 1800s diffusion zone/EDS scans 2.pdf]

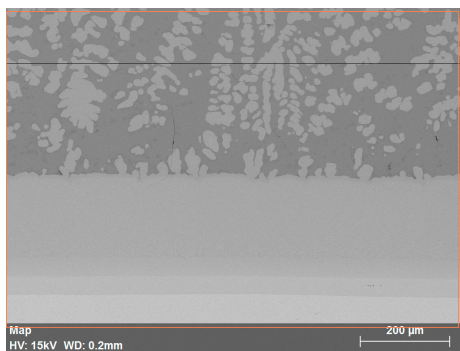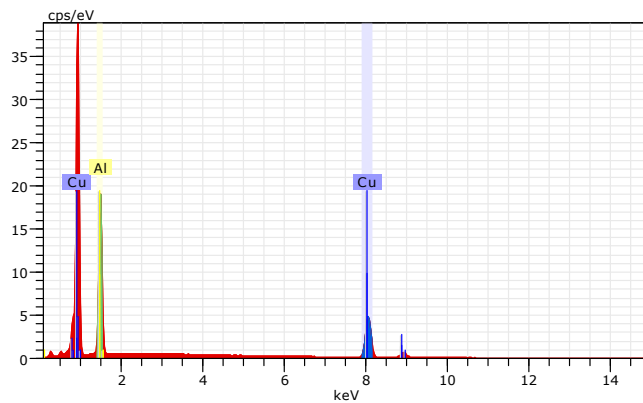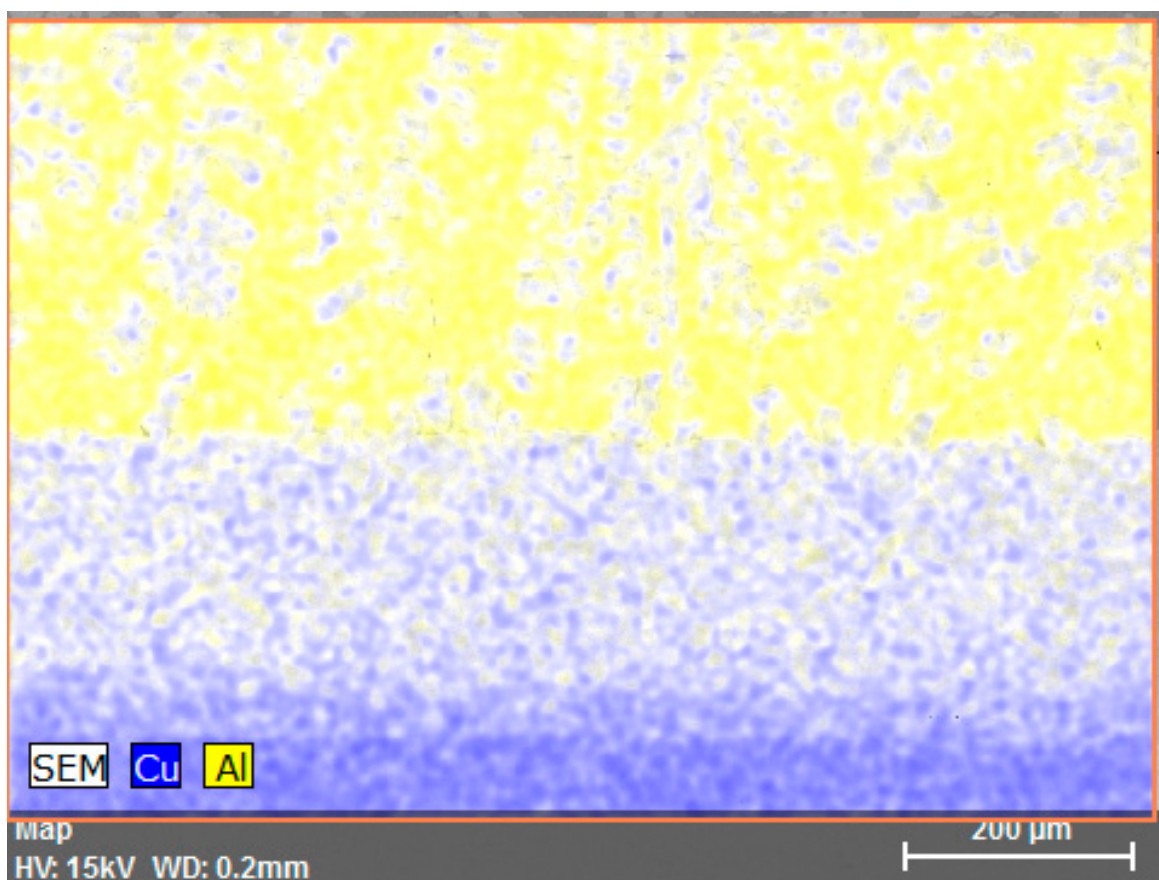

MapDate:05-Aug-25 4:30:38 PMImage size:480 x 360

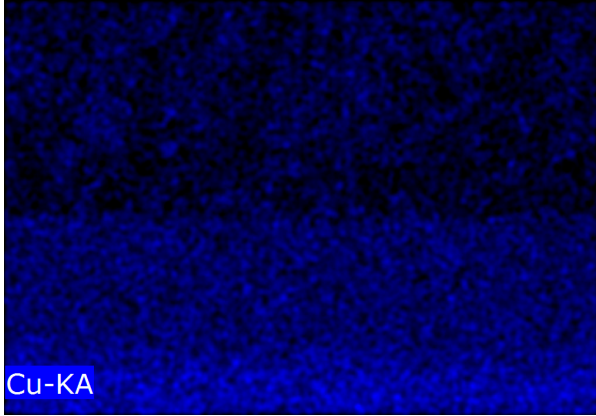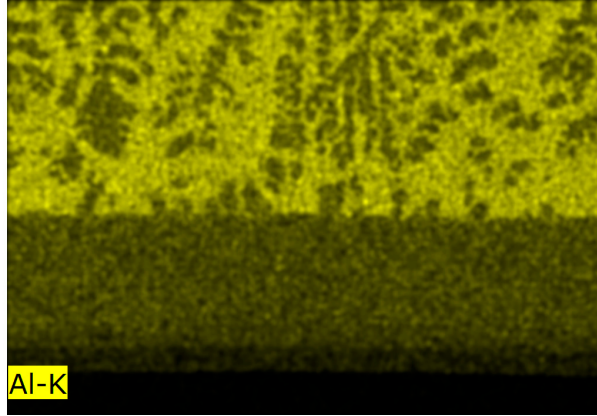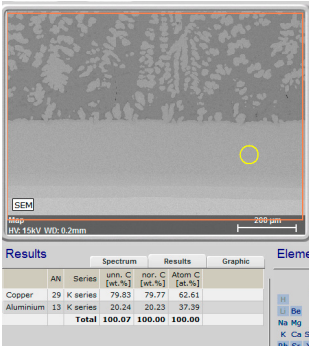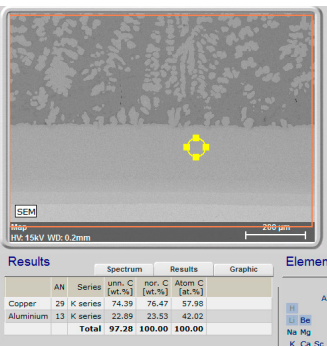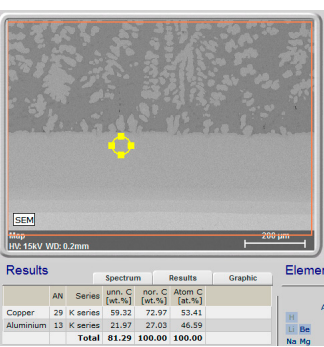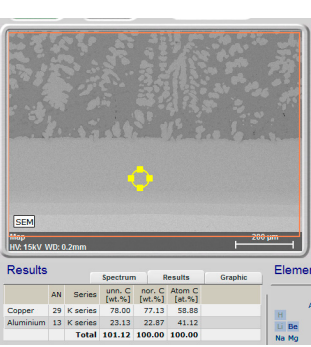

Supplement: Supplementary file 1 [file materials-18-05689-s001.zip › Supplementary Figures/Files S1/Al 1800s diffusion zone/EDS scans 3.pdf]

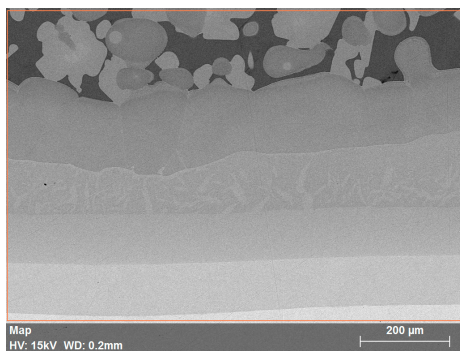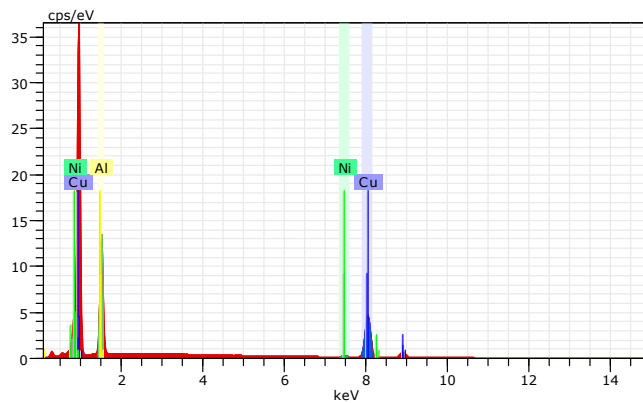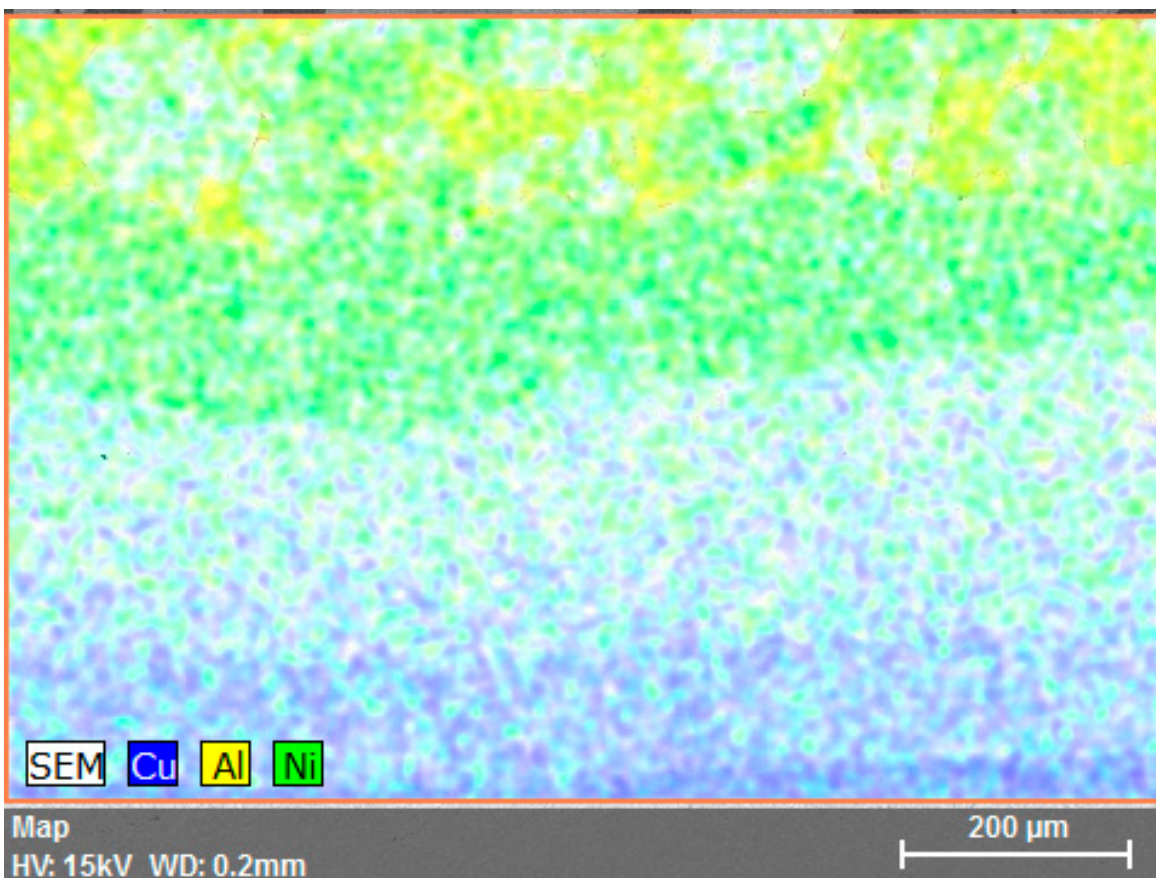

MapDate:07-Aug-25 5:59:52 PMImage size:480 x 360

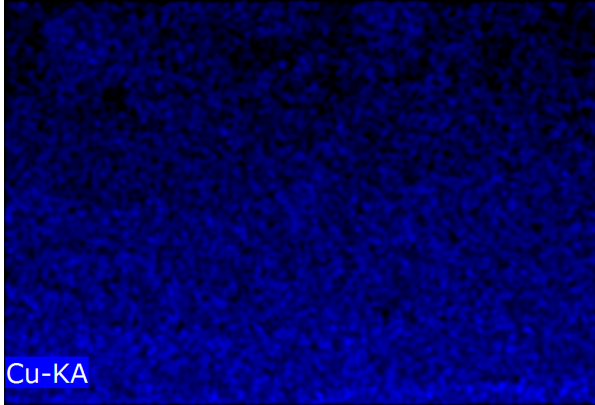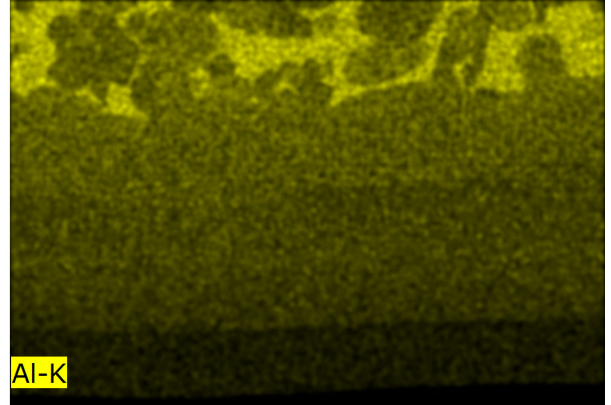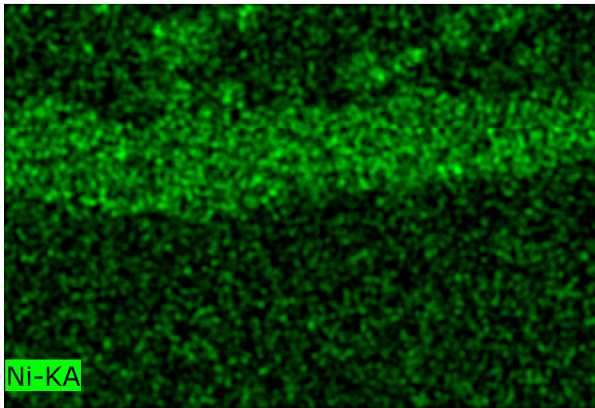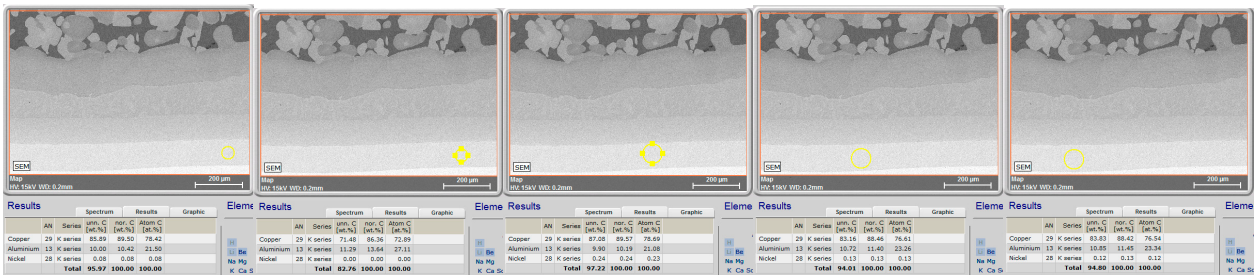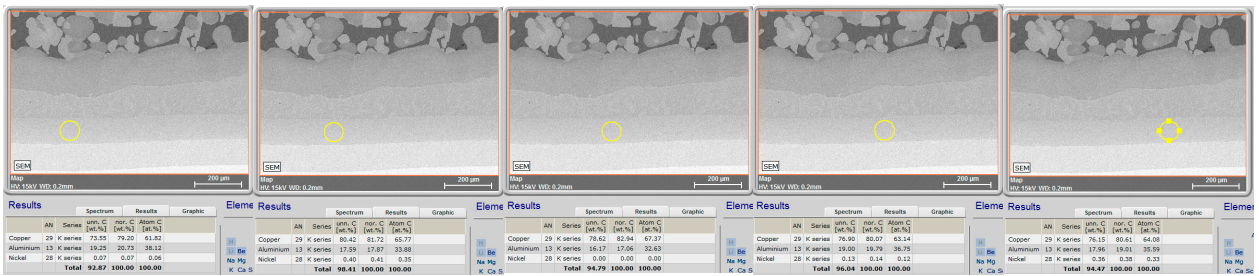

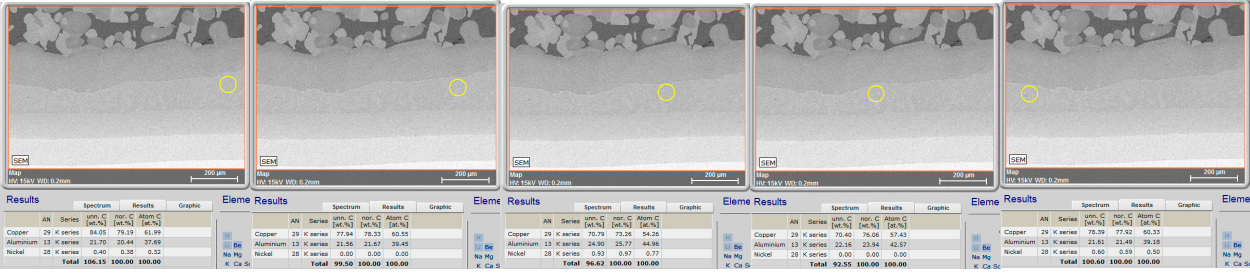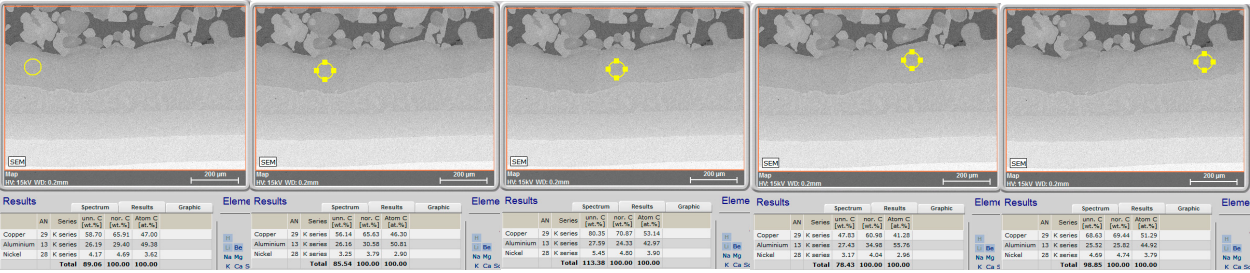

Supplement: Supplementary file 1 [file materials-18-05689-s001.zip › Supplementary Figures/Files S1/Al-Ni 1800s diffusion zone/EDS 3.pdf]
